# Dataset on causality analysis of chilling process in beef and pork carcasses using graphical modeling

**DOI:** 10.1016/j.dib.2020.106075

**Published:** 2020-07-25

**Authors:** Kumiko Kuzuoka, Kohji Kawai, Syunpei Yamauchi, Ayaka Okada, Yasuo Inoshima

**Affiliations:** a The United Graduate School of Veterinary Sciences, Gifu University Japan; b Toyohashi City Meat Hygiene Inspection Center, Department of Health, Toyohashi City Japan; c Laboratory of Food and Environmental Hygiene, Cooperative Department of Veterinary Medicine, Gifu University Japan; d Education and Research Center for Food Animal Health, Gifu University (GeFAH) Japan; e Joint Graduate School of Veterinary Sciences, Gifu University Japan

**Keywords:** Carcass temperature, Chilling process, Causality analysis, Graphical modeling, Hazard analysis and critical control point, Multivariate data analysis, Affector, Layer structure, fbo, food business operator, GM, graphical modeling

## Abstract

Appropriate control of carcass temperatures in slaughterhouses requires an accurate understanding of extrinsic and intrinsic factors present after slaughter and dressing. Therefore, we use large amounts of data required under the hazard analysis and critical control point system that are accumulated in daily business reports compiled by food business operators. This data aims to clarify the influencing factors or affectors of the chilling processes for beef and pork carcasses in a slaughterhouse using graphical modeling (GM), which is an explorative method in multivariate data analysis. GM has been widely used for statistical causality analysis in visual and flexible modeling. GM is carried out using the following parameters: outside temperature and humidity, number of carcasses in a chilling room on each operating day and during every afternoon of operation, time of sealing a chilling room, pre-set temperature in a chilling room, chilling room temperature at 16:30 on the day of slaughter and dressing and at 8:00 on the next day, and surface and core temperatures of carcasses. These parameters are set in a three-layered structure comprising (1) cause, (2) intermediate effect, and (3) effect. Covariance selection is performed to statistically eliminate spurious correlation. Path diagrams are drawn for beef and pork in GM for visualization. The data herein has contributed to the first attempt at the use of GM to statistically verify causality in the food manufacturing process. These data can be used to determine causality between carcass temperature and affectors in the chilling process via GM and thus minimize bias. Analyses of the present data are reported in the article “Chilling control of beef and pork carcasses in a slaughterhouse based on causality analysis by graphical modeling” [1].

**Specifications table**

**Table:** 

**Subject**	Safety, Risk, Reliability and Quality
**Specific subject area**	Food hygiene and biological risk analysis
**Type of data**	Table Figure Dataset
**How data were acquired**	Data were acquired from daily business reports written by FBOs in a slaughterhouse
**Data format**	Raw (The data written by FBOs were input to a spreadsheet in Excel 2010 (Microsoft, WA, USA)). Dates were set in rows as data ID, and surveillance items were arranged in the order of the operation process in the column of the matrix as a parameter of causal analysis. An array of each row was used as a data unit.) Analysed (When there was a blank space in the data unit due to an omission in the recording, the data units were excluded as missing values.) Filtered (The objects of measurement were two carcasses located on each corner of the exit side of each chilling room. The average temperatures of these two carcasses were used as data. Eight parameters concerning beef and ten parameters concerning pork from the surveillance items were selected as continuous variables.)
**Parameters for data collection**	The data were obtained for one year from April 2016 to March 2017. The surveillance items to be checked were designed by FBOs and had been empirically considered as affectors of the carcass chilling process. The surveillance items were recorded on all operating days. Carcass temperatures were regularly recorded once per week on Monday, or Tuesday in the case of a holiday or a maintenance day.
**Description of data collection**	Regarding representativeness bias, Kuzuoka and Kawai confirmed on-site how the FBOs recorded the data. We found that the FBOs had established a system in which one person did not record data. The management of the chilling process was rotated among multiple persons. Accuracy was guaranteed because the manager checked the daily records. Transparency was ensured because the records could be viewed by the stakeholders if they applied for viewing.
**Data source location**	Institution: The Higashi Mikawa Meat Distribution Center City/Town/Region: Toyohashi/ Akemi-cho/ 16–1 Country: Japan
**Data accessibility**	With the article Mendeley Data Repository name: [K*uzuoka*, K*umiko* (2020), K*uzuoka*_*et*_*al*_01, M*endeley* D*ata, v*1] Data identification number: [DOI: 10.17632/*yw*8*rgyt*98*z*.1] Direct URL to data: [https://data.mendeley.com/datasets/yw8rgyt98z/1]
**Related research article**	Author's name: Kumiko Kuzuoka, Kohji Kawai, Syunpei Yamauchi, Ayaka Okada, Yasuo Inoshima Title: Chilling control of beef and pork carcasses in a slaughterhouse based on causality analysis by graphical modeling Journal: Food Control 118 (2020) 107,353 https://doi.org/10.1016/j.foodcont.2020.107353

**Value of the data**

Biological risk in the meat industry, which mainly occurs in the form of foodborne pathogens of animal origin, is minimised by controlling the carcass temperature through the chilling process. The data are useful for analysing the causality of the influencing factors of the chilling process.
The following will significantly benefit from these data:

i Researchers statistically analysing the chilling process of carcasses after slaughter and dressing in slaughterhouses.
ii FBOs who manage the same chilling process.
iii Inspection authorities who are required to conduct inspection and guidance based on scientific evidence from the food industry.

The data will help identify factors that have the greatest effect on carcass temperature and those with no effect. Therefore, in further experiments relating to carcass chilling, unnecessary setting for comparison among groups can be eliminated, and costs and time can be reduced.
In the future, automatic control in the chilling process can be achieved by feeding data into artificial neural networks.

## 1 Data description

Numerous figures, tables, and datasets are provided. Figs. 1a to 1d represent examples of daily business records by FBOs: records of surveillance of the chilling process (Fig. 1a), measurement of surface temperature (Fig. 1b) of beef carcasses, and surface temperature (Fig. 1c) and core temperature (d) of pork carcasses. Because the daily business reports are written in Japanese, explanations in English are added in Figs. 1a–1d. Tables 1a–1d are English notation for Figs. 1a–1d.

**Fig. 1ab:**
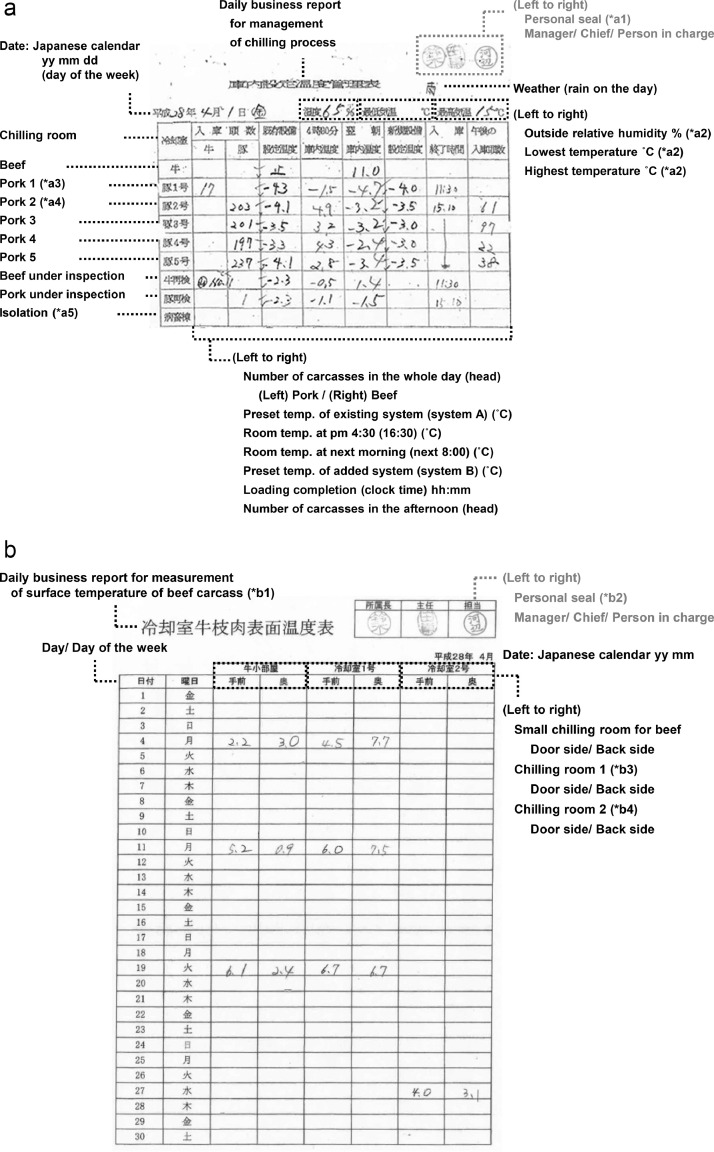
Representative example of daily business records by FBOs. **a.** Records of surveillance of chilling process. (*a1) A personal seal is more formal than a signature in Japan. In the Orient, the left is a higher rank. (*a2) The outside air temperature and humidity were measured at 12:00 of the slaughter day using a digital thermohygrometer. (*a3) Pork 1 was used for beef since 2015 (Japanese calendar Heisei 27) with the increase in the size of beef carcass in recent years, because the chilling room for beef was small. (*a4) Pork 2 was sometimes used for beef when Pork 1 is maintained. Pork 1 to 5 have same structures. (*a5) In cases of suspected legal infectious disease, the carcass is isolated in another facility on the same site. **b.** Measurement of surface temperature of beef carcass. (*b1) Carcass temperatures were regularly recorded once per week on Monday or Tuesday depending on whether Monday fell on a holiday and a maintenance day. (*b2) A personal seal is more formal than a signature in Japan. In the Orient, the left is a higher rank. (*b3) Chilling room 1 in Fig. 2. is Pork 1 in Fig. 1. (*b4) Chilling room 2 in Fig. 2. is Pork 2 in Fig. 1. Chilling room 2 was sometimes used for beef when Chilling room 1 is maintained. Chilling room 1 and 2 have same structures.

**Fig. 1cd:**
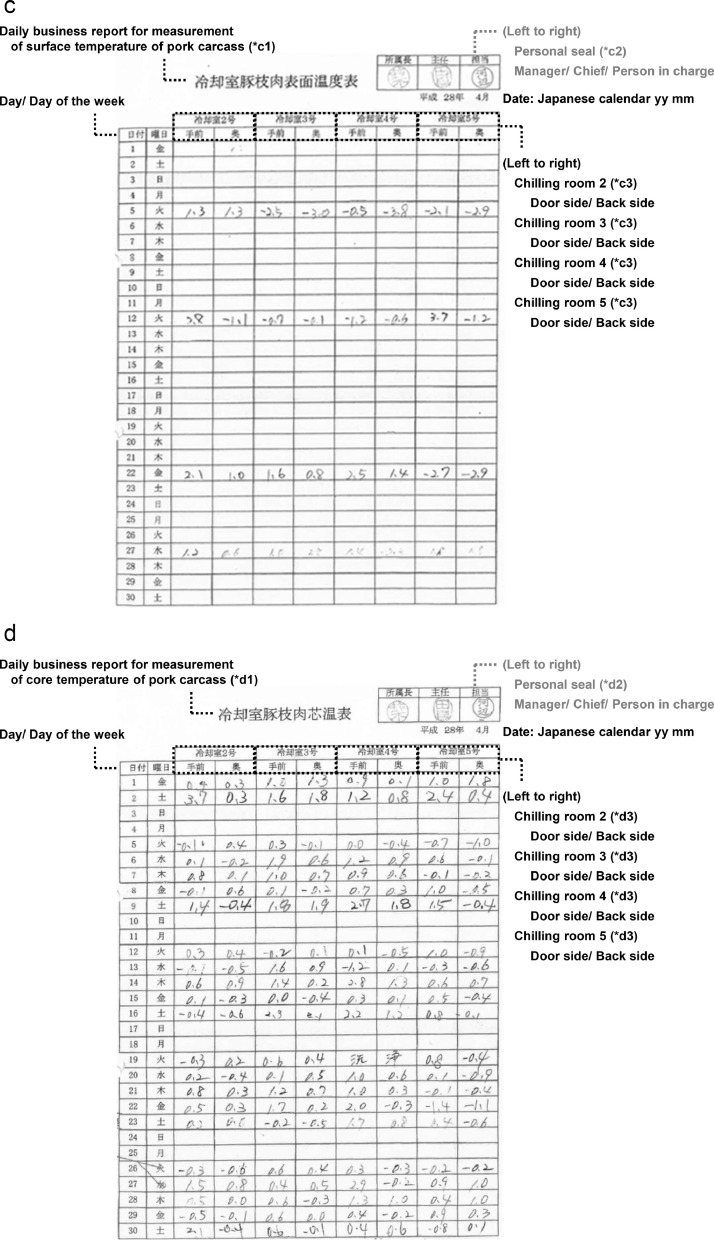
Representative example of daily business records by FBOs. **c.** Measurement of surface temperature of pork carcass. (*c1) Carcass temperatures were regularly recorded once per week on Monday or Tuesday depending on whether Monday fell on a holiday and a maintenance day. (*c2) A personal seal is more formal than a signature in Japan. In the Orient, the left is a higher rank. (*c3) Chilling room 2 to 5 have same structures. **d.** Measurement of core temperature of pork carcass. (*d1) At the request of stakeholders, core temperatures were experimentally recorded every operating day only in April 2016 (Japanese calendar Heisei 28). (*d2) A personal seal is more formal than a signature in Japan. In the Orient, the left is a higher rank. (*d3) Chilling room 2 to 5 have same structures.

**Table 1a:** English notation for Fig. 1a.

**Table 1b:** English notation for Fig. 1b.

**Table 1c:** English notation for Fig. 1c.

**Table 1d:** English notation for Fig. 1d.

Supplementary Dataset S1a and S1b in the public repository show the data processing of input and adjustment (https://data.mendeley.com/datasets/yw8rgyt98z/1). Tables 2a and 2b list the processed data of beef (*n* = 44) and pork (*n* = 44). Eight parameters associated with beef and 10 parameters associated with pork from the surveillance items were selected as continuous variables and were set in three layers as listed in Table 3. Tables 4a and 4b show correlation matrices, inverse matrices, and partial correlation matrices for beef and pork, respectively. Supplementary Figs. S1a and S1b in the public repository show the data processing of covariance selection for beef carcasses and pig carcasses (https://data.mendeley.com/datasets/yw8rgyt98z/1). Table 5 compares seasonal fluctuation and comprises the monthly sum of the number of carcass, operating days, and electric power consumption for the entire slaughterhouse. The total number of carcasses reported in Table 5 is given in standardized units of pork carcasses in which one beef carcass are considered equivalent to four pork carcasses. Table 6 is constructed from the data in Table 5 and shows the correlation matrix.

**Table 2a:** Processed data of beef (*n* = 44).

Sample ID	Outside temp.	Outside humidity	Carcass in the day	Loading completion	Preset temp.	Room temp. at 16:30	Room temp. at next 8:00	Surface temp.
	(°C)	(%)	(head)	(*1)	(°C)	(°C)	(°C)	(°C)
BC20160404	18	86	40	11.67	−6.2	1.0	−5.1	6.1
BC20160411	17	34	44	12.08	−6.7	1.1	−4.4	6.8
BC20160419	19	33	47	11.83	−7.2	1.4	−3.2	6.7
BC20160509	20	68	50	12.17	−7.2	5.4	−6.0	6.3
BC20160523	25	44	44	11.75	−6.7	4.1	−6.2	6.3
BC20160530	21	80	50	11.50	−7.2	4.8	−7.1	7.1
BC20160606	22	58	43	11.92	−6.7	4.6	−6.0	5.7
BC20160620	25	75	39	11.83	−6.2	3.8	−6.5	3.6
BC20160621	24	77	40	11.33	−6.2	3.4	−6.2	3.1
BC20160627	25	52	49	12.67	−5.7	4.3	−7.1	5.5
BC20160704	30	61	49	12.08	−7.2	3.5	−6.7	7.9
BC20160711	28	54	47	11.83	−7.2	2.7	−6.9	7.7
BC20160719	29	61	51	12.67	−7.7	3.5	−8.3	5.7
BC20160725	27	54	48	11.58	−7.2	3.7	−7.2	6.3
BC20160801	30	50	51	12.42	−7.7	4.3	−6.7	5.6
BC20160808	31	54	16	11.92	−4.2	−0.3	−3.5	3.2
BC20160816	28	62	51	11.67	−7.7	3.1	−8.1	6.9
BC20160823	29	65	44	12.67	−6.7	2.3	−7.5	6.4
BC20160829	31	45	50	12.17	−7.2	2.7	−6.4	5.3
BC20160906	27	76	50	11.33	−7.2	3.4	−5.1	6.6
BC20160912	27	68	42	11.75	−6.7	2.9	−7.4	4.4
BC20160926	25	77	43	12.42	−6.7	2.4	−7.8	6.4
BC20161003	25	84	49	11.83	−7.2	4.9	−6.5	7.3
BC20161011	23	50	48	11.92	−7.2	6.2	−6.5	5.6
BC20161017	21	81	44	12.67	−6.7	6.5	−6.6	5.9
BC20161024	21	39	44	12.00	−6.7	5.0	−5.9	6.1
BC20161031	19	50	40	12.25	−6.2	4.3	−7.1	4.8
BC20161101	19	49	40	12.50	−6.2	4.1	−5.7	5.6
BC20161107	17	43	48	12.42	−7.2	6.5	−7.1	7.3
BC20161114	17	63	47	12.33	−7.2	2.3	−7.4	7.6
BC20161128	15	35	50	12.58	−7.2	5.6	−6.6	7.3
BC20161205	16	49	50	11.83	−7.2	5.8	−8.8	8.7
BC20161212	11	36	50	12.25	−7.2	5.0	−7.5	8.9
BC20161219	13	48	27	12.00	−5.2	−1.0	−6.2	4.4
BC20160110	12	46	49	12.92	−7.2	5.0	−6.9	7.3
BC20160116	7	48	44	12.50	−6.7	5.0	−6.1	5.5
BC20160123	8	43	34	11.75	−5.7	4.0	−5.9	5.3
BC20160130	11	72	45	12.08	−6.7	5.0	−6.4	8.5
BC20160206	10	51	42	12.75	−6.7	5.1	−6.4	6.4
BC20160227	12	36	40	12.00	−6.2	4.3	−6.1	5.3
BC20160306	12	69	46	13.00	−7.2	4.0	−7.3	6.1
BC20160313	12	61	41	11.67	−6.7	2.3	−6.9	6.7
BC20160321	13	60	48	12.08	−7.2	3.2	−7.7	6.8
BC20160327	13	39	45	12.00	−6.7	5.3	−6.0	6.4

(*1) Conversion to continuous variable (written as hh+mm/60).

**Table 2b:** Processed data of pork (*n* = 44).

Sample ID	Outside temp.	Outside humidity	Carcass in the day	Carcass after noon	Loading completion	Preset temp.	Room temp. at 16:30	Room temp. at next 8:00	Surface temp.	Core temp.
	(°C)	(%)	(head)	(head)	(*1)	(°C)	(°C)	(°C)	(°C)	(°C)
PC20160404	18	86	110	0	12.58	−2.7	−3.8	−2.1	−2.8	0.1
PC20160411	17	34	115	18	14.50	−2.8	−2.9	−2.2	−0.4	−0.1
PC20160421	18	81	169	95	15.83	−3.2	2.6	−3.7	1.2	1.0
PC20160426	21	51	242	20	15.58	−4.2	0.4	−4.0	1.3	0.5
PC20160505	23	29	262	88	16.00	−4.7	3.1	−4.8	−2.5	0.2
PC20160510	20	77	229	30	15.50	−3.2	1.7	−3.7	−4.9	−0.1
PC20160523	25	44	119	0	12.42	−2.7	−2.8	−3.5	−7.1	−0.5
PC20160530	21	80	113	0	12.58	−2.7	−2.2	−3.0	−4.7	−0.3
PC20160607	22	67	165	31	14.50	−2.8	0.9	−2.9	−4.6	0.3
PC20160620	25	75	214	33	13.83	−3.2	1.3	−3.4	−5.2	0.3
PC20160708	27	71	201	95	16.00	−3.3	3.9	−2.0	−6.4	0.0
PC20160711	28	54	211	0	13.00	−3.1	−0.8	−3.4	−4.3	−0.2
PC20160719	29	61	217	90	15.67	−3.3	4.7	−3.6	−5.1	0.4
PC20160726	25	76	186	28	14.67	−3.1	1.5	−3.1	−4.8	−0.7
PC20160801	30	50	210	0	12.50	−3.1	0.3	−2.8	−5.7	−0.6
PC20160808	31	54	109	18	14.00	−2.8	2.3	−2.9	−3.8	0.5
PC20160816	28	62	220	76	15.17	−3.3	0.8	−3.7	−3.7	0.1
PC20160830	27	46	163	36	14.50	−2.8	2.3	−3.0	−5.1	−0.7
PC20160905	27	86	109	19	14.33	−2.3	1.7	−2.7	−5.1	−0.2
PC20160913	26	86	170	57	15.17	−3.2	4.0	−3.4	−4.9	−0.5
PC20160920	24	78	228	63	16.17	−3.7	−3.3	−4.0	−6.1	−0.3
PC20160926	25	77	124	28	14.50	−2.8	3.2	−2.8	−5.1	−0.4
PC20161011	23	50	234	130	15.33	−3.8	5.8	−3.6	−5.3	0.2
PC20161017	21	81	137	38	15.92	−2.8	1.8	−2.5	−5.5	−0.2
PC20161025	19	63	200	103	15.08	−3.3	5.0	−3.6	−4.3	0.1
PC20161102	17	43	258	113	15.92	−4.1	2.1	−4.1	−2.2	0.0
PC20161107	17	43	115	27	14.75	−2.8	2.4	−3.0	−2.3	0.1
PC20161114	17	63	155	17	14.42	−2.8	−0.3	−2.7	−2.7	−0.5
PC20161121	18	63	233	56	15.25	−4.3	5.3	−4.0	−2.7	0.2
PC20161128	15	35	132	0	12.42	−2.7	−1.4	−3.6	−0.3	−0.4
PC20161206	16	40	165	62	14.92	−3.1	2.6	−3.6	−2.1	0.0
PC20161213	11	66	229	103	15.83	−3.8	3.4	−3.3	−5.0	−0.1
PC20161219	13	48	154	43	15.17	−2.8	3	−2.2	−0.8	−0.2
PC20160104	12	46	199	68	16.17	−3.2	4.8	−4.1	−4.3	0.2
PC20160111	12	41	228	84	16.00	−3.8	5.3	−3.9	−3.5	0.1
PC20160116	7	48	154	38	15.08	−2.8	4.3	−3.1	−2.3	−0.1
PC20160123	8	43	127	14	14.50	−2.7	1.4	−2.6	−3.3	−0.7
PC20160206	10	51	236	0	12.67	−3.2	3.6	−3.3	−3.2	0.2
PC20160213	9	40	144	0	12.58	−2.8	−2.3	−2.8	−2.1	−0.4
PC20160227	12	36	126	0	12.67	−2.7	−2.2	−2.8	−0.4	0.0
PC20160306	12	69	234	0	15.83	−3.2	2.5	−3.4	−3.5	0.4
PC20160314	14	37	196	52	16.00	−4.3	3.1	−4.4	−4.5	−0.2
PC20160321	13	60	261	154	16.33	−4.8	4.9	−5.4	−2.9	1.3
PC20160327	13	39	214	0	12.67	−3.1	2.7	−3.5	−3.6	−0.1

(*1) Conversion to continuous variable (written as hh+mm/60).

**Table 3:** Setting of layer structures and parameters.

Layers	Notations	Parameters
*Beef*		

First layer: Cause	Outdoor_temp ( °C)	Outdoor temperature at noon on the day of slaughtering and dressing
	Outdoor_humidity (%)	Outdoor humidity at noon on the day of slaughtering and dressing
	Carcasses_in_the_day (head)	Number of carcasses loaded in the chilling room in the whole day

Second (Middle) layer: Intermediate effect	Loading_completion (clock time)	Clock time of completion loading all carcasses in the chilling room
	Preset_temp ( °C)	Preset temperature of the chilling room
	Room_temp_at_16:30 ( °C)	Room temperature of the chilling room at 16:30 on the day of slaughtering and dressing
	Room_temp_next_8:00 ( °C)	Room temperature of the chilling room at 8:00 on the day after slaughtering and dressing

Third layer: Effect	Surface_temp ( °C)	Average of surface temperatures of two carcasses on shoulder

*Pork*		

First layer: Cause	Outdoor_temp ( °C)	Same with beef
	Outdoor_humidity (%)	Same with beef
	Carcasses_in_the_day (head)	Same with beef

Second (Middle) layer: Intermediate effect	Carcasses_in_pm (head)	Number of carcasses loaded in a chilling room in the afternoon
	Loading_completion (clock time)	Same with beef
	Preset_temp ( °C)	Same with beef
	Room_temp_at_16:30 ( °C)	Same with beef
	Room_temp_next_8:00 ( °C)	Same with beef

Third layer: Effect	Surface_temp ( °C)	Average of surface temperatures of two carcasses on the gluteal region
	Core_temp ( °C)	Average of core temperatures of two carcasses at a depth of 7 cm on the gluteal region

**Table 4a:** Matrixes required for calculation of covariance selection of beef (No unit in matrixes).

Correlation matrix	Outside temp.	Outside humidity	Carcass in the day	Loading completion	Preset temp.	Room temp. at 16:30	Room temp. at next 8:00	Surface temp.
Outside tem.	1	0.3059	0.0976	−0.2262	−0.1004	−0.2297	−0.0291	−0.2527
Outside humidity	0.3059	1	0.0253	−0.1984	−0.0422	−0.0626	−0.2021	−0.1009
Carcass in the day	0.0976	0.0253	1	0.1498	−0.9156	0.5443	−0.4748	0.6334
Loadig completion	−0.2262	−0.1984	0.1498	1	−0.1055	0.2565	−0.2515	0.1250
Preset temp.	−0.1004	−0.0422	−0.9156	−0.1055	1	−0.4395	0.4607	−0.6230
Room temp. at 16:30	−0.2297	−0.0626	0.5443	0.2565	−0.4395	1	−0.3656	0.3238
Room temp. at next 8:00	−0.0291	−0.2021	−0.4748	−0.2515	0.4607	−0.3656	1	−0.3213
Surface temp.	−0.2527	−0.1009	0.6334	0.1250	−0.6230	0.3238	−0.3213	1

Inverse matrix	Outside temp.	Outside humidity	Carcass in the day	Loading completion	Preset temp.	Room temp. at 16:30	Room temp. at next 8:00	Surface temp.
Outside tem.	1.5558	−0.2888	−0.9338	0.2044	0.0245	0.5703	0.0407	0.7736
Outside humidity	−0.2888	1.2188	0.1059	0.2422	0.0378	0.0110	0.3646	0.0897
Carcass in the day	−0.9338	0.1059	8.1871	−0.2024	5.8989	−1.5891	0.1647	−1.1433
Loadig completion	0.2044	0.2422	−0.2024	1.2093	−0.1780	−0.1249	0.3276	0.0878
Preset temp.	0.0245	0.0378	5.8989	−0.1780	6.5947	−0.5270	−0.3142	0.4740
Room temp. at 16:30	0.5703	0.0110	−1.5891	−0.1249	−0.5270	1.7720	0.2327	0.3401
Room temp. at next 8:00	0.0407	0.3646	0.1647	0.3276	−0.3142	0.2327	1.5017	0.1131
Surface temp.	0.7736	0.0897	−1.1433	0.0878	0.4740	0.3401	0.1131	2.1392

Partial correlation matrix	Outside temp.	Outside humidity	Carcass in the day	Loading completion	Preset temp.	Room temp. at 16:30	Room temp. at next 8:00	Surface temp.
Outside tem.	.	0.2097	0.2616	−0.1490	−0.0076	−0.3435	−0.0266	−0.4240
Outside humidity	0.2097	.	−0.0335	−0.1995	−0.0133	−0.0075	−0.2695	−0.0556
Carcass in the day	0.2616	−0.0335	.	0.0643	−0.8028	0.4172	−0.0470	0.2732
Loadig completion	−0.1490	−0.1995	0.0643	.	0.0630	0.0853	−0.2431	−0.0546
Preset temp.	−0.0076	−0.0133	−0.8028	0.0630	.	0.1542	0.0998	−0.1262
Room temp. at 16:30	−0.3435	−0.0075	0.4172	0.0853	0.1542	.	−0.1427	−0.1747
Room temp. at next 8:00	−0.0266	−0.2695	−0.0470	−0.2431	0.0998	−0.1427	.	−0.0631
Surface temp.	−0.4240	−0.0556	0.2732	−0.0546	−0.1262	−0.1747	−0.0631	.

**Table 4b:** Matrixes required for calculation of covariance selection of pork (No unit in matrixes).

Correlation matrix	Outside temp.	Outside humidity	Carcass in the day	Carcass after noon	Loading completion	Preset temp.	Room temp. at 16:30	Room temp. at next 8:00	Surface temp.	Core temp.
Outside temp.	1	0.3892	−0.0157	0.0254	−0.0386	0.0719	−0.0956	0.0877	−0.4842	−0.0978
Outside humidity	0.3892	1	−0.0683	0.0491	0.1453	0.1702	−0.0166	0.2089	−0.3783	0.0685
Carcass in the day	−0.0157	−0.0683	1	0.5535	0.4880	−0.8188	0.4692	−0.6878	−0.0844	0.3874
Carcass after noon	0.0254	0.0491	0.5535	1	0.7496	−0.6653	0.6274	−0.5274	−0.0758	0.4847
Loading completion	−0.0386	0.1453	0.4880	0.7496	1	−0.5847	0.5963	−0.4201	−0.0233	0.3818
Preset temp.	0.0719	0.1702	−0.8188	−0.6653	−0.5847	1	−0.4351	0.7993	−0.0718	−0.4696
Room temp. at 16:30	−0.0956	−0.0166	0.4692	0.6274	0.5963	−0.4351	1	−0.3621	−0.1259	0.3588
Room temp. at next 8:00	0.0877	0.2089	−0.6878	−0.5274	−0.4201	0.7993	−0.3621	1	−0.0628	−0.4646
Surface temp.	−0.4842	−0.3783	−0.0844	−0.0758	−0.0233	−0.0718	−0.1259	−0.0628	1	0.3434
Core temp.	−0.0978	0.0685	0.3874	0.4847	0.3818	−0.4696	0.3588	−0.4646	0.3434	1

Inverse matrix	Outside temp.	Outside humidity	Carcass in the day	Carcass after noon	Loading completion	Preset temp.	Room temp. at 16:30	Room temp. at next 8:00	Surface temp.	Core temp.
Outside temp.	1.4772	−0.3198	0.0600	−0.1918	0.1536	0.1425	0.3024	−0.0830	0.6781	−0.1355
Outside humidity	−0.3198	1.6767	−0.1682	0.1337	−0.6778	−0.5110	0.3592	−0.3794	0.6720	−0.6630
Carcass in the day	0.0600	−0.1682	3.4787	0.3741	0.1501	2.7154	−0.5666	0.2799	0.4746	−0.1231
Carcass after noon	−0.1918	0.1337	0.3741	3.3983	−1.4082	1.1741	−0.6959	−0.0110	0.4276	−0.6333
Loading completion	0.1536	−0.6778	0.1501	−1.4082	2.8281	0.9005	−0.7002	−0.1682	−0.3557	0.3244
Preset temp.	0.1425	−0.5110	2.7154	1.1741	0.9005	5.9950	−0.6242	−2.0120	0.4262	0.0425
Room temp. at 16:30	0.3024	0.3592	−0.5666	−0.6959	−0.7002	−0.6242	2.0758	−0.0630	0.5212	−0.4171
Room temp. at next 8:00	−0.0830	−0.3794	0.2799	−0.0110	−0.1682	−2.0120	−0.0630	3.0368	−0.3257	0.5796
Surface temp.	0.6781	0.6720	0.4746	0.4276	−0.3557	0.4262	0.5212	−0.3257	2.0981	−1.0937
Core temp.	−0.1355	−0.6630	−0.1231	−0.6333	0.3244	0.0425	−0.4171	0.5796	−1.0937	2.0775

Partial correlation matrix	Outside temp.	Outside humidity	Carcass in the day	Carcass after noon	Loading completion	Preset temp.	Room temp. at 16:30	Room temp. at next 8:00	Surface temp.	Core temp.
Outside temp.	.	0.2032	−0.0264	0.0856	−0.0752	−0.0479	−0.1727	0.0392	−0.3852	0.0774
Outside humidity	0.2032	.	0.0696	−0.0560	0.3113	0.1612	−0.1925	0.1681	−0.3583	0.3552
Carcass in the day	−0.0264	0.0696	.	−0.1088	−0.0478	−0.5946	0.2109	−0.0861	−0.1757	0.0458
Carcass after noon	0.0856	−0.0560	−0.1088	.	0.4542	−0.2601	0.2620	0.0034	−0.1601	0.2383
Loading completion	−0.0752	0.3113	−0.0478	0.4542	.	−0.2187	0.2890	0.0574	0.1460	−0.1338
Preset temp.	−0.0479	0.1612	−0.5946	−0.2601	−0.2187	.	0.1769	0.4715	−0.1202	−0.0120
Room temp. at 16:30	−0.1727	−0.1925	0.2109	0.2620	0.2890	0.1769	.	0.0251	−0.2497	0.2009
Room temp. at next 8:00	0.0392	0.1681	−0.0861	0.0034	0.0574	0.4715	0.0251	.	0.1291	−0.2308
Surface temp.	−0.3852	−0.3583	−0.1757	−0.1601	0.1460	−0.1202	−0.2497	0.1291	.	0.5239
Core temp.	0.0774	0.3552	0.0458	0.2383	−0.1338	−0.0120	0.2009	−0.2308	0.5239	.

**Table 5:** Data for analysis of seasonal fluctuation (monthly value).

Month	Electric consumption	Operating days	Outside temp. (*1)	Beef carcass	Pork carcass	Carcass volume (*2)
	(kwh)	(day)	(°C)	(head)	(head)	(head)
Apr 2016	84,330	21	23.3	746	17,024	20,008
May 2016	76,720	19	29.0	740	14,802	17,762
Jun 2016	87,300	20	29.5	621	14,677	17,161
Jul 2016	93,850	20	33.5	718	14,367	17,239
Aug 2016	98,770	21	35.3	767	15,417	18,485
Sep 2016	96,240	20	34.4	653	16,142	18,754
Oct 2016	84,140	20	29.4	792	16,337	19,505
Nov 2016	76,960	20	21.5	828	17,912	21,224
Dec 2016	73,540	20	19.6	756	17,038	20,062
Jan 2017	63,200	19	13.1	752	17,339	20,347
Feb 2017	63,770	20	18.3	635	16,473	19,013
Mar 2017	72,060	22	17.5	680	17,438	20,158

(*1) Outside temperatures are calculated as the average of the daily maximum temperature based on the Japan Meteorological Agency.

(*2) Number of beef carcasses is convert to pork for 4:1.

**Table 6:** Correlation matrix for analysis of seasonal fluctuation (No unit in a matrix).

	Electric consumption	Operationg days	Outside temperature	Carcass volume
Electric consumption	1	0.2332	0.9307	−0.5181
Operationg days	0.2332	1	0.0172	0.2021
Outside temperature	0.9307	0.0172	1	−0.7063
Carcass volume	−0.5181	0.2021	−0.7063	1

## 2 Experimental design, materials, and methods

### 2.1 Abbreviations

(In order of appearance)

HACCP: Hazard Analysis and Critical Control Point
MRA: multiple regression analysis
MDA: multivariate data analysis
SEM: structural equation modeling
*r*_*ij* · *rest*_: partial correlation coefficient
*r_ij_*: correlation coefficient matrix *R*
*r^ij^*: invertible matrix $R^{-1}$
NFI: normed fit index
Dev: deviance
RM: reduced model
*i*: number setting partial correlation coefficient to 0
NM: null model
*n*: number of samples
$\hat{\prod (i)}$: estimate of population correlation coefficient of RM
| · |: determinant
*R*: sample correlation coefficient
FM: full model

### 2.2 Prior confirmation

Regarding the risk of representativeness bias, we visited a slaughterhouse when planning this data and confirmed how the FBOs recorded data on-site. We found that the FBOs had established a system in which one person did not record data. The management of the chilling process was rotated among multiple persons. The accuracy was guaranteed because the chief and manager checked the daily records (Figs. 1a–1d and Tables 1a–1d). If the chief noticed any omissions in the entry field when checking the records, he instructed the person in charge to prevent further omissions. Any omission was left blank without supplementing with speculation. The manager confirmed daily that the records were implemented and stored them properly. Transparency was ensured because the records could be viewed by the stakeholders if they applied for viewing.

### 2.3 Collected data

The data were obtained from the management records of chilling operators at the Higashi Mikawa Meat Distribution Center, Toyohashi, Japan for one year from April 2016 to March 2017. This data collection did not require approval, because live animals were not used. The slaughterhouse, Higashi Mikawa Meat Distribution Center Co., Ltd., is a facility approved by the Slaughterhouse Law (1948, Act No. 114) in Japan, and it is inspected and supervised by local civil servants who are veterinarians. It also complies with the "Slaughterhouse Facility Equipment Guidelines" issued by the Ministry of Health, Labor and Welfare (June 23, 1994, Sanitation and Milk No. 97).

The surveillance items to be checked were designed by the operators, which were empirically considered as affectors of the carcass chilling process. The surveillance items were recorded on all operating days in a beef chilling room and a pork chilling room with maximum capacities, with 50 beef carcasses or 240 pork carcasses, respectively. Carcass temperatures were regularly recorded once per week on Monday or Tuesday depending on whether Monday fell on a holiday or a maintenance day.

The surface temperature was measured at the shoulder of beef carcasses and the gluteal region of pork carcasses using an infrared thermometer, SK-8920 (SK SATO, Tokyo, Japan). The core temperature of pork carcass was measured at a depth of 7 cm by a waterproof digital thermometer, SK-250WP (SK SATO) with a standard probe, SWP-01 (SK SATO). Measurement was performed at 8:00 on the day after slaughter and dressing when the beef and pork carcasses were transported from the chilling rooms to the storage rooms or, in the case of some pork carcasses, shipped by refrigerated trucks. The objects of measurement were two carcasses located on each corner of the exit side of each chilling room. The average temperatures of these two carcasses were used as data. The outside air temperature and humidity were measured at 12:00 on the day of slaughter using a digital thermohygrometer, PC-5000TRH-II (SK SATO). This thermohygrometer was placed on the wall of the facility at a height of 120 cm and in a location with appropriate shade. The weather conditions at 12:00 were recorded but were excluded from analyses as they were nominal variables. The room temperature was measured at 16:30 on the day of slaughter and dressing and at 8:00 on the next day using a demand monitor and control equipment, DM-100 (Mitsubishi Electric, Tokyo, Japan).

The number of total slaughtered livestock in a day was recorded, and the maximum number of total slaughtered livestock per day was 65 cattle and 1200 pigs, limited by the capacity for sewage treatment rather than the capacity for chilling and storage. The slaughter and dressing of beef were completed by 13:00 at the latest. In the process of pork slaughter and dressing, approximately 600 heads were slaughtered and dressed by 12:00, and the remaining livestock were slaughtered and dressed by 17:00. The number of carcasses loaded into the chilling room during the day and afternoon, the time of final loading (written as hh + mm/60), and the pre-set chilling temperature were recorded. All items were recorded by visual reading and manually by the chilling operators.

### 2.4 Setting the parameters and the structure

The aforementioned data were input to a spreadsheet in Excel 2010 (Microsoft, WA, USA). Dates were set in rows as data ID, and surveillance items were arranged in the order of the operation process in the column of the matrix as a parameter of causal analysis (Supplementary Datasets 1a and 1b). An array of each row was used as a data unit. When there was a blank space in a data unit due to omission, the data unit was excluded as a missing value. A total of 44 data units for beef and 44 data units for pork were analysed after excluding missing values (Tables 2a and 2b). Eight parameters associated with beef and 10 parameters associated with pork from the surveillance items were selected as continuous variables and were set in three layers as listed in Table 3.

### 2.5 Correlation coefficient matrix and scatterplot matrix

A correlation coefficient matrix (*r_ij_*) among the parameters was calculated using the statistical software JMP 14 (SAS Institute, Inc., NC, USA). The correlation coefficient was distinguished by 0.5 or higher in absolute value to determine the intensity of correlation (Tables 4a and 4b). A scatterplot matrix was drawn using JMP to visualize the data distributions. The 95% probability ellipse, which indicates the two-sigma range, was drawn to determine the variation in the plot area.

### 2.6 Covariance selection and drawing path diagram in GM

Covariance selection was performed by JUSE StatWorks/V5 (The Institute of Japanese Union of Scientists & Engineers, Tokyo, Japan), which is based on Dempster's theory to statistically eliminate spurious correlation [2]. The partial correlation coefficient (*r*_*ij* · *rest*_) among the parameters was calculated from *r_ij_*.

$$r_{ij\cdot rest}=-\frac{r^{ij}}{\sqrt{r^{ii}r^{jj}}}$$

The threshold of *r*_*ij* · *rest*_ was set to 0.1 in absolute value [3]. A pair of parameters with a value of less than 0.1 was disconnected in ascending order (Supplementary Figs. S1a and S1b). NFI represented the goodness of fit and ranged from 0 to 1. When NFI was close to 1, the path graph fit the full model. The threshold of NFI was set to 0.9 [3].

NFI=1−dev{RM(i)}/dev(NM)

$$dev\left\{RM\left(i\right)\right\}=n\log\frac{\left|\hat{\prod^{\left(i\right)}}\right|}{\left|R\right|}$$

This process was performed for every layer in order from the first to the third layer. When NFI remained 0.9 or higher, the threshold of *r*_*ij* · *rest*_ was raised to 0.2, and the process was repeated. After covariance selection, path diagrams were drawn for beef and pork using JUSE StatWorks/V5 in GM. R-code also supports these formulae [4].

The electric consumption, number of operating days, average outside temperature, and number of carcasses in a month were collected to check seasonal fluctuation for the entire slaughterhouse (Table 5). The total number of carcasses reported in Table 5 is given in standardized units of pork carcasses in which one beef carcass are considered equivalent to four pork carcasses. Correlation matrices were constructed from the data in Table 5 (Table 6).

## CRediT authorship contribution statement

**Kumiko Kuzuoka:** Conceptualization, Writing - original draft. **Kohji Kawai:** Project administration, Resources. **Syunpei Yamauchi:** Funding acquisition, Resources. **Ayaka Okada:** Writing - review & editing. **Yasuo Inoshima:** .

## Declaration of Competing Interest

The authors declare that they have no known competing financial interests or personal relationships that have, or could be perceived to have, influenced the work reported in this article.

## Appendix Supplementary materials

Supplementary Dataset S1a. The data processing of input and adjustment of beef. Image, application 1

Supplementary Dataset S1b. The data processing of input and adjustment of pork. Image, application 2

Supplementary Fig. S1a. The data processing of covariance selection of beef. Image, application 3

Supplementary Fig. S1b. The data processing of covariance selection of pork. Image, application 4
