# Supplementary material for: Dataset on causality analysis of chilling process in beef and pork carcasses using graphical modeling
Source: Data Brief. 2020 Jul 25;32:106075. doi: 10.1016/j.dib.2020.106075 (PMC7424210; doi:10.1016/j.dib.2020.106075)
Supplement: Supplementary file 1 — Supplementary Dataset S1a. The data processing of input and adjustment of beef. [file mmc1.pdf]

Supplementary Dataset S1a.
Data procesing of beef.

Date Slaughter	Day of the week Code	Abbreviation	Weather Sunny	Cloudy	Rainny	Cl./ Su.	Ra./ Su.	Snow/ Su.	Outside temp.	Outside humidity	Carcass in the day	Preset temp. of system A	Room temp. at 16:30	Room temp. at 8:00	Setting temp.	Loading completion	Date Slaughter	Day of the week Code	Abbreviation	Surface temp. beef shoulder on the ingate	on the recess	Particular mention
H280404	1	Mo			3				18	86	40	-6.3	1.0	-5.1	-6.0	11:40	H280404	1	Mo	4.5	7.7	
H280411	1	Mo	1						17	34	44	-6.8	1.1	-4.4	-6.5	12:05	H280411	1	Mo	6.0	7.5	
H280419	2	Tu	1						19	33	47	-7.3	1.4	-3.2	-7.0	11:50	H280419	2	Tu	6.7	6.7	
H280426	2	Tu	1						21	51	43	-6.8	-1.7	-6.2	-6.5	12:35	H280426	2	Tu	4.0	3.1	Chilling room 2
H280509	1	Mo			3				20	68	50	-7.3	5.4	-6.0	-7.0	12:10	H280509	1	Mo	5.7	6.8	
H280516	1	Mo		2					21	67	48	-7.3	5.6		-7.0	12:10	H280516	1	Mo	6.1	6.5	
H280523	1	Mo	1						25	44	44	-6.8	4.1	-6.2	-6.5	11:45	H280523	1	Mo	5.7	6.8	
H280530	1	Mo			3				21	80	50	-7.3	4.8	-7.1	-7.0	11:30	H280530	1	Mo	6.9	7.2	
H280606	1	Mo	1						22	58	43	-6.8	4.6	-6.0	-6.5	11:55	H280606	1	Mo	6.1	5.3	
H280613	1	Mo			3				23	86	38	-6.3	3.9		-6.0	11:55	H280613	1	Mo	5.0	4.7	
H280620	1	Mo		2					25	75	39	-6.3	3.8	-6.5	-6.0	11:50	H280620	1	Mo	3.7	3.5	
H280621	2	Tu					2		24	77	40	-6.3	3.4	-6.2	-6.0	11:20	H280621	2	Tu	3.0	3.1	
H280627	1	Mo	1						25	52	49	-7.3	4.3	-7.1	-4.0	12:40	H280627	1	Mo	4.7	6.3	
H280704	1	Mo	1						30	61	49	-7.3	3.5	-6.7	-7.0	12:05	H280704	1	Mo	7.5	8.3	
H280711	1	Mo	1						28	54	47	-7.3	2.7	-6.9	-7.0	11:50	H280711	1	Mo	7.6	7.7	
H280719	2	Tu	1						29	61	51	-7.8	3.5	-8.3	-7.5	12:40	H280719	2	Tu	5.6	5.7	
H280725	1	Mo	1						27	54	48	-7.3	3.7	-7.2	-7.0	11:35	H280725	1	Mo	7.4	5.2	
H280801	1	Mo	1						30	50	51	-7.8	4.3	-6.7	-7.5	12:25	H280801	1	Mo	6.2	4.9	
H280808	1	Mo	1						31	54	16	-4.3	-0.3	-3.5	-4.0	11:55	H280808	1	Mo	4.0	2.4	
H280816	2	Tu				2			28	62	51	-7.8	3.1	-8.1	-7.5	11:40	H280816	2	Tu	6.5	7.3	
H280823	2	Tu	1						29	65	44	-6.8	2.3	-7.5	-6.5	12:40	H280823	2	Tu	6.0	6.8	
H280829	1	Mo	1						31	45	50	-7.3	2.7	-6.4	-7.0	12:10	H280829	1	Mo	5.5	5.1	
H280906	2	Tu	1						27	76	50	-7.3	3.4	-5.1	-7.0	11:20	H280906	2	Tu	6.2	7.0	
H280912	1	Mo		2					27	68	42	-6.8	2.9	-7.4	-6.5	11:45	H280912	1	Mo	4.6	4.1	
H280920	2	Tu			3				24	78	17	-4.5	4.7	-3.6	-4.0	12:40	H280920	2	Tu	3.5	4.2	Pork 160 loaded together. Pork loaded at 16:10
H280926	1	Mo	1						25	77	43	-6.8	2.4	-7.8	-6.5	12:25	H280926	1	Mo	5.7	7.0	
H281003	1	Mo			3				25	84	49	-7.3	4.9	-6.5	-7.0	11:50	H281003	1	Mo	7.6	7.0	
H281011	2	Tu	1						23	50	48	-7.3	6.2	-6.5	-7.0	11:55	H281011	2	Tu	5.3	5.8	
H281017	1	Mo					2		21	81	44	-6.8	6.5	-6.6	-6.5	12:40	H281017	1	Mo	5.6	6.1	
H281024	1	Mo		2					21	39	44	-6.8	5.0	-5.9	-6.5	12:00	H281024	1	Mo	6.3	5.9	
H281031	1	Mo	1						19	50	40	-6.3	4.3	-7.1	-6.0	12:15	H281031	1	Mo	4.3	5.3	
H281101	2	Tu	1						19	49	40	-6.3	4.1	-5.7	-6.0	12:30	H281101	2	Tu	5.3	5.8	
H281107	1	Mo	1						17	43	48	-7.3	6.5	-7.1	-7.0	12:25	H281107	1	Mo	6.5	8.0	
H281114	1	Mo	1						17	63	47	-7.3	2.3	-7.4	-7.0	12:20	H281114	1	Mo	8.2	7.0	
H281121	1	Mo		2					18	63	46	-7.3	3.1	-7.0	-7.0	12:25	H281121	1	Mo	8.2	5.6	Chilling room 2
H281128	1	Mo	1						15	35	50	-7.3	5.6	-6.6	-7.0	12:35	H281128	1	Mo	6.9	7.7	
H281205	1	Mo	1						16	49	50	-7.3	5.8	-8.8	-7.0	11:50	H281205	1	Mo	7.6	9.7	
H281212	1	Mo	1						11	36	50	-7.3	5.0	-7.5	-7.0	12:15	H281212	1	Mo	8.5	9.2	
H281219	1	Mo	1						13	48	27	-5.3	-1.0	-6.2	-5.0	12:00	H281219	1	Mo	4.3	4.4	
H290104	3	We	1						12	46	6	-4.4	4.3	-3.7	-4.0	10:30	H290104	3	We	-	2.9	Pork 188 loaded together. Pork loaded at 16:10
H290110	2	Tu	1						12	46	49	-7.3	5.0	-6.9	-7.0	12:55	H290110	2	Tu	7.5	7.1	
H290116	1	Mo						2	7	48	44	-6.8	5.0	-6.1	-6.5	12:30	H290116	1	Mo	5.6	5.4	
H290123	1	Mo	1						8	43	34	-5.8	4.0	-5.9	-5.5	11:45	H290123	1	Mo	5.2	5.3	
H290130	1	Mo					2		11	72	45	-6.8	5.0	-6.4	-6.5	12:05	H290130	1	Mo	7.6	9.3	
H290206	1	Mo		2					10	51	42	-6.8	5.1	-6.4	-6.5	12:45	H290206	1	Mo	6.1	6.7	
H290217	5	Fr		2					11	46	26	-5.3	1.5		-5.0	12:25	H290217	5	Fr	2.3	2.7	
H290220	1	Mo	1						13	74	46	-7.3	3.9		-7.0	12:35	H290220	1	Mo	8.8	8.7	
H290227	1	Mo	1						12	36	40	-6.3	4.3	-6.1	-6.0	12:00	H290227	1	Mo	5.3	5.2	
H290306	1	Mo			3				12	69	46	-7.3	4.0	-7.3	-7.0	13:00	H290306	1	Mo	6.1	6.0	
H290313	1	Mo		2					12	61	41	-6.8	2.3	-6.9	-6.5	11:40	H290313	1	Mo	7.0	6.3	
H290321	2	Tu			3				13	60	48	-7.3	3.2	-7.7	-7.0	12:05	H290321	2	Tu	7.3	6.2	
H290327	1	Mo	1						13	39	45	-6.8	5.3	-6	-6.5	12:00	H290327	1	Mo	6.0	6.7	

Date Japanese	Date A. D.	=right	=concatenate	Beef_Carcass	=concatenate	Sample ID	Code for day of the week	Code for weather	Outside temp.	Outside humidity	Carcass in the day	Preset temp. of system A	Preset temp. of system B	Room temp. at 16:30	Room tem. at next 8:00	Loading completion	hh	mm	mm/60	hh+mm/60	Completion time of loading	Surface temp. on the ingate	Surface temp. on the recess	Particular mention
H280404	2016	0404	20160404	BC	BC20160404	BC20160404	1	3	18	86	40	-6.3	-6.0	1.0	-5.1	11:40	11	40	0.67	11.67	11.67	4.5	7.7	
H280411	2016	0411	20160411	BC	BC20160411	BC20160411	1	1	17	34	44	-6.8	-6.5	1.1	-4.4	12:05	12	5	0.08	12.08	12.08	6.0	7.5	
H280419	2016	0419	20160419	BC	BC20160419	BC20160419	2	1	19	33	47	-7.3	-7.0	1.4	-3.2	11:50	11	50	0.83	11.83	11.83	6.7	6.7	
H280426	2016	0426	20160426	BC	BC20160426	BC20160426	2	1	21	51	43	-6.8	-6.5	-1.7	-6.2	12:35	12	35	0.58	12.58	12.58	4.0	3.1	Chilling room 2
H280509	2016	0509	20160509	BC	BC20160509	BC20160509	1	3	20	68	50	-7.3	-7.0	5.4	-6.0	12:10	12	10	0.17	12.17	12.17	5.7	6.8	
H280516	2016	0516	20160516	BC	BC20160516	BC20160516	1	2	21	67	48	-7.3	-7.0	5.6		12:10	12	10	0.17	12.17	12.17	6.1	6.5	
H280523	2016	0523	20160523	BC	BC20160523	BC20160523	1	1	25	44	44	-6.8	-6.5	4.1	-6.2	11:45	11	45	0.75	11.75	11.75	5.7	6.8	
H280530	2016	0530	20160530	BC	BC20160530	BC20160530	1	3	21	80	50	-7.3	-7.0	4.8	-7.1	11:30	11	30	0.50	11.50	11.50	6.9	7.2	
H280606	2016	0606	20160606	BC	BC20160606	BC20160606	1	1	22	58	43	-6.8	-6.5	4.6	-6.0	11:55	11	55	0.92	11.92	11.92	6.1	5.3	
H280613	2016	0613	20160613	BC	BC20160613	BC20160613	1	3	23	86	38	-6.3	-6.0	3.9		11:55	11	55	0.92	11.92	11.92	5.0	4.7	
H280620	2016	0620	20160620	BC	BC20160620	BC20160620	1	2	25	75	39	-6.3	-6.0	3.8	-6.5	11:50	11	50	0.83	11.83	11.83	3.7	3.5	
H280621	2016	0621	20160621	BC	BC20160621	BC20160621	2	2	24	77	40	-6.3	-6.0	3.4	-6.2	11:20	11	20	0.33	11.33	11.33	3.0	3.1	
H280627	2016	0627	20160627	BC	BC20160627	BC20160627	1	1	25	52	49	-7.3	-4.0	4.3	-7.1	12:40	12	40	0.67	12.67	12.67	4.7	6.3	
H280704	2016	0704	20160704	BC	BC20160704	BC20160704	1	1	30	61	49	-7.3	-7.0	3.5	-6.7	12:05	12	5	0.08	12.08	12.08	7.5	8.3	
H280711	2016	0711	20160711	BC	BC20160711	BC20160711	1	1	28	54	47	-7.3	-7.0	2.7	-6.9	11:50	11	50	0.83	11.83	11.83	7.6	7.7	
H280719	2016	0719	20160719	BC	BC20160719	BC20160719	2	1	29	61	51	-7.8	-7.5	3.5	-8.3	12:40	12	40	0.67	12.67	12.67	5.6	5.7	
H280725	2016	0725	20160725	BC	BC20160725	BC20160725	1	1	27	54	48	-7.3	-7.0	3.7	-7.2	11:35	11	35	0.58	11.58	11.58	7.4	5.2	
H280801	2016	0801	20160801	BC	BC20160801	BC20160801	1	1	30	50	51	-7.8	-7.5	4.3	-6.7	12:25	12	25	0.42	12.42	12.42	6.2	4.9	
H280808	2016	0808	20160808	BC	BC20160808	BC20160808	1	1	31	54	16	-4.3	-4.0	-0.3	-3.5	11:55	11	55	0.92	11.92	11.92	4.0	2.4	
H280816	2016	0816	20160816	BC	BC20160816	BC20160816	2	2	28	62	51	-7.8	-7.5	3.1	-8.1	11:40	11	40	0.67	11.67	11.67	6.5	7.3	
H280823	2016	0823	20160823	BC	BC20160823	BC20160823	2	1	29	65	44	-6.8	-6.5	2.3	-7.5	12:40	12	40	0.67	12.67	12.67	6.0	6.8	
H280829	2016	0829	20160829	BC	BC20160829	BC20160829	1	1	31	45	50	-7.3	-7.0	2.7	-6.4	12:10	12	10	0.17	12.17	12.17	5.5	5.1	
H280906	2016	0906	20160906	BC	BC20160906	BC20160906	2	1	27	76	50	-7.3	-7.0	3.4	-5.1	11:20	11	20	0.33	11.33	11.33	6.2	7.0	
H280912	2016	0912	20160912	BC	BC20160912	BC20160912	1	2	27	68	42	-6.8	-6.5	2.9	-7.4	11:45	11	45	0.75	11.75	11.75	4.6	4.1	
H280920	2016	0920	20160920	BC	BC20160920	BC20160920	2	3	24	78	17	-4.5	-4.0	4.7	-3.6	12:40	12	40	0.67	12.67	12.67	3.5	4.2	Pork 160 loaded together. Pork loaded at 16:10
H280926	2016	0926	20160926	BC	BC20160926	BC20160926	1	1	25	77	43	-6.8	-6.5	2.4	-7.8	12:25	12	25	0.42	12.42	12.42	5.7	7.0	
H281003	2016	1003	20161003	BC	BC20161003	BC20161003	1	3	25	84	49	-7.3	-7.0	4.9	-6.5	11:50	11	50	0.83	11.83	11.83	7.6	7.0	
H281011	2016	1011	20161011	BC	BC20161011	BC20161011	2	1	23	50	48	-7.3	-7.0	6.2	-6.5	11:55	11	55	0.92	11.92	11.92	5.3	5.8	
H281017	2016	1017	20161017	BC	BC20161017	BC20161017	1	2	21	81	44	-6.8	-6.5	6.5	-6.6	12:40	12	40	0.67	12.67	12.67	5.6	6.1	
H281024	2016	1024	20161024	BC	BC20161024	BC20161024	1	2	21	39	44	-6.8	-6.5	5.0	-5.9	12:00	12	0	0.00	12.00	12.00	6.3	5.9	
H281031	2016	1031	20161031	BC	BC20161031	BC20161031	1	1	19	50	40	-6.3	-6.0	4.3	-7.1	12:15	12	15	0.25	12.25	12.25	4.3	5.3	
H281101	2016	1101	20161101	BC	BC20161101	BC20161101	2	1	19	49	40	-6.3	-6.0	4.1	-5.7	12:30	12	30	0.50	12.50	12.50	5.3	5.8	
H281107	2016	1107	20161107	BC	BC20161107	BC20161107	1	1	17	43	48	-7.3	-7.0	6.5	-7.1	12:25	12	25	0.42	12.42	12.42	6.5	8.0	
H281114	2016	1114	20161114	BC	BC20161114	BC20161114	1	1	17	63	47	-7.3	-7.0	2.3	-7.4	12:20	12	20	0.33	12.33	12.33	8.2	7.0	
H281121	2016	1121	20161121	BC	BC20161121	BC20161121	1	2	18	63	46	-7.3	-7.0	3.1	-7.0	12:25	12	25	0.42	12.42	12.42	8.2	5.6	Chilling room 2
H281128	2016	1128	20161128	BC	BC20161128	BC20161128	1	1	15	35	50	-7.3	-7.0	5.6	-6.6	12:35	12	35	0.58	12.58	12.58	6.9	7.7	
H281205	2016	1205	20161205	BC	BC20161205	BC20161205	1	1	16	49	50	-7.3	-7.0	5.8	-8.8	11:50	11	50	0.83	11.83	11.83	7.6	9.7	
H281212	2016	1212	20161212	BC	BC20161212	BC20161212	1	1	11	36	50	-7.3	-7.0	5.0	-7.5	12:15	12	15	0.25	12.25	12.25	8.5	9.2	
H281219	2016	1219	20161219	BC	BC20161219	BC20161219	1	1	13	48	27	-5.3	-5.0	-1.0	-6.2	12:00	12	0	0.00	12.00	12.00	4.3	4.4	
H290104	2016	0104	20160104	BC	BC20160104	BC20160104	3	1	12	46	6	-4.4	-4.0	4.3	-3.7	10:30	10	30	0.50	10.50	10.50	-	2.9	Pork 188 loaded together. Pork loaded at 16:10
H290110	2016	0110	20160110	BC	BC20160110	BC20160110	2	1	12	46	49	-7.3	-7.0	5.0	-6.9	12:55	12	55	0.92	12.92	12.92	7.5	7.1	
H290116	2016	0116	20160116	BC	BC20160116	BC20160116	1	2	7	48	44	-6.8	-6.5	5.0	-6.1	12:30	12	30	0.50	12.50	12.50	5.6	5.4	
H290123	2016	0123	20160123	BC	BC20160123	BC20160123	1	1	8	43	34	-5.8	-5.5	4.0	-5.9	11:45	11	45	0.75	11.75	11.75	5.2	5.3	
H290130	2016	0130	20160130	BC	BC20160130	BC20160130	1	2	11	72	45	-6.8	-6.5	5.0	-6.4	12:05	12	5	0.08	12.08	12.08	7.6	9.3	
H290206	2016	0206	20160206	BC	BC20160206	BC20160206	1	2	10	51	42	-6.8	-6.5	5.1	-6.4	12:45	12	45	0.75	12.75	12.75	6.1	6.7	
H290217	2016	0217	20160217	BC	BC20160217	BC20160217	5	2	11	46	26	-5.3	-5.0	1.5		12:25	12	25	0.42	12.42	12.42	2.3	2.7	
H290220	2016	0220	20160220	BC	BC20160220	BC20160220	1	1	13	74	46	-7.3	-7.0	3.9		12:35	12	35	0.58	12.58	12.58	8.8	8.7	
H290227	2016	0227	20160227	BC	BC20160227	BC20160227	1	1	12	36	40	-6.3	-6.0	4.3	-6.1	12:00	12	0	0.00	12.00	12.00	5.3	5.2	
H290306	2016	0306	20160306	BC	BC20160306	BC20160306	1	3	12	69	46	-7.3	-7.0	4.0	-7.3	13:00	13	0	0.00	13.00	13.00	6.1	6.0	
H290313	2016	0313	20160313	BC	BC20160313	BC20160313	1	2	12	61	41	-6.8	-6.5	2.3	-6.9	11:40	11	40	0.67	11.67	11.67	7.0	6.3	
H290321	2016	0321	20160321	BC	BC20160321	BC20160321	2	3	13	60	48	-7.3	-7.0	3.2	-7.7	12:05	12	5	0.08	12.08	12.08	7.3	6.2	
H290327	2016	0327	20160327	BC	BC20160327	BC20160327	1	1	13	39	45	-6.8	-6.5	5.3	-6	12:00	12	0	0.00	12.00	12.00	6.0	6.7	

Sample ID	Outside temp.	Outside humidity	Carcass in the day	Loading completion	Preset temp. of system A	Preset temp. of system B	Room temp. at 16:30	Room temp. at next 8:00	Surface temp. on the ingate	Surface temp. on the recess
BC20160404	18	86	40	11.67	-6.3	-6.0	1.0	-5.1	4.5	7.7
BC20160411	17	34	44	12.08	-6.8	-6.5	1.1	-4.4	6.0	7.5
BC20160419	19	33	47	11.83	-7.3	-7.0	1.4	-3.2	6.7	6.7
BC20160426	21	51	43	12.58	-6.8	-6.5	-1.7	-6.2	4.0	3.1
BC20160509	20	68	50	12.17	-7.3	-7.0	5.4	-6.0	5.7	6.8
BC20160516	21	67	48	12.17	-7.3	-7.0	5.6		6.1	6.5
BC20160523	25	44	44	11.75	-6.8	-6.5	4.1	-6.2	5.7	6.8
BC20160530	21	80	50	11.50	-7.3	-7.0	4.8	-7.1	6.9	7.2
BC20160606	22	58	43	11.92	-6.8	-6.5	4.6	-6.0	6.1	5.3
BC20160613	23	86	38	11.92	-6.3	-6.0	3.9		5.0	4.7
BC20160620	25	75	39	11.83	-6.3	-6.0	3.8	-6.5	3.7	3.5
BC20160621	24	77	40	11.33	-6.3	-6.0	3.4	-6.2	3.0	3.1
BC20160627	25	52	49	12.67	-7.3	-4.0	4.3	-7.1	4.7	6.3
BC20160704	30	61	49	12.08	-7.3	-7.0	3.5	-6.7	7.5	8.3
BC20160711	28	54	47	11.83	-7.3	-7.0	2.7	-6.9	7.6	7.7
BC20160719	29	61	51	12.67	-7.8	-7.5	3.5	-8.3	5.6	5.7
BC20160725	27	54	48	11.58	-7.3	-7.0	3.7	-7.2	7.4	5.2
BC20160801	30	50	51	12.42	-7.8	-7.5	4.3	-6.7	6.2	4.9
BC20160808	31	54	16	11.92	-4.3	-4.0	-0.3	-3.5	4.0	2.4
BC20160816	28	62	51	11.67	-7.8	-7.5	3.1	-8.1	6.5	7.3
BC20160823	29	65	44	12.67	-6.8	-6.5	2.3	-7.5	6.0	6.8
BC20160829	31	45	50	12.17	-7.3	-7.0	2.7	-6.4	5.5	5.1
BC20160906	27	76	50	11.33	-7.3	-7.0	3.4	-5.1	6.2	7.0
BC20160912	27	68	42	11.75	-6.8	-6.5	2.9	-7.4	4.6	4.1
BC20160920	24	78	17	12.67	-4.5	-4.0	4.7	-3.6	3.5	4.2
BC20160926	25	77	43	12.42	-6.8	-6.5	2.4	-7.8	5.7	7.0
BC20161003	25	84	49	11.83	-7.3	-7.0	4.9	-6.5	7.6	7.0
BC20161011	23	50	48	11.92	-7.3	-7.0	6.2	-6.5	5.3	5.8
BC20161017	21	81	44	12.67	-6.8	-6.5	6.5	-6.6	5.6	6.1
BC20161024	21	39	44	12.00	-6.8	-6.5	5.0	-5.9	6.3	5.9
BC20161031	19	50	40	12.25	-6.3	-6.0	4.3	-7.1	4.3	5.3
BC20161101	19	49	40	12.50	-6.3	-6.0	4.1	-5.7	5.3	5.8
BC20161107	17	43	48	12.42	-7.3	-7.0	6.5	-7.1	6.5	8.0
BC20161114	17	63	47	12.33	-7.3	-7.0	2.3	-7.4	8.2	7.0
BC20161121	18	63	46	12.42	-7.3	-7.0	3.1	-7.0	8.2	5.6
BC20161128	15	35	50	12.58	-7.3	-7.0	5.6	-6.6	6.9	7.7
BC20161205	16	49	50	11.83	-7.3	-7.0	5.8	-8.8	7.6	9.7
BC20161212	11	36	50	12.25	-7.3	-7.0	5.0	-7.5	8.5	9.2
BC20161219	13	48	27	12.00	-5.3	-5.0	-1.0	-6.2	4.3	4.4
BC20160104	12	46	6	10.50	-4.4	-4.0	4.3	-3.7		2.9
BC20160110	12	46	49	12.92	-7.3	-7.0	5.0	-6.9	7.5	7.1
BC20160116	7	48	44	12.50	-6.8	-6.5	5.0	-6.1	5.6	5.4
BC20160123	8	43	34	11.75	-5.8	-5.5	4.0	-5.9	5.2	5.3
BC20160130	11	72	45	12.08	-6.8	-6.5	5.0	-6.4	7.6	9.3
BC20160206	10	51	42	12.75	-6.8	-6.5	5.1	-6.4	6.1	6.7
BC20160217	11	46	26	12.42	-5.3	-5.0	1.5		2.3	2.7
BC20160220	13	74	46	12.58	-7.3	-7.0	3.9		8.8	8.7
BC20160227	12	36	40	12.00	-6.3	-6.0	4.3	-6.1	5.3	5.2
BC20160306	12	69	46	13.00	-7.3	-7.0	4.0	-7.3	6.1	6.0
BC20160313	12	61	41	11.67	-6.8	-6.5	2.3	-6.9	7.0	6.3
BC20160321	13	60	48	12.08	-7.3	-7.0	3.2	-7.7	7.3	6.2
BC20160327	13	39	45	12.00	-6.8	-6.5	5.3	-6	6.0	6.7

Sample ID	Outside temp.	Outside humidity	Carcass in the day	Loading completion	Preset temp. of system A	Preset temp. of system B	Room temp. at 16:30	Room temp. at next 8:00	Surface temp. on the ingate	Surface temp. on the recess
BC20160404	18	86	40	11.67	-6.3	-6.0	1.0	-5.1	4.5	7.7
BC20160411	17	34	44	12.08	-6.8	-6.5	1.1	-4.4	6.0	7.5
BC20160419	19	33	47	11.83	-7.3	-7.0	1.4	-3.2	6.7	6.7
BC20160509	20	68	50	12.17	-7.3	-7.0	5.4	-6.0	5.7	6.8
BC20160523	25	44	44	11.75	-6.8	-6.5	4.1	-6.2	5.7	6.8
BC20160530	21	80	50	11.50	-7.3	-7.0	4.8	-7.1	6.9	7.2
BC20160606	22	58	43	11.92	-6.8	-6.5	4.6	-6.0	6.1	5.3
BC20160620	25	75	39	11.83	-6.3	-6.0	3.8	-6.5	3.7	3.5
BC20160621	24	77	40	11.33	-6.3	-6.0	3.4	-6.2	3.0	3.1
BC20160627	25	52	49	12.67	-7.3	-4.0	4.3	-7.1	4.7	6.3
BC20160704	30	61	49	12.08	-7.3	-7.0	3.5	-6.7	7.5	8.3
BC20160711	28	54	47	11.83	-7.3	-7.0	2.7	-6.9	7.6	7.7
BC20160719	29	61	51	12.67	-7.8	-7.5	3.5	-8.3	5.6	5.7
BC20160725	27	54	48	11.58	-7.3	-7.0	3.7	-7.2	7.4	5.2
BC20160801	30	50	51	12.42	-7.8	-7.5	4.3	-6.7	6.2	4.9
BC20160808	31	54	16	11.92	-4.3	-4.0	-0.3	-3.5	4.0	2.4
BC20160816	28	62	51	11.67	-7.8	-7.5	3.1	-8.1	6.5	7.3
BC20160823	29	65	44	12.67	-6.8	-6.5	2.3	-7.5	6.0	6.8
BC20160829	31	45	50	12.17	-7.3	-7.0	2.7	-6.4	5.5	5.1
BC20160906	27	76	50	11.33	-7.3	-7.0	3.4	-5.1	6.2	7.0
BC20160912	27	68	42	11.75	-6.8	-6.5	2.9	-7.4	4.6	4.1
BC20160926	25	77	43	12.42	-6.8	-6.5	2.4	-7.8	5.7	7.0
BC20161003	25	84	49	11.83	-7.3	-7.0	4.9	-6.5	7.6	7.0
BC20161011	23	50	48	11.92	-7.3	-7.0	6.2	-6.5	5.3	5.8
BC20161017	21	81	44	12.67	-6.8	-6.5	6.5	-6.6	5.6	6.1
BC20161024	21	39	44	12.00	-6.8	-6.5	5.0	-5.9	6.3	5.9
BC20161031	19	50	40	12.25	-6.3	-6.0	4.3	-7.1	4.3	5.3
BC20161101	19	49	40	12.50	-6.3	-6.0	4.1	-5.7	5.3	5.8
BC20161107	17	43	48	12.42	-7.3	-7.0	6.5	-7.1	6.5	8.0
BC20161114	17	63	47	12.33	-7.3	-7.0	2.3	-7.4	8.2	7.0
BC20161128	15	35	50	12.58	-7.3	-7.0	5.6	-6.6	6.9	7.7
BC20161205	16	49	50	11.83	-7.3	-7.0	5.8	-8.8	7.6	9.7
BC20161212	11	36	50	12.25	-7.3	-7.0	5.0	-7.5	8.5	9.2
BC20161219	13	48	27	12.00	-5.3	-5.0	-1.0	-6.2	4.3	4.4
BC20160110	12	46	49	12.92	-7.3	-7.0	5.0	-6.9	7.5	7.1
BC20160116	7	48	44	12.50	-6.8	-6.5	5.0	-6.1	5.6	5.4
BC20160123	8	43	34	11.75	-5.8	-5.5	4.0	-5.9	5.2	5.3
BC20160130	11	72	45	12.08	-6.8	-6.5	5.0	-6.4	7.6	9.3
BC20160206	10	51	42	12.75	-6.8	-6.5	5.1	-6.4	6.1	6.7
BC20160227	12	36	40	12.00	-6.3	-6.0	4.3	-6.1	5.3	5.2
BC20160306	12	69	46	13.00	-7.3	-7.0	4.0	-7.3	6.1	6.0
BC20160313	12	61	41	11.67	-6.8	-6.5	2.3	-6.9	7.0	6.3
BC20160321	13	60	48	12.08	-7.3	-7.0	3.2	-7.7	7.3	6.2
BC20160327	13	39	45	12.00	-6.8	-6.5	5.3	-6	6.0	6.7

Sample ID	Outside temp.	Outside humidity	Carcass in the day	Loading completion	Preset temp. of system A	Preset temp. of system B	Preset temp.	Room temp. at 16:30	Room temp. at next 8:00	Surface temp. on the ingate	Surface temp. on the recess	Surface temp.
BC20160404	18	86	40	11.67	-6.3	-6.0	-6.2	1.0	-5.1	4.5	7.7	6.1
BC20160411	17	34	44	12.08	-6.8	-6.5	-6.7	1.1	-4.4	6.0	7.5	6.8
BC20160419	19	33	47	11.83	-7.3	-7.0	-7.2	1.4	-3.2	6.7	6.7	6.7
BC20160509	20	68	50	12.17	-7.3	-7.0	-7.2	5.4	-6.0	5.7	6.8	6.3
BC20160523	25	44	44	11.75	-6.8	-6.5	-6.7	4.1	-6.2	5.7	6.8	6.3
BC20160530	21	80	50	11.50	-7.3	-7.0	-7.2	4.8	-7.1	6.9	7.2	7.1
BC20160606	22	58	43	11.92	-6.8	-6.5	-6.7	4.6	-6.0	6.1	5.3	5.7
BC20160620	25	75	39	11.83	-6.3	-6.0	-6.2	3.8	-6.5	3.7	3.5	3.6
BC20160621	24	77	40	11.33	-6.3	-6.0	-6.2	3.4	-6.2	3.0	3.1	3.1
BC20160627	25	52	49	12.67	-7.3	-4.0	-5.7	4.3	-7.1	4.7	6.3	5.5
BC20160704	30	61	49	12.08	-7.3	-7.0	-7.2	3.5	-6.7	7.5	8.3	7.9
BC20160711	28	54	47	11.83	-7.3	-7.0	-7.2	2.7	-6.9	7.6	7.7	7.7
BC20160719	29	61	51	12.67	-7.8	-7.5	-7.7	3.5	-8.3	5.6	5.7	5.7
BC20160725	27	54	48	11.58	-7.3	-7.0	-7.2	3.7	-7.2	7.4	5.2	6.3
BC20160801	30	50	51	12.42	-7.8	-7.5	-7.7	4.3	-6.7	6.2	4.9	5.6
BC20160808	31	54	16	11.92	-4.3	-4.0	-4.2	-0.3	-3.5	4.0	2.4	3.2
BC20160816	28	62	51	11.67	-7.8	-7.5	-7.7	3.1	-8.1	6.5	7.3	6.9
BC20160823	29	65	44	12.67	-6.8	-6.5	-6.7	2.3	-7.5	6.0	6.8	6.4
BC20160829	31	45	50	12.17	-7.3	-7.0	-7.2	2.7	-6.4	5.5	5.1	5.3
BC20160906	27	76	50	11.33	-7.3	-7.0	-7.2	3.4	-5.1	6.2	7.0	6.6
BC20160912	27	68	42	11.75	-6.8	-6.5	-6.7	2.9	-7.4	4.6	4.1	4.4
BC20160926	25	77	43	12.42	-6.8	-6.5	-6.7	2.4	-7.8	5.7	7.0	6.4
BC20161003	25	84	49	11.83	-7.3	-7.0	-7.2	4.9	-6.5	7.6	7.0	7.3
BC20161011	23	50	48	11.92	-7.3	-7.0	-7.2	6.2	-6.5	5.3	5.8	5.6
BC20161017	21	81	44	12.67	-6.8	-6.5	-6.7	6.5	-6.6	5.6	6.1	5.9
BC20161024	21	39	44	12.00	-6.8	-6.5	-6.7	5.0	-5.9	6.3	5.9	6.1
BC20161031	19	50	40	12.25	-6.3	-6.0	-6.2	4.3	-7.1	4.3	5.3	4.8
BC20161101	19	49	40	12.50	-6.3	-6.0	-6.2	4.1	-5.7	5.3	5.8	5.6
BC20161107	17	43	48	12.42	-7.3	-7.0	-7.2	6.5	-7.1	6.5	8.0	7.3
BC20161114	17	63	47	12.33	-7.3	-7.0	-7.2	2.3	-7.4	8.2	7.0	7.6
BC20161128	15	35	50	12.58	-7.3	-7.0	-7.2	5.6	-6.6	6.9	7.7	7.3
BC20161205	16	49	50	11.83	-7.3	-7.0	-7.2	5.8	-8.8	7.6	9.7	8.7
BC20161212	11	36	50	12.25	-7.3	-7.0	-7.2	5.0	-7.5	8.5	9.2	8.9
BC20161219	13	48	27	12.00	-5.3	-5.0	-5.2	-1.0	-6.2	4.3	4.4	4.4
BC20160110	12	46	49	12.92	-7.3	-7.0	-7.2	5.0	-6.9	7.5	7.1	7.3
BC20160116	7	48	44	12.50	-6.8	-6.5	-6.7	5.0	-6.1	5.6	5.4	5.5
BC20160123	8	43	34	11.75	-5.8	-5.5	-5.7	4.0	-5.9	5.2	5.3	5.3
BC20160130	11	72	45	12.08	-6.8	-6.5	-6.7	5.0	-6.4	7.6	9.3	8.5
BC20160206	10	51	42	12.75	-6.8	-6.5	-6.7	5.1	-6.4	6.1	6.7	6.4
BC20160227	12	36	40	12.00	-6.3	-6.0	-6.2	4.3	-6.1	5.3	5.2	5.3
BC20160306	12	69	46	13.00	-7.3	-7.0	-7.2	4.0	-7.3	6.1	6.0	6.1
BC20160313	12	61	41	11.67	-6.8	-6.5	-6.7	2.3	-6.9	7.0	6.3	6.7
BC20160321	13	60	48	12.08	-7.3	-7.0	-7.2	3.2	-7.7	7.3	6.2	6.8
BC20160327	13	39	45	12.00	-6.8	-6.5	-6.7	5.3	-6	6.0	6.7	6.4

Sample ID	Outside temp.	Outside humidity	Carcass in the day	Loading completion	Preset temp.	Room temp. at 16:30	Room temp. at next 8:00	Surface temp.
BC20160404	18	86	40	11.67	-6.2	1.0	-5.1	6.1
BC20160411	17	34	44	12.08	-6.7	1.1	-4.4	6.8
BC20160419	19	33	47	11.83	-7.2	1.4	-3.2	6.7
BC20160509	20	68	50	12.17	-7.2	5.4	-6.0	6.3
BC20160523	25	44	44	11.75	-6.7	4.1	-6.2	6.3
BC20160530	21	80	50	11.50	-7.2	4.8	-7.1	7.1
BC20160606	22	58	43	11.92	-6.7	4.6	-6.0	5.7
BC20160620	25	75	39	11.83	-6.2	3.8	-6.5	3.6
BC20160621	24	77	40	11.33	-6.2	3.4	-6.2	3.1
BC20160627	25	52	49	12.67	-5.7	4.3	-7.1	5.5
BC20160704	30	61	49	12.08	-7.2	3.5	-6.7	7.9
BC20160711	28	54	47	11.83	-7.2	2.7	-6.9	7.7
BC20160719	29	61	51	12.67	-7.7	3.5	-8.3	5.7
BC20160725	27	54	48	11.58	-7.2	3.7	-7.2	6.3
BC20160801	30	50	51	12.42	-7.7	4.3	-6.7	5.6
BC20160808	31	54	16	11.92	-4.2	-0.3	-3.5	3.2
BC20160816	28	62	51	11.67	-7.7	3.1	-8.1	6.9
BC20160823	29	65	44	12.67	-6.7	2.3	-7.5	6.4
BC20160829	31	45	50	12.17	-7.2	2.7	-6.4	5.3
BC20160906	27	76	50	11.33	-7.2	3.4	-5.1	6.6
BC20160912	27	68	42	11.75	-6.7	2.9	-7.4	4.4
BC20160926	25	77	43	12.42	-6.7	2.4	-7.8	6.4
BC20161003	25	84	49	11.83	-7.2	4.9	-6.5	7.3
BC20161011	23	50	48	11.92	-7.2	6.2	-6.5	5.6
BC20161017	21	81	44	12.67	-6.7	6.5	-6.6	5.9
BC20161024	21	39	44	12.00	-6.7	5.0	-5.9	6.1
BC20161031	19	50	40	12.25	-6.2	4.3	-7.1	4.8
BC20161101	19	49	40	12.50	-6.2	4.1	-5.7	5.6
BC20161107	17	43	48	12.42	-7.2	6.5	-7.1	7.3
BC20161114	17	63	47	12.33	-7.2	2.3	-7.4	7.6
BC20161128	15	35	50	12.58	-7.2	5.6	-6.6	7.3
BC20161205	16	49	50	11.83	-7.2	5.8	-8.8	8.7
BC20161212	11	36	50	12.25	-7.2	5.0	-7.5	8.9
BC20161219	13	48	27	12.00	-5.2	-1.0	-6.2	4.4
BC20160110	12	46	49	12.92	-7.2	5.0	-6.9	7.3
BC20160116	7	48	44	12.50	-6.7	5.0	-6.1	5.5
BC20160123	8	43	34	11.75	-5.7	4.0	-5.9	5.3
BC20160130	11	72	45	12.08	-6.7	5.0	-6.4	8.5
BC20160206	10	51	42	12.75	-6.7	5.1	-6.4	6.4
BC20160227	12	36	40	12.00	-6.2	4.3	-6.1	5.3
BC20160306	12	69	46	13.00	-7.2	4.0	-7.3	6.1
BC20160313	12	61	41	11.67	-6.7	2.3	-6.9	6.7
BC20160321	13	60	48	12.08	-7.2	3.2	-7.7	6.8
BC20160327	13	39	45	12.00	-6.7	5.3	-6	6.4
