# Supplementary material for: Dataset on causality analysis of chilling process in beef and pork carcasses using graphical modeling
Source: Data Brief. 2020 Jul 25;32:106075. doi: 10.1016/j.dib.2020.106075 (PMC7424210; doi:10.1016/j.dib.2020.106075)
Supplement: Supplementary file 2 — Supplementary Dataset S1b. The data processing of input and adjustment of pork. [file mmc2.pdf]

Supplementary Dataset S1b.
Data processing of pork.

Date	Day of the week					Weather				Outside	Outside	Carcass	Preset temp.	Room temp.	Room temp.	Setting	Loading	Carcass	Date	Day of the week		Surface temp. pork glute.		Core temp. pork glute.	
Slaughter	Code	Abbreviation	Sunny	Cloudy	Rainny	Cl./ Su.	Cl./ Ra.	Ra. /Su.	Snow/ su.	temp.	humidity	in the day	of system A	at 16:30	at 8:00	temp.	completion	after noon	Slaughter	Code	Abbreviation	on the ingate	on the recess	on the ingate	on the recess
	Prohibition setting																			Prohibition setting					
H280404	1	Mo			3					18	86	110	-2.9	-3.8	-2.1	-2.5	12:35	0	H280405	2	Tu	-2.5	-3.0	0.3	-0.1
H280411	1	Mo	1							17	34	115	-3.0	-2.9	-2.2	-2.5	14:30	18	H280412	2	Tu	-0.7	-0.1	-0.2	0.1
H280421	4	Th			3					18	81	169	-3.4	2.6	-3.7	-3.0	15:50	95	H280422	5	Fr	1.6	0.8	1.7	0.2
H280426	2	Tu	1							21	51	242	-4.4	0.4	-4	-4.0	15:35	20	H280427	3	We	1.0	1.5	0.4	0.5
H280505	4	Th	1							23	29	262	-4.8	3.1	-4.8	-4.5	16:00	88	H280506	5	Fr	-2.0	-2.9	0.6	-0.2
H280510	2	Tu		2						20	77	229	-3.4	1.7	-3.7	-3.0	15:30	30	H280511	3	We	-4.8	-5.0	0.5	-0.6
H280516	1	Mo		2						21	67	210	-3.2	3.0		-3.0	12:55	0	H280517	2	Tu	-3.0	-4.0	0.6	0.1
H280523	1	Mo	1							25	44	119	-2.9	-2.8	-3.5	-2.5	12:25	0	H280524	2	Tu	-6.8	-7.3	-0.1	-0.9
H280530	1	Mo			3					21	80	113	-2.9	-2.2	-3	-2.5	12:35	0	H280531	2	Tu	-4.5	-4.8	-0.1	-0.4
H280607	2	Tu					2			22	67	165	-3.1	0.9	-2.9	-2.5	14:30	31	H280608	3	We	-4.9	-4.2	0.2	0.3
H280613	1	Mo			3					23	86	127	-3.0	-2.1		-2.5	14:15	24	H280614	2	Tu	-4.6	-5.3	0.8	0.0
H280620	1	Mo		2						25	75	214	-3.3	1.3	-3.4	-3.0	13:50	33	H280621	2	Tu	-4.7	-5.7	0.4	0.1
H280708	5	Fr		2						27	71	201	-3.5	3.9	-2	-3.0	16:00	95	H280709	6	Sa	-6.2	-6.5	0.2	-0.2
H280711	1	Mo	1							28	54	211	-3.2	-0.8	-3.4	-3.0	13:00	0	H280712	2	Tu	-3.9	-4.7	0.0	-0.3
H280719	2	Tu	1							29	61	217	-3.5	4.7	-3.6	-3.0	15:40	90	H280720	3	We	-4.8	-5.4	1.1	-0.3
H280726	2	Tu			3					25	76	186	-3.2	1.5	-3.1	-3.0	14:40	28	H280727	3	We	-4.3	-5.2	-0.6	-0.8
H280801	1	Mo	1							30	50	210	-3.2	0.3	-2.8	-3.0	12:30	0	H280802	2	Tu	-5.8	-5.5	-0.5	-0.6
H280808	1	Mo	1							31	54	109	-3.0	2.3	-2.9	-2.5	14:00	18	H280809	2	Tu	-3.0	-4.5	1.3	-0.4
H280816	2	Tu				2				28	62	220	-3.5	0.8	-3.7	-3.0	15:10	76	H280817	3	We	-3.5	-3.8	0.8	-0.6
H280830	2	Tu	1							27	46	163	-3.1	2.3	-3.0	-2.5	14:30	36	H280831	3	We	-5.3	-4.8	-0.5	-0.8
H280905	1	Mo					2			27	86	109	-2.5	1.7	-2.7	-2.0	14:20	19	H280906	2	Tu	-4.7	-5.4	0.3	-0.6
H280913	2	Tu		2						26	86	170	-3.3	4.0	-3.4	-3.0	15:10	57	H280914	3	We	-4.4	-5.4	-0.1	-0.9
H280920	2	Tu			3					24	78	228	-3.9	-3.3	-4	-3.5	16:10	63	H280921	3	We	-6.0	-6.1	0.2	-0.7
H280926	1	Mo	1							25	77	124	-3.0	3.2	-2.8	-2.5	14:30	28	H280927	2	Tu	-5.1	-5.0	-0.2	-0.6
H281011	2	Tu	1							23	50	234	-4.1	5.8	-3.6	-3.5	15:20	130	H281012	3	We	-5.4	-5.1	-0.3	0.7
H281017	1	Mo						2		21	81	137	-3.0	1.8	-2.5	-2.5	15:55	38	H281018	2	Tu	-5.7	-5.3	0.1	-0.4
H281025	2	Tu			3					19	63	200	-3.5	5.0	-3.6	-3.0	15:05	103	H281026	3	We	-3.5	-5.0	-0.1	0.2
H281102	3	We	1							17	43	258	-4.2	2.1	-4.1	-4.0	15:55	113	H281103	4	Th	-1.8	-2.5	0.1	-0.1
H281107	1	Mo	1							17	43	115	-3	2.4	-3	-2.5	14:45	27	H281108	2	Tu	-2.2	-2.4	0.3	-0.2
H281114	1	Mo	1							17	63	155	-3.1	-0.3	-2.7	-2.5	14:25	17	H281115	2	Tu	-2.5	-2.8	-0.3	-0.6
H281121	1	Mo		2						18	63	233	-4.5	5.3	-4	-4.0	15:15	56	H281122	2	Tu	-1.9	-3.5	0.1	0.3
H281128	1	Mo	1							15	35	132	-2.9	-1.4	-3.6	-2.5	12:25	0	H281129	2	Tu	-0.1	-0.5	-0.1	-0.6
H281206	2	Tu	1							16	40	165	-3.2	2.6	-3.6	-3.0	14:55	62	H281207	3	We	-2.2	-1.9	0.8	-0.8
H281213	2	Tu			3					11	66	229	-4.0	3.4	-3.3	-3.5	15:50	103	H281214	3	We	-4.7	-5.3	0.5	-0.6
H281219	1	Mo	1							13	48	154	-3.1	3	-2.2	-2.5	15:10	43	H281220	2	Tu	-0.5	-1.0	0.1	-0.4
H290104	3	WE	1							12	46	199	-3.4	4.8	-4.1	-3.0	16:10	68	H290105	4	Th	-3.4	-5.1	0.4	-0.1
H290111	3	We		2						12	41	228	-4.0	5.3	-3.9	-3.5	16:00	84	H290112	4	Th	-3.1	-3.9	0.6	-0.5
H290116	1	Mo							2	7	48	154	-3.1	4.3	-3.1	-2.5	15:05	38	H290117	2	Tu	-2.0	-2.6	0.4	-0.5
H290123	1	Mo	1							8	43	127	-2.9	1.4	-2.6	-2.5	14:30	14	H290124	2	Tu	-3.0	-3.6	-0.5	-0.8
H290131	2	Tu	1							10	34	253	-4.0			-3.5	16:05	92	H290201	3	We	-1.6	-3.1	1.3	-0.6
H290206	1	Mo				2				10	51	236	-3.3	3.6	-3.3	-3.0	12:40	0	H290207	2	Tu	-2.8	-3.5	0.8	-0.4
H290213	1	Mo	1							9	40	144	-3.0	-2.3	-2.8	-2.5	12:35	0	H290214	2	Tu	-1.7	-2.4	-0.2	-0.6
H290227	1	Mo	1							12	36	126	-2.9	-2.2	-2.8	-2.5	12:40	0	H290228	2	Tu	-0.1	-0.6	0.3	-0.3
H290306	1	Mo			3					12	69	234	-3.3	2.5	-3.4	-3.0	15:50	0	H290307	2	Tu	-3.2	-3.8	0.8	0.0
H290314	2	Tu	1							14	37	196	-4.5	3.1	-4.4	-4.0	16:00	52	H290315	3	We	-4.0	-4.9	0.4	-0.8
H290321	2	Tu			3					13	60	261	-5.0	4.9	-5.4	-4.5	16:20	154	H290322	3	We	-2.3	-3.5	1.3	1.2
H290327	1	Mo	1							13	39	214	-3.2	2.7	-3.5	-3.0	12:40	0	H290328	2	Tu	-3.2	-3.9	0.2	-0.4

Date Japanese	Date A. D.	=right	=concatenate	Pork_Carcass	=concatenate	Sample ID	Code for day of the week	Code for weather	Outside temp.	Outside humidity	Carcass in the day	Preset temp. of system A	Preset temp. of system B	Room temp. at 16:30	Room tem. at next 8:00	Loaded completion	hh	mm	mm/60	hh+mm/60	Loading completion	Carcass after noon	Surface temp. on the ingate	Surface temp. on the recess	Core temp. on the ingate	Core temp. on the recess
H280404	2016	0404	20160404	PC	PC20160404	PC20160404	1	3	18	86	110	-2.9	-2.5	-3.8	-2.1	12:35	12	35	0.58	12.58	12.58	0	-2.5	-3.0	0.3	-0.1
H280411	2016	0411	20160411	PC	PC20160411	PC20160411	1	1	17	34	115	-3.0	-2.5	-2.9	-2.2	14:30	14	30	0.50	14.50	14.50	18	-0.7	-0.1	-0.2	0.1
H280421	2016	0421	20160421	PC	PC20160421	PC20160421	4	3	18	81	169	-3.4	-3.0	2.6	-3.7	15:50	15	50	0.83	15.83	15.83	95	1.6	0.8	1.7	0.2
H280426	2016	0426	20160426	PC	PC20160426	PC20160426	2	1	21	51	242	-4.4	-4.0	0.4	-4	15:35	15	35	0.58	15.58	15.58	20	1.0	1.5	0.4	0.5
H280505	2016	0505	20160505	PC	PC20160505	PC20160505	4	1	23	29	262	-4.8	-4.5	3.1	-4.8	16:00	16	0	0.00	16.00	16.00	88	-2.0	-2.9	0.6	-0.2
H280510	2016	0510	20160510	PC	PC20160510	PC20160510	2	2	20	77	229	-3.4	-3.0	1.7	-3.7	15:30	15	30	0.50	15.50	15.50	30	-4.8	-5.0	0.5	-0.6
H280516	2016	0516	20160516	PC	PC20160516	PC20160516	1	2	21	67	210	-3.2	-3.0	3.0		12:55	12	55	0.92	12.92	12.92	0	-3.0	-4.0	0.6	0.1
H280523	2016	0523	20160523	PC	PC20160523	PC20160523	1	1	25	44	119	-2.9	-2.5	-2.8	-3.5	12:25	12	25	0.42	12.42	12.42	0	-6.8	-7.3	-0.1	-0.9
H280530	2016	0530	20160530	PC	PC20160530	PC20160530	1	3	21	80	113	-2.9	-2.5	-2.2	-3	12:35	12	35	0.58	12.58	12.58	0	-4.5	-4.8	-0.1	-0.4
H280607	2016	0607	20160607	PC	PC20160607	PC20160607	2	2	22	67	165	-3.1	-2.5	0.9	-2.9	14:30	14	30	0.50	14.50	14.50	31	-4.9	-4.2	0.2	0.3
H280613	2016	0613	20160613	PC	PC20160613	PC20160613	1	3	23	86	127	-3.0	-2.5	-2.1		14:15	14	15	0.25	14.25	14.25	24	-4.6	-5.3	0.8	0.0
H280620	2016	0620	20160620	PC	PC20160620	PC20160620	1	2	25	75	214	-3.3	-3.0	1.3	-3.4	13:50	13	50	0.83	13.83	13.83	33	-4.7	-5.7	0.4	0.1
H280708	2016	0708	20160708	PC	PC20160708	PC20160708	5	2	27	71	201	-3.5	-3.0	3.9	-2	16:00	16	0	0.00	16.00	16.00	95	-6.2	-6.5	0.2	-0.2
H280711	2016	0711	20160711	PC	PC20160711	PC20160711	1	1	28	54	211	-3.2	-3.0	-0.8	-3.4	13:00	13	0	0.00	13.00	13.00	0	-3.9	-4.7	0.0	-0.3
H280719	2016	0719	20160719	PC	PC20160719	PC20160719	2	1	29	61	217	-3.5	-3.0	4.7	-3.6	15:40	15	40	0.67	15.67	15.67	90	-4.8	-5.4	1.1	-0.3
H280726	2016	0726	20160726	PC	PC20160726	PC20160726	2	3	25	76	186	-3.2	-3.0	1.5	-3.1	14:40	14	40	0.67	14.67	14.67	28	-4.3	-5.2	-0.6	-0.8
H280801	2016	0801	20160801	PC	PC20160801	PC20160801	1	1	30	50	210	-3.2	-3.0	0.3	-2.8	12:30	12	30	0.50	12.50	12.50	0	-5.8	-5.5	-0.5	-0.6
H280808	2016	0808	20160808	PC	PC20160808	PC20160808	1	1	31	54	109	-3.0	-2.5	2.3	-2.9	14:00	14	0	0.00	14.00	14.00	18	-3.0	-4.5	1.3	-0.4
H280816	2016	0816	20160816	PC	PC20160816	PC20160816	2	2	28	62	220	-3.5	-3.0	0.8	-3.7	15:10	15	10	0.17	15.17	15.17	76	-3.5	-3.8	0.8	-0.6
H280830	2016	0830	20160830	PC	PC20160830	PC20160830	2	1	27	46	163	-3.1	-2.5	2.3	-3.0	14:30	14	30	0.50	14.50	14.50	36	-5.3	-4.8	-0.5	-0.8
H280905	2016	0905	20160905	PC	PC20160905	PC20160905	1	2	27	86	109	-2.5	-2.0	1.7	-2.7	14:20	14	20	0.33	14.33	14.33	19	-4.7	-5.4	0.3	-0.6
H280913	2016	0913	20160913	PC	PC20160913	PC20160913	2	2	26	86	170	-3.3	-3.0	4.0	-3.4	15:10	15	10	0.17	15.17	15.17	57	-4.4	-5.4	-0.1	-0.9
H280920	2016	0920	20160920	PC	PC20160920	PC20160920	2	3	24	78	228	-3.9	-3.5	-3.3	-4	16:10	16	10	0.17	16.17	16.17	63	-6.0	-6.1	0.2	-0.7
H280926	2016	0926	20160926	PC	PC20160926	PC20160926	1	1	25	77	124	-3.0	-2.5	3.2	-2.8	14:30	14	30	0.50	14.50	14.50	28	-5.1	-5.0	-0.2	-0.6
H281011	2016	1011	20161011	PC	PC20161011	PC20161011	2	1	23	50	234	-4.1	-3.5	5.8	-3.6	15:20	15	20	0.33	15.33	15.33	130	-5.4	-5.1	-0.3	0.7
H281017	2016	1017	20161017	PC	PC20161017	PC20161017	1	2	21	81	137	-3.0	-2.5	1.8	-2.5	15:55	15	55	0.92	15.92	15.92	38	-5.7	-5.3	0.1	-0.4
H281025	2016	1025	20161025	PC	PC20161025	PC20161025	2	3	19	63	200	-3.5	-3.0	5.0	-3.6	15:05	15	5	0.08	15.08	15.08	103	-3.5	-5.0	-0.1	0.2
H281102	2016	1102	20161102	PC	PC20161102	PC20161102	3	1	17	43	258	-4.2	-4.0	2.1	-4.1	15:55	15	55	0.92	15.92	15.92	113	-1.8	-2.5	0.1	-0.1
H281107	2016	1107	20161107	PC	PC20161107	PC20161107	1	1	17	43	115	-3	-2.5	2.4	-3	14:45	14	45	0.75	14.75	14.75	27	-2.2	-2.4	0.3	-0.2
H281114	2016	1114	20161114	PC	PC20161114	PC20161114	1	1	17	63	155	-3.1	-2.5	-0.3	-2.7	14:25	14	25	0.42	14.42	14.42	17	-2.5	-2.8	-0.3	-0.6
H281121	2016	1121	20161121	PC	PC20161121	PC20161121	1	2	18	63	233	-4.5	-4.0	5.3	-4	15:15	15	15	0.25	15.25	15.25	56	-1.9	-3.5	0.1	0.3
H281128	2016	1128	20161128	PC	PC20161128	PC20161128	1	1	15	35	132	-2.9	-2.5	-1.4	-3.6	12:25	12	25	0.42	12.42	12.42	0	-0.1	-0.5	-0.1	-0.6
H281206	2016	1206	20161206	PC	PC20161206	PC20161206	2	1	16	40	165	-3.2	-3.0	2.6	-3.6	14:55	14	55	0.92	14.92	14.92	62	-2.2	-1.9	0.8	-0.8
H281213	2016	1213	20161213	PC	PC20161213	PC20161213	2	3	11	66	229	-4.0	-3.5	3.4	-3.3	15:50	15	50	0.83	15.83	15.83	103	-4.7	-5.3	0.5	-0.6
H281219	2016	1219	20161219	PC	PC20161219	PC20161219	1	1	13	48	154	-3.1	-2.5	3	-2.2	15:10	15	10	0.17	15.17	15.17	43	-0.5	-1.0	0.1	-0.4
H290104	2016	0104	20160104	PC	PC20160104	PC20160104	3	1	12	46	199	-3.4	-3.0	4.8	-4.1	16:10	16	10	0.17	16.17	16.17	68	-3.4	-5.1	0.4	-0.1
H290111	2016	0111	20160111	PC	PC20160111	PC20160111	3	2	12	41	228	-4.0	-3.5	5.3	-3.9	16:00	16	0	0.00	16.00	16.00	84	-3.1	-3.9	0.6	-0.5
H290116	2016	0116	20160116	PC	PC20160116	PC20160116	1	2	7	48	154	-3.1	-2.5	4.3	-3.1	15:05	15	5	0.08	15.08	15.08	38	-2.0	-2.6	0.4	-0.5
H290123	2016	0123	20160123	PC	PC20160123	PC20160123	1	1	8	43	127	-2.9	-2.5	1.4	-2.6	14:30	14	30	0.50	14.50	14.50	14	-3.0	-3.6	-0.5	-0.8
H290131	2016	0131	20160131	PC	PC20160131	PC20160131	2	1	10	34	253	-4.0	-3.5			16:05	16	5	0.08	16.08	16.08	92	-1.6	-3.1	1.3	-0.6
H290206	2016	0206	20160206	PC	PC20160206	PC20160206	1	2	10	51	236	-3.3	-3.0	3.6	-3.3	12:40	12	40	0.67	12.67	12.67	0	-2.8	-3.5	0.8	-0.4
H290213	2016	0213	20160213	PC	PC20160213	PC20160213	1	1	9	40	144	-3.0	-2.5	-2.3	-2.8	12:35	12	35	0.58	12.58	12.58	0	-1.7	-2.4	-0.2	-0.6
H290227	2016	0227	20160227	PC	PC20160227	PC20160227	1	1	12	36	126	-2.9	-2.5	-2.2	-2.8	12:40	12	40	0.67	12.67	12.67	0	-0.1	-0.6	0.3	-0.3
H290306	2016	0306	20160306	PC	PC20160306	PC20160306	1	3	12	69	234	-3.3	-3.0	2.5	-3.4	15:50	15	50	0.83	15.83	15.83	0	-3.2	-3.8	0.8	0.0
H290314	2016	0314	20160314	PC	PC20160314	PC20160314	2	1	14	37	196	-4.5	-4.0	3.1	-4.4	16:00	16	0	0.00	16.00	16.00	52	-4.0	-4.9	0.4	-0.8
H290321	2016	0321	20160321	PC	PC20160321	PC20160321	2	3	13	60	261	-5.0	-4.5	4.9	-5.4	16:20	16	20	0.33	16.33	16.33	154	-2.3	-3.5	1.3	1.2
H290327	2016	0327	20160327	PC	PC20160327	PC20160327	1	1	13	39	214	-3.2	-3.0	2.7	-3.5	12:40	12	40	0.67	12.67	12.67	0	-3.2	-3.9	0.2	-0.4

Sample ID	Outside temp.	Outside humidity	Carcass in the day	Carcass after noon	Loadeing completion	Preset temp. of system A	Preset temp. of system B	Room temp. at 16:30	Room temp. at next 8:00	Surface temp. on the ingate	Surface temp. on the recess	Core temp. on the ingate	Core temp. on the recess
PC20160404	18	86	110	0	12.58	-2.9	-2.5	-3.8	-2.1	-2.5	-3.0	0.3	-0.1
PC20160411	17	34	115	18	14.50	-3.0	-2.5	-2.9	-2.2	-0.7	-0.1	-0.2	0.1
PC20160421	18	81	169	95	15.83	-3.4	-3.0	2.6	-3.7	1.6	0.8	1.7	0.2
PC20160426	21	51	242	20	15.58	-4.4	-4.0	0.4	-4	1.0	1.5	0.4	0.5
PC20160505	23	29	262	88	16.00	-4.8	-4.5	3.1	-4.8	-2.0	-2.9	0.6	-0.2
PC20160510	20	77	229	30	15.50	-3.4	-3.0	1.7	-3.7	-4.8	-5.0	0.5	-0.6
PC20160516	21	67	210	0	12.92	-3.2	-3.0	3.0		-3.0	-4.0	0.6	0.1
PC20160523	25	44	119	0	12.42	-2.9	-2.5	-2.8	-3.5	-6.8	-7.3	-0.1	-0.9
PC20160530	21	80	113	0	12.58	-2.9	-2.5	-2.2	-3	-4.5	-4.8	-0.1	-0.4
PC20160607	22	67	165	31	14.50	-3.1	-2.5	0.9	-2.9	-4.9	-4.2	0.2	0.3
PC20160613	23	86	127	24	14.25	-3.0	-2.5	-2.1		-4.6	-5.3	0.8	0.0
PC20160620	25	75	214	33	13.83	-3.3	-3.0	1.3	-3.4	-4.7	-5.7	0.4	0.1
PC20160708	27	71	201	95	16.00	-3.5	-3.0	3.9	-2	-6.2	-6.5	0.2	-0.2
PC20160711	28	54	211	0	13.00	-3.2	-3.0	-0.8	-3.4	-3.9	-4.7	0.0	-0.3
PC20160719	29	61	217	90	15.67	-3.5	-3.0	4.7	-3.6	-4.8	-5.4	1.1	-0.3
PC20160726	25	76	186	28	14.67	-3.2	-3.0	1.5	-3.1	-4.3	-5.2	-0.6	-0.8
PC20160801	30	50	210	0	12.50	-3.2	-3.0	0.3	-2.8	-5.8	-5.5	-0.5	-0.6
PC20160808	31	54	109	18	14.00	-3.0	-2.5	2.3	-2.9	-3.0	-4.5	1.3	-0.4
PC20160816	28	62	220	76	15.17	-3.5	-3.0	0.8	-3.7	-3.5	-3.8	0.8	-0.6
PC20160830	27	46	163	36	14.50	-3.1	-2.5	2.3	-3.0	-5.3	-4.8	-0.5	-0.8
PC20160905	27	86	109	19	14.33	-2.5	-2.0	1.7	-2.7	-4.7	-5.4	0.3	-0.6
PC20160913	26	86	170	57	15.17	-3.3	-3.0	4.0	-3.4	-4.4	-5.4	-0.1	-0.9
PC20160920	24	78	228	63	16.17	-3.9	-3.5	-3.3	-4	-6.0	-6.1	0.2	-0.7
PC20160926	25	77	124	28	14.50	-3.0	-2.5	3.2	-2.8	-5.1	-5.0	-0.2	-0.6
PC20161011	23	50	234	130	15.33	-4.1	-3.5	5.8	-3.6	-5.4	-5.1	-0.3	0.7
PC20161017	21	81	137	38	15.92	-3.0	-2.5	1.8	-2.5	-5.7	-5.3	0.1	-0.4
PC20161025	19	63	200	103	15.08	-3.5	-3.0	5.0	-3.6	-3.5	-5.0	-0.1	0.2
PC20161102	17	43	258	113	15.92	-4.2	-4.0	2.1	-4.1	-1.8	-2.5	0.1	-0.1
PC20161107	17	43	115	27	14.75	-3	-2.5	2.4	-3	-2.2	-2.4	0.3	-0.2
PC20161114	17	63	155	17	14.42	-3.1	-2.5	-0.3	-2.7	-2.5	-2.8	-0.3	-0.6
PC20161121	18	63	233	56	15.25	-4.5	-4.0	5.3	-4	-1.9	-3.5	0.1	0.3
PC20161128	15	35	132	0	12.42	-2.9	-2.5	-1.4	-3.6	-0.1	-0.5	-0.1	-0.6
PC20161206	16	40	165	62	14.92	-3.2	-3.0	2.6	-3.6	-2.2	-1.9	0.8	-0.8
PC20161213	11	66	229	103	15.83	-4.0	-3.5	3.4	-3.3	-4.7	-5.3	0.5	-0.6
PC20161219	13	48	154	43	15.17	-3.1	-2.5	3	-2.2	-0.5	-1.0	0.1	-0.4
PC20160104	12	46	199	68	16.17	-3.4	-3.0	4.8	-4.1	-3.4	-5.1	0.4	-0.1
PC20160111	12	41	228	84	16.00	-4.0	-3.5	5.3	-3.9	-3.1	-3.9	0.6	-0.5
PC20160116	7	48	154	38	15.08	-3.1	-2.5	4.3	-3.1	-2.0	-2.6	0.4	-0.5
PC20160123	8	43	127	14	14.50	-2.9	-2.5	1.4	-2.6	-3.0	-3.6	-0.5	-0.8
PC20160131	10	34	253	92	16.08	-4.0	-3.5			-1.6	-3.1	1.3	-0.6
PC20160206	10	51	236	0	12.67	-3.3	-3.0	3.6	-3.3	-2.8	-3.5	0.8	-0.4
PC20160213	9	40	144	0	12.58	-3.0	-2.5	-2.3	-2.8	-1.7	-2.4	-0.2	-0.6
PC20160227	12	36	126	0	12.67	-2.9	-2.5	-2.2	-2.8	-0.1	-0.6	0.3	-0.3
PC20160306	12	69	234	0	15.83	-3.3	-3.0	2.5	-3.4	-3.2	-3.8	0.8	0.0
PC20160314	14	37	196	52	16.00	-4.5	-4.0	3.1	-4.4	-4.0	-4.9	0.4	-0.8
PC20160321	13	60	261	154	16.33	-5.0	-4.5	4.9	-5.4	-2.3	-3.5	1.3	1.2
PC20160327	13	39	214	0	12.67	-3.2	-3.0	2.7	-3.5	-3.2	-3.9	0.2	-0.4

Sample ID	Outside temp.	Outside humidity	Carcass in the day	Carcass after noon	Loading completion	Preset temp. of system A	Preset temp. of system B	Room temp. at 16:30	Room temp. at next 8:00	Surface temp. on the ingate	Surface temp. on the recess	Core temp. on the ingate	Core temp. on the recess
PC20160404	18	86	110	0	12.58	-2.9	-2.5	-3.8	-2.1	-2.5	-3.0	0.3	-0.1
PC20160411	17	34	115	18	14.50	-3.0	-2.5	-2.9	-2.2	-0.7	-0.1	-0.2	0.1
PC20160421	18	81	169	95	15.83	-3.4	-3.0	2.6	-3.7	1.6	0.8	1.7	0.2
PC20160426	21	51	242	20	15.58	-4.4	-4.0	0.4	-4	1.0	1.5	0.4	0.5
PC20160505	23	29	262	88	16.00	-4.8	-4.5	3.1	-4.8	-2.0	-2.9	0.6	-0.2
PC20160510	20	77	229	30	15.50	-3.4	-3.0	1.7	-3.7	-4.8	-5.0	0.5	-0.6
PC20160523	25	44	119	0	12.42	-2.9	-2.5	-2.8	-3.5	-6.8	-7.3	-0.1	-0.9
PC20160530	21	80	113	0	12.58	-2.9	-2.5	-2.2	-3	-4.5	-4.8	-0.1	-0.4
PC20160607	22	67	165	31	14.50	-3.1	-2.5	0.9	-2.9	-4.9	-4.2	0.2	0.3
PC20160620	25	75	214	33	13.83	-3.3	-3.0	1.3	-3.4	-4.7	-5.7	0.4	0.1
PC20160708	27	71	201	95	16.00	-3.5	-3.0	3.9	-2	-6.2	-6.5	0.2	-0.2
PC20160711	28	54	211	0	13.00	-3.2	-3.0	-0.8	-3.4	-3.9	-4.7	0.0	-0.3
PC20160719	29	61	217	90	15.67	-3.5	-3.0	4.7	-3.6	-4.8	-5.4	1.1	-0.3
PC20160726	25	76	186	28	14.67	-3.2	-3.0	1.5	-3.1	-4.3	-5.2	-0.6	-0.8
PC20160801	30	50	210	0	12.50	-3.2	-3.0	0.3	-2.8	-5.8	-5.5	-0.5	-0.6
PC20160808	31	54	109	18	14.00	-3.0	-2.5	2.3	-2.9	-3.0	-4.5	1.3	-0.4
PC20160816	28	62	220	76	15.17	-3.5	-3.0	0.8	-3.7	-3.5	-3.8	0.8	-0.6
PC20160830	27	46	163	36	14.50	-3.1	-2.5	2.3	-3.0	-5.3	-4.8	-0.5	-0.8
PC20160905	27	86	109	19	14.33	-2.5	-2.0	1.7	-2.7	-4.7	-5.4	0.3	-0.6
PC20160913	26	86	170	57	15.17	-3.3	-3.0	4.0	-3.4	-4.4	-5.4	-0.1	-0.9
PC20160920	24	78	228	63	16.17	-3.9	-3.5	-3.3	-4	-6.0	-6.1	0.2	-0.7
PC20160926	25	77	124	28	14.50	-3.0	-2.5	3.2	-2.8	-5.1	-5.0	-0.2	-0.6
PC20161011	23	50	234	130	15.33	-4.1	-3.5	5.8	-3.6	-5.4	-5.1	-0.3	0.7
PC20161017	21	81	137	38	15.92	-3.0	-2.5	1.8	-2.5	-5.7	-5.3	0.1	-0.4
PC20161025	19	63	200	103	15.08	-3.5	-3.0	5.0	-3.6	-3.5	-5.0	-0.1	0.2
PC20161102	17	43	258	113	15.92	-4.2	-4.0	2.1	-4.1	-1.8	-2.5	0.1	-0.1
PC20161107	17	43	115	27	14.75	-3	-2.5	2.4	-3	-2.2	-2.4	0.3	-0.2
PC20161114	17	63	155	17	14.42	-3.1	-2.5	-0.3	-2.7	-2.5	-2.8	-0.3	-0.6
PC20161121	18	63	233	56	15.25	-4.5	-4.0	5.3	-4	-1.9	-3.5	0.1	0.3
PC20161128	15	35	132	0	12.42	-2.9	-2.5	-1.4	-3.6	-0.1	-0.5	-0.1	-0.6
PC20161206	16	40	165	62	14.92	-3.2	-3.0	2.6	-3.6	-2.2	-1.9	0.8	-0.8
PC20161213	11	66	229	103	15.83	-4.0	-3.5	3.4	-3.3	-4.7	-5.3	0.5	-0.6
PC20161219	13	48	154	43	15.17	-3.1	-2.5	3	-2.2	-0.5	-1.0	0.1	-0.4
PC20160104	12	46	199	68	16.17	-3.4	-3.0	4.8	-4.1	-3.4	-5.1	0.4	-0.1
PC20160111	12	41	228	84	16.00	-4.0	-3.5	5.3	-3.9	-3.1	-3.9	0.6	-0.5
PC20160116	7	48	154	38	15.08	-3.1	-2.5	4.3	-3.1	-2.0	-2.6	0.4	-0.5
PC20160123	8	43	127	14	14.50	-2.9	-2.5	1.4	-2.6	-3.0	-3.6	-0.5	-0.8
PC20160206	10	51	236	0	12.67	-3.3	-3.0	3.6	-3.3	-2.8	-3.5	0.8	-0.4
PC20160213	9	40	144	0	12.58	-3.0	-2.5	-2.3	-2.8	-1.7	-2.4	-0.2	-0.6
PC20160227	12	36	126	0	12.67	-2.9	-2.5	-2.2	-2.8	-0.1	-0.6	0.3	-0.3
PC20160306	12	69	234	0	15.83	-3.3	-3.0	2.5	-3.4	-3.2	-3.8	0.8	0.0
PC20160314	14	37	196	52	16.00	-4.5	-4.0	3.1	-4.4	-4.0	-4.9	0.4	-0.8
PC20160321	13	60	261	154	16.33	-5.0	-4.5	4.9	-5.4	-2.3	-3.5	1.3	1.2
PC20160327	13	39	214	0	12.67	-3.2	-3.0	2.7	-3.5	-3.2	-3.9	0.2	-0.4

Sample ID	Outside temp.	Outside humidity	Carcass in the day	Carcass after noon	Loading completion	Preset temp. of system A	Preset temp. of system B	Preset temp.	Room temp. at 16:30	Room temp. at next 8:00	Surface temp. on the ingate	Surface temp. on the recess	Surface temp.	Core temp. on the ingate	Core temp. on the recess	Core temp.
PC20160404	18	86	110	0	12.58	−2.9	−2.5	−2.7	−3.8	−2.1	−2.5	−3.0	−2.8	0.3	−0.1	0.1
PC20160411	17	34	115	18	14.50	−3.0	−2.5	−2.8	−2.9	−2.2	−0.7	−0.1	−0.4	−0.2	0.1	−0.1
PC20160421	18	81	169	95	15.83	−3.4	−3.0	−3.2	2.6	−3.7	1.6	0.8	1.2	1.7	0.2	1.0
PC20160426	21	51	242	20	15.58	−4.4	−4.0	−4.2	0.4	−4	1.0	1.5	1.3	0.4	0.5	0.5
PC20160505	23	29	262	88	16.00	−4.8	−4.5	−4.7	3.1	−4.8	−2.0	−2.9	−2.5	0.6	−0.2	0.2
PC20160510	20	77	229	30	15.50	−3.4	−3.0	−3.2	1.7	−3.7	−4.8	−5.0	−4.9	0.5	−0.6	−0.1
PC20160523	25	44	119	0	12.42	−2.9	−2.5	−2.7	−2.8	−3.5	−6.8	−7.3	−7.1	−0.1	−0.9	−0.5
PC20160530	21	80	113	0	12.58	−2.9	−2.5	−2.7	−2.2	−3	−4.5	−4.8	−4.7	−0.1	−0.4	−0.3
PC20160607	22	67	165	31	14.50	−3.1	−2.5	−2.8	0.9	−2.9	−4.9	−4.2	−4.6	0.2	0.3	0.3
PC20160620	25	75	214	33	13.83	−3.3	−3.0	−3.2	1.3	−3.4	−4.7	−5.7	−5.2	0.4	0.1	0.3
PC20160708	27	71	201	95	16.00	−3.5	−3.0	−3.3	3.9	−2	−6.2	−6.5	−6.4	0.2	−0.2	0.0
PC20160711	28	54	211	0	13.00	−3.2	−3.0	−3.1	−0.8	−3.4	−3.9	−4.7	−4.3	0.0	−0.3	−0.2
PC20160719	29	61	217	90	15.67	−3.5	−3.0	−3.3	4.7	−3.6	−4.8	−5.4	−5.1	1.1	−0.3	0.4
PC20160726	25	76	186	28	14.67	−3.2	−3.0	−3.1	1.5	−3.1	−4.3	−5.2	−4.8	−0.6	−0.8	−0.7
PC20160801	30	50	210	0	12.50	−3.2	−3.0	−3.1	0.3	−2.8	−5.8	−5.5	−5.7	−0.5	−0.6	−0.6
PC20160808	31	54	109	18	14.00	−3.0	−2.5	−2.8	2.3	−2.9	−3.0	−4.5	−3.8	1.3	−0.4	0.5
PC20160816	28	62	220	76	15.17	−3.5	−3.0	−3.3	0.8	−3.7	−3.5	−3.8	−3.7	0.8	−0.6	0.1
PC20160830	27	46	163	36	14.50	−3.1	−2.5	−2.8	2.3	−3.0	−5.3	−4.8	−5.1	−0.5	−0.8	−0.7
PC20160905	27	86	109	19	14.33	−2.5	−2.0	−2.3	1.7	−2.7	−4.7	−5.4	−5.1	0.3	−0.6	−0.2
PC20160913	26	86	170	57	15.17	−3.3	−3.0	−3.2	4.0	−3.4	−4.4	−5.4	−4.9	−0.1	−0.9	−0.5
PC20160920	24	78	228	63	16.17	−3.9	−3.5	−3.7	−3.3	−4	−6.0	−6.1	−6.1	0.2	−0.7	−0.3
PC20160926	25	77	124	28	14.50	−3.0	−2.5	−2.8	3.2	−2.8	−5.1	−5.0	−5.1	−0.2	−0.6	−0.4
PC20161011	23	50	234	130	15.33	−4.1	−3.5	−3.8	5.8	−3.6	−5.4	−5.1	−5.3	−0.3	0.7	0.2
PC20161017	21	81	137	38	15.92	−3.0	−2.5	−2.8	1.8	−2.5	−5.7	−5.3	−5.5	0.1	−0.4	−0.2
PC20161025	19	63	200	103	15.08	−3.5	−3.0	−3.3	5.0	−3.6	−3.5	−5.0	−4.3	−0.1	0.2	0.1
PC20161102	17	43	258	113	15.92	−4.2	−4.0	−4.1	2.1	−4.1	−1.8	−2.5	−2.2	0.1	−0.1	0.0
PC20161107	17	43	115	27	14.75	−3	−2.5	−2.8	2.4	−3	−2.2	−2.4	−2.3	0.3	−0.2	0.1
PC20161114	17	63	155	17	14.42	−3.1	−2.5	−2.8	−0.3	−2.7	−2.5	−2.8	−2.7	−0.3	−0.6	−0.5
PC20161121	18	63	233	56	15.25	−4.5	−4.0	−4.3	5.3	−4	−1.9	−3.5	−2.7	0.1	0.3	0.2
PC20161128	15	35	132	0	12.42	−2.9	−2.5	−2.7	−1.4	−3.6	−0.1	−0.5	−0.3	−0.1	−0.6	−0.4
PC20161206	16	40	165	62	14.92	−3.2	−3.0	−3.1	2.6	−3.6	−2.2	−1.9	−2.1	0.8	−0.8	0.0
PC20161213	11	66	229	103	15.83	−4.0	−3.5	−3.8	3.4	−3.3	−4.7	−5.3	−5.0	0.5	−0.6	−0.1
PC20161219	13	48	154	43	15.17	−3.1	−2.5	−2.8	3	−2.2	−0.5	−1.0	−0.8	0.1	−0.4	−0.2
PC20160104	12	46	199	68	16.17	−3.4	−3.0	−3.2	4.8	−4.1	−3.4	−5.1	−4.3	0.4	−0.1	0.2
PC20160111	12	41	228	84	16.00	−4.0	−3.5	−3.8	5.3	−3.9	−3.1	−3.9	−3.5	0.6	−0.5	0.1
PC20160116	7	48	154	38	15.08	−3.1	−2.5	−2.8	4.3	−3.1	−2.0	−2.6	−2.3	0.4	−0.5	−0.1
PC20160123	8	43	127	14	14.50	−2.9	−2.5	−2.7	1.4	−2.6	−3.0	−3.6	−3.3	−0.5	−0.8	−0.7
PC20160206	10	51	236	0	12.67	−3.3	−3.0	−3.2	3.6	−3.3	−2.8	−3.5	−3.2	0.8	−0.4	0.2
PC20160213	9	40	144	0	12.58	−3.0	−2.5	−2.8	−2.3	−2.8	−1.7	−2.4	−2.1	−0.2	−0.6	−0.4
PC20160227	12	36	126	0	12.67	−2.9	−2.5	−2.7	−2.2	−2.8	−0.1	−0.6	−0.4	0.3	−0.3	0.0
PC20160306	12	69	234	0	15.83	−3.3	−3.0	−3.2	2.5	−3.4	−3.2	−3.8	−3.5	0.8	0.0	0.4
PC20160314	14	37	196	52	16.00	−4.5	−4.0	−4.3	3.1	−4.4	−4.0	−4.9	−4.5	0.4	−0.8	−0.2
PC20160321	13	60	261	154	16.33	−5.0	−4.5	−4.8	4.9	−5.4	−2.3	−3.5	−2.9	1.3	1.2	1.3
PC20160327	13	39	214	0	12.67	−3.2	−3.0	−3.1	2.7	−3.5	−3.2	−3.9	−3.6	0.2	−0.4	−0.1

Sample ID	Outside temp.	Outside humidity	Carcass in the day	Carcass after noon	Loading completion	Preset temp.	Room temp. at 16:30	Room temp. at next 8:00	Surface temp.	Core temp.
PC20160404	18	86	110	0	12.58	-2.7	-3.8	-2.1	-2.8	0.1
PC20160411	17	34	115	18	14.50	-2.8	-2.9	-2.2	-0.4	-0.1
PC20160421	18	81	169	95	15.83	-3.2	2.6	-3.7	1.2	1.0
PC20160426	21	51	242	20	15.58	-4.2	0.4	-4	1.3	0.5
PC20160505	23	29	262	88	16.00	-4.7	3.1	-4.8	-2.5	0.2
PC20160510	20	77	229	30	15.50	-3.2	1.7	-3.7	-4.9	-0.1
PC20160523	25	44	119	0	12.42	-2.7	-2.8	-3.5	-7.1	-0.5
PC20160530	21	80	113	0	12.58	-2.7	-2.2	-3	-4.7	-0.3
PC20160607	22	67	165	31	14.50	-2.8	0.9	-2.9	-4.6	0.3
PC20160620	25	75	214	33	13.83	-3.2	1.3	-3.4	-5.2	0.3
PC20160708	27	71	201	95	16.00	-3.3	3.9	-2	-6.4	0.0
PC20160711	28	54	211	0	13.00	-3.1	-0.8	-3.4	-4.3	-0.2
PC20160719	29	61	217	90	15.67	-3.3	4.7	-3.6	-5.1	0.4
PC20160726	25	76	186	28	14.67	-3.1	1.5	-3.1	-4.8	-0.7
PC20160801	30	50	210	0	12.50	-3.1	0.3	-2.8	-5.7	-0.6
PC20160808	31	54	109	18	14.00	-2.8	2.3	-2.9	-3.8	0.5
PC20160816	28	62	220	76	15.17	-3.3	0.8	-3.7	-3.7	0.1
PC20160830	27	46	163	36	14.50	-2.8	2.3	-3.0	-5.1	-0.7
PC20160905	27	86	109	19	14.33	-2.3	1.7	-2.7	-5.1	-0.2
PC20160913	26	86	170	57	15.17	-3.2	4.0	-3.4	-4.9	-0.5
PC20160920	24	78	228	63	16.17	-3.7	-3.3	-4	-6.1	-0.3
PC20160926	25	77	124	28	14.50	-2.8	3.2	-2.8	-5.1	-0.4
PC20161011	23	50	234	130	15.33	-3.8	5.8	-3.6	-5.3	0.2
PC20161017	21	81	137	38	15.92	-2.8	1.8	-2.5	-5.5	-0.2
PC20161025	19	63	200	103	15.08	-3.3	5.0	-3.6	-4.3	0.1
PC20161102	17	43	258	113	15.92	-4.1	2.1	-4.1	-2.2	0.0
PC20161107	17	43	115	27	14.75	-2.8	2.4	-3	-2.3	0.1
PC20161114	17	63	155	17	14.42	-2.8	-0.3	-2.7	-2.7	-0.5
PC20161121	18	63	233	56	15.25	-4.3	5.3	-4	-2.7	0.2
PC20161128	15	35	132	0	12.42	-2.7	-1.4	-3.6	-0.3	-0.4
PC20161206	16	40	165	62	14.92	-3.1	2.6	-3.6	-2.1	0.0
PC20161213	11	66	229	103	15.83	-3.8	3.4	-3.3	-5.0	-0.1
PC20161219	13	48	154	43	15.17	-2.8	3	-2.2	-0.8	-0.2
PC20160104	12	46	199	68	16.17	-3.2	4.8	-4.1	-4.3	0.2
PC20160111	12	41	228	84	16.00	-3.8	5.3	-3.9	-3.5	0.1
PC20160116	7	48	154	38	15.08	-2.8	4.3	-3.1	-2.3	-0.1
PC20160123	8	43	127	14	14.50	-2.7	1.4	-2.6	-3.3	-0.7
PC20160206	10	51	236	0	12.67	-3.2	3.6	-3.3	-3.2	0.2
PC20160213	9	40	144	0	12.58	-2.8	-2.3	-2.8	-2.1	-0.4
PC20160227	12	36	126	0	12.67	-2.7	-2.2	-2.8	-0.4	0.0
PC20160306	12	69	234	0	15.83	-3.2	2.5	-3.4	-3.5	0.4
PC20160314	14	37	196	52	16.00	-4.3	3.1	-4.4	-4.5	-0.2
PC20160321	13	60	261	154	16.33	-4.8	4.9	-5.4	-2.9	1.3
PC20160327	13	39	214	0	12.67	-3.1	2.7	-3.5	-3.6	-0.1
