# Supplementary material for: Dataset on causality analysis of chilling process in beef and pork carcasses using graphical modeling
Source: Data Brief. 2020 Jul 25;32:106075. doi: 10.1016/j.dib.2020.106075 (PMC7424210; doi:10.1016/j.dib.2020.106075)
Supplement: Supplementary file 3 — Supplementary Fig. S1a. The data processing of covariance selection of beef. [file mmc3.pdf]

Supplementary Fig. S1a.
The data processing of covariance selection of beef

Kuzuoka et al.

Sequence 1.

Partial correlation: less than 0.10

NFI: 0.90 or more

Juse SEM 因果分析編 - [Untitles]

ﾌｧｲﾙ(F) 編集(E) 表示(V) ﾚｲｱｳﾄ(L) 分析(A) ｳｨﾝﾄﾞｳ(W) ﾍﾙﾌﾟ(H)

- データ
  - データ
- 編集モデル
  - 共分散選択
  - 独立グラフ
- 保存モデル

	Outside	Outside	Number o	Completi	Preset t	Room te	Room te	Surface
Outsid	1.0000	0.3059	0.0976	−0.2262	−0.1004	−0.2297	−0.0291	−0.2527
Outsid	0.3059	1.0000	0.0253	−0.1984	−0.0422	−0.0626	−0.2021	−0.1009
Numb	0.0976	0.0253	1.0000	0.1498	−0.9156	0.5443	−0.4748	0.6334
Compl	−0.2262	−0.1984	0.1498	1.0000	−0.1055	0.2565	−0.2515	0.1250
Prese	−0.1004	−0.0422	−0.9156	−0.1055	1.0000	−0.4395	0.4607	−0.6230
Room	−0.2297	−0.0626	0.5443	0.2565	−0.4395	1.0000	−0.3656	0.3238
Room	−0.0291	−0.2021	−0.4748	−0.2515	0.4607	−0.3656	1.0000	−0.3213
Surfa	−0.2527	−0.1009	0.6334	0.1250	−0.6230	0.3238	−0.3213	1.0000
STD_D	1.0000	1.0000	1.0000	1.0000	1.0000	1.0000	1.0000	1.0000
_MEA	0.0000	0.0000	0.0000	0.0000	0.0000	0.0000	0.0000	0.0000

変数情報

ｻﾝﾌﾟﾙのﾏｽｸ

共分散選択

データ

警告・エラー 収束過程 分析情報

For Help, press F1

ここに入力して検索

19:22
2019/03/30

Juse SEM 因果分析編 - [Untitled]

ﾌｧｲﾙ(F) 編集(E) 表示(V) ﾚｲｱｳﾄ(L) 分析(A) ｳｨﾝﾄﾞｳ(W) ﾍﾙﾌﾟ(H)

- データ
  - データ
- 編集モデル
  - 共分散選択
  - 独立グラフ
- 保存モデル

データ数：44

《モデル全体》

フルモデルとの比較　：逸脱度＝－　　自由度＝－　　P値＝－

適合度指標　：NFI＝1.000

《第1群》

フルモデルとの比較　：逸脱度＝－　　自由度＝－　　P値＝－

直前のモデルとの比較　：逸脱度＝－　　自由度＝－　　P値＝－

適合度指標　：GFI＝1.000　AGFI＝1.000　NFI＝1.000　SRMR＝0.000

下三角：偏相関係数　　上三角：相関係数の残差

	Outside te	Outside hu	Number of	Completion	Preset tem	Room temp	Room temp	Surface te
V1 Outside temperature	***							
V2 Outside humidity	0.30498	***						
V3 Number of carcass	0.09442	-0.00481	***					
V4 Completion of loading				***				
V5 Preset temp.					***			
V6 Room temp. at 16:30						***		
V7 Room temp. at next 8:00							***	
V8 Surface temp.								***

共分散選択

前の群へ
次の群へ
切断
接続
逐次自動切断
線の凍結
選択履歴
モデルの保存
独立グラフ

警告・エラー　収束過程　分析情報

For Help, press F1

ここに入力して検索

19:25
2019/03/30

Juse SEM 因果分析編 - [Untitled]

ﾌｧｲﾙ(F) 編集(E) 表示(V) ﾚｲｱｳﾄ(L) 分析(A) ｳｨﾝﾄﾞｳ(W) ﾍﾙﾌﾟ(H)

データ
データ
編集モデル
共分散選択
独立グラフ
保存モデル

データ数：44

《モデル全体》

フルモデルとの比較　：　逸脱度＝0.422　自由度＝2　P値＝0.8097

適合度指標　：　NFI＝0.997

《第1群》

フルモデルとの比較　：　逸脱度＝0.422　自由度＝2　P値＝0.8097

直前のモデルとの比較　：　逸脱度＝0.421　自由度＝1　P値＝0.5164

適合度指標　：　GFI＝0.994　AGFI＝0.981　NFI＝0.911　SRMR＝0.041

下三角：偏相関係数　上三角：相関係数の残差

	Outside te	Outside hu	Number of	Completion	Preset tem	Room temp	Room temp	Surface te
V1 Outside temperature	***		0.09759					
V2 Outside humidity	0.30590	***	0.02530					
V3 Number of carcass	0.00001	0.00000	***					
V4 Completion of loading				***				
V5 Preset temp.					***			
V6 Room temp. at 16:30						***		
V7 Room temp. at next 8:00							***	
V8 Surface temp.								***

共分散選択

前の群へ
次の群へ
切断
接続
逐次自動切断
線の凍結
選択履歴
モデルの保存
独立グラフ

警告・エラー　収束過程　分析情報

For Help, press F1

ここに入力して検索

19:26
2019/03/30

Juse SEM 因果分析編 - [Untitled]

ﾌｧｲﾙ(F)　編集(E)　表示(V)　ﾚｲｱｳﾄ(L)　分析(A)　ｳｨﾝﾄﾞｳ(W)　ﾍﾙﾌﾟ(H)

データ
　データ
編集モデル
　共分散選択
　独立グラフ
保存モデル

データ数：44
《モデル全体》
フルモデルとの比較　：逸脱度＝0.422　自由度＝2　P値＝0.8097
適合度指標　：NFI＝0.997

《第2群》
フルモデルとの比較　：逸脱度＝－　自由度＝－　P値＝－
直前のモデルとの比較　：逸脱度＝－　自由度＝－　P値＝－
適合度指標　：GFI＝1.000　AGFI＝1.000　NFI＝1.000　SRMR＝0.000

下三角：偏相関係数　上三角：相関係数の残差

	Outside te	Outside hu	Number of	Completion	Preset tem	Room temp	Room temp	Surface te
V1 Outside temperature	***							
V2 Outside humidity		***						
V3 Number of carcass			***					
V4 Completion of loading	-0.14339	-0.14211	0.06972	***				
V5 Preset temp.	0.05152	-0.03696	-0.89198	0.04604	***			
V6 Room temp. at 16:30	-0.30491	0.03980	0.40367	0.13376	0.16840	***		
V7 Room temp. at next 8:00							***	
V8 Surface temp.								***

前の群へ
次の群へ
切断
接続
逐次自動切断
線の凍結
選択履歴
モデルの保存
独立グラフ

共分散選択

警告・エラー　収束過程　分析情報

For Help, press F1

ここに入力して検索
19:26
2019/03/30

Juse SEM 因果分析編 - [Untitled]

ﾌｧｲﾙ(F) 編集(E) 表示(V) ﾚｲｱｳﾄ(L) 分析(A) ｳｨﾝﾄﾞｳ(W) ﾍﾙﾌﾟ(H)

データ
- データ

編集モデル
- 共分散選択
- 独立グラフ

保存モデル

データ数：44

《モデル全体》

フルモデルとの比較　：逸脱度＝0.897　自由度＝7　P値＝0.9963

適合度指標　：NFI＝0.995

《第2群》

フルモデルとの比較　：逸脱度＝0.475　自由度＝5　P値＝0.9930

直前のモデルとの比較　：逸脱度＝0.194　自由度＝1　P値＝0.6593

適合度指標　：GFI＝0.996　AGFI＝0.985　NFI＝0.996　SRMR＝0.015

下三角：偏相関係数　上三角：相関係数の残差

	Outside te	Outside hu	Number of	Completion	Preset tem	Room temp	Room temp	Surface te
V1 Outside temperature	***				0.01262			
V2 Outside humidity		***			-0.01023	0.02889		
V3 Number of carcass			***	0.05061				
V4 Completion of loading	-0.12793	-0.13765	0.00001	***	-0.03162			
V5 Preset temp.	-0.00001	0.00000	-0.89069	-0.00000	***			
V6 Room temp. at 16:30	-0.28426	0.00000	0.40266	0.17318	0.16194	***		
V7 Room temp. at next 8:00							***	
V8 Surface temp.								***

前の群へ
次の群へ
切断
接続
逐次自動切断
線の凍結
選択履歴
モデルの保存
独立グラフ

共分散選択

警告・エラー　収束過程　分析情報

For Help, press F1

ここに入力して検索

19:27
2019/03/30

Juse SEM 因果分析編 - [Untitled]

ﾌｧｲﾙ(F) 編集(E) 表示(V) ﾚｲｱｳﾄ(L) 分析(A) ｳｨﾝﾄﾞｳ(W) ﾍﾙﾌﾟ(H)

データ
データ
編集モデル
共分散選択
独立グラフ
保存モデル

データ数：44

《モデル全体》

フルモデルとの比較　：逸脱度＝0.897　自由度＝7　P値＝0.9963

適合度指標　：NFI＝0.995

《第3群》

フルモデルとの比較　：逸脱度＝－　自由度＝－　P値＝－

直前のモデルとの比較　：逸脱度＝－　自由度＝－　P値＝－

適合度指標　：GFI＝1.000　AGFI＝1.000　NFI＝1.000　SRMR＝0.000

下三角：偏相関係数　上三角：相関係数の残差

	Outside te	Outside hu	Number of	Completion	Preset tem	Room temp	Room temp	Surface te
V1 Outside temperature	***							
V2 Outside humidity		***						
V3 Number of carcass			***					
V4 Completion of loading				***				
V5 Preset temp.					***			
V6 Room temp. at 16:30						***		
V7 Room temp. at next 8:00	-0.02666	-0.26950	-0.04692	-0.24306	0.09987	-0.14273	***	
V8 Surface temp.	-0.42405	-0.05558	0.27321	-0.05466	-0.12617	-0.17468	-0.06318	***

共分散選択

前の群へ
次の群へ
切断
接続
逐次自動切断
線の凍結
選択履歴
モデルの保存
独立グラフ

警告・エラー　収束過程　分析情報

For Help, press F1

ここに入力して検索

19:28
2019/03/30

Juse SEM 因果分析編 - [Untitled]

ﾌｧｲﾙ(F) 編集(E) 表示(V) ﾚｲｱｳﾄ(L) 分析(A) ｳｨﾝﾄﾞｳ(W) ﾍﾙﾌﾟ(H)

- データ
  - データ
- 編集モデル
  - 共分散選択
  - 独立グラフ
- 保存モデル

データ数：44

《モデル全体》

フルモデルとの比較　：　逸脱度＝1.401　自由度＝12　P値＝0.9999

適合度指標　：　NFI＝0.991

《第3群》

フルモデルとの比較　：　逸脱度＝0.504　自由度＝5　P値＝0.9920

直前のモデルとの比較　：　逸脱度＝0.203　自由度＝1　P値＝0.6526

適合度指標　：　GFI＝0.997　AGFI＝0.979　NFI＝0.997　SRMR＝0.009

下三角：偏相関係数　上三角：相関係数の残差

	Outside te	Outside hu	Number of	Completion	Preset tem	Room temp	Room temp	Surface te
V1 Outside temperature	***						-0.00826	
V2 Outside humidity		***						-0.02263
V3 Number of carcass			***				-0.02052	
V4 Completion of loading				***				-0.02291
V5 Preset temp.					***			
V6 Room temp. at 16:30						***		
V7 Room temp. at next 8:00	-0.00001	-0.26699	0.00000	-0.24282	0.16620	-0.15491	***	-0.03307
V8 Surface temp.	-0.42604	0.00000	0.27553	0.00000	-0.13170	-0.17128	0.00000	***

前の群へ　次の群へ　切断　接続　逐次自動切断　線の凍結　選択履歴　モデルの保存　独立グラフ

共分散選択

警告・エラー　収束過程　分析情報

For Help, press F1

ここに入力して検索　19:29　2019/03/30

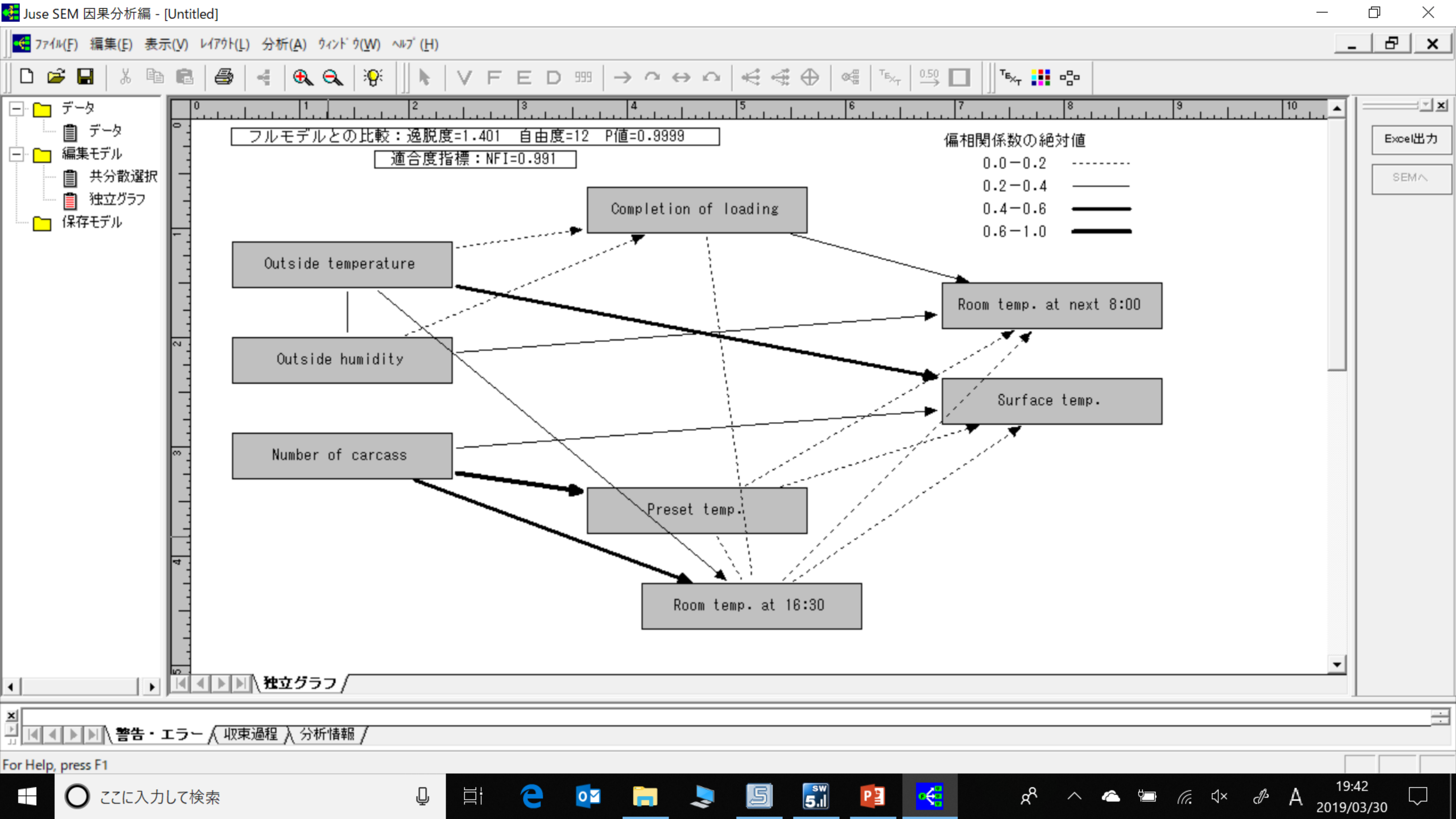
Juse SEM 因果分析編 - [Untitled]
ﾌｧｲﾙ(F) 編集(E) 表示(V) ﾚｲｱｳﾄ(L) 分析(A) ｳｨﾝﾄﾞｳ(W) ﾍﾙﾌﾟ(H)
データ
データ
編集モデル
共分散選択
独立グラフ
保存モデル
フルモデルとの比較：逸脱度=1.401　自由度=12　P値=0.9999
適合度指標：NFI=0.991
偏相関係数の絶対値
0.0－0.2
0.2－0.4
0.4－0.6
0.6－1.0
Completion of loading
Outside temperature
Room temp. at next 8:00
Outside humidity
Surface temp.
Number of carcass
Preset temp.
Room temp. at 16:30
Excel出力
SEMへ
独立グラフ
警告・エラー
収束過程
分析情報
For Help, press F1
ここに入力して検索
19:42
2019/03/30

Juse SEM 因果分析編 - モデル相関係数行列_第3群.ess

ﾌｧｲﾙ(F) 編集(E) 表示(V) ﾃﾞｰﾀ(D) 分析(A) ｳｨﾝﾄﾞｳ(W) ﾍﾙﾌﾟ(H)

標本相関係数行列.ess

	Outside	Outside	Number o	Completi	Preset t	Room te	Room te	Surface
Outsid	1.0000	0.3059	0.0976	−0.2262	−0.1004	−0.2297	−0.0291	−0.2527
Outsid	0.3059	1.0000	0.0253	−0.1984	−0.0422	−0.0626	−0.2021	−0.1009
Numb	0.0976	0.0253	1.0000	0.1498	−0.9156	0.5443	−0.4748	0.6334
Compl	−0.2262	−0.1984	0.1498	1.0000	−0.1055	0.2565	−0.2515	0.1250
Prese	−0.1004	−0.0422	−0.9156	−0.1055	1.0000	−0.4395	0.4607	−0.6230
Room	−0.2297	−0.0626	0.5443	0.2565	−0.4395	1.0000	−0.3656	0.3238
Room	−0.0291	−0.2021	−0.4748	−0.2515	0.4607	−0.3656	1.0000	−0.3213
Surfa	−0.2527	−0.1009	0.6334	0.1250	−0.6230	0.3238	−0.3213	1.0000
STD_D	1.0000	1.0000	1.0000	1.0000	1.0000	1.0000	1.0000	1.0000
_MEA	0.0000	0.0000	0.0000	0.0000	0.0000	0.0000	0.0000	0.0000

モデル相関係数行列_第3群.ess

	Outside	Outside	Number o	Completi	Preset t	Room te	Room te	Surface
Outsid	1.0000	0.3059	0.0976	−0.2262	−0.1004	−0.2297	−0.0208	−0.2527
Outsid	0.3059	1.0000	0.0253	−0.1984	−0.0422	−0.0626	−0.2021	−0.0783
Numb	0.0976	0.0253	1.0000	0.1498	−0.9156	0.5443	−0.4543	0.6334
Compl	−0.2262	−0.1984	0.1498	1.0000	−0.1055	0.2565	−0.2515	0.1479
Prese	−0.1004	−0.0422	−0.9156	−0.1055	1.0000	−0.4395	0.4607	−0.6230
Room	−0.2297	−0.0626	0.5443	0.2565	−0.4395	1.0000	−0.3656	0.3238
Room	−0.0208	−0.2021	−0.4543	−0.2515	0.4607	−0.3656	1.0000	−0.2882
Surfa	−0.2527	−0.0783	0.6334	0.1479	−0.6230	0.3238	−0.2882	1.0000
STD_D	1.0000	1.0000	1.0000	1.0000	1.0000	1.0000	1.0000	1.0000
_MEA	0.0000	0.0000	0.0000	0.0000	0.0000	0.0000	0.0000	0.0000

パス図

変数情報

ﾓﾃﾞﾙﾌｧｲﾙ作成

警告・エラー　収束過程　分析情報

For Help, press F1

ここに入力して検索

20:00
2019/03/30

Sequence 2.

Partial correlation: less than 0.20

NFI: 0.90 or more

Juse SEM 因果分析編 - [Untitles]

ﾌｧｲﾙ(F) 編集(E) 表示(V) ﾚｲｱｳﾄ(L) 分析(A) ｳｨﾝﾄﾞｳ(W) ﾍﾙﾌﾟ(H)

- データ
  - データ
- 編集モデル
  - 共分散選択
  - 独立グラフ
- 保存モデル

	Outside	Outside	Number o	Completi	Preset t	Room te	Room te	Surface
Outsid	1.0000	0.3059	0.0976	−0.2262	−0.1004	−0.2297	−0.0291	−0.2527
Outsid	0.3059	1.0000	0.0253	−0.1984	−0.0422	−0.0626	−0.2021	−0.1009
Numb	0.0976	0.0253	1.0000	0.1498	−0.9156	0.5443	−0.4748	0.6334
Compl	−0.2262	−0.1984	0.1498	1.0000	−0.1055	0.2565	−0.2515	0.1250
Prese	−0.1004	−0.0422	−0.9156	−0.1055	1.0000	−0.4395	0.4607	−0.6230
Room	−0.2297	−0.0626	0.5443	0.2565	−0.4395	1.0000	−0.3656	0.3238
Room	−0.0291	−0.2021	−0.4748	−0.2515	0.4607	−0.3656	1.0000	−0.3213
Surfa	−0.2527	−0.1009	0.6334	0.1250	−0.6230	0.3238	−0.3213	1.0000
STD_D	1.0000	1.0000	1.0000	1.0000	1.0000	1.0000	1.0000	1.0000
_MEA	0.0000	0.0000	0.0000	0.0000	0.0000	0.0000	0.0000	0.0000

変数情報

ｻﾝﾌﾟﾙのﾏｽｸ

共分散選択

データ

警告・エラー　収束過程　分析情報

For Help, press F1

ここに入力して検索

20:17
2019/03/30

Juse SEM 因果分析編 - [Untitled]

ﾌｧｲﾙ(F) 編集(E) 表示(V) ﾚｲｱｳﾄ(L) 分析(A) ｳｨﾝﾄﾞｳ(W) ﾍﾙﾌﾟ(H)

データ
データ
編集モデル
共分散選択
独立グラフ
保存モデル

データ数：44
《モデル全体》
フルモデルとの比較　：逸脱度＝ー　自由度＝ー　P値＝ー
適合度指標　：NFI＝1.000

《第1群》
フルモデルとの比較　：逸脱度＝ー　自由度＝ー　P値＝ー
直前のモデルとの比較　：逸脱度＝ー　自由度＝ー　P値＝ー
適合度指標　：GFI＝1.000　AGFI＝1.000　NFI＝1.000　SRMR＝0.000

下三角：偏相関係数　上三角：相関係数の残差

	Outside te	Outside hu	Number of	Completion	Preset tem	Room temp	Room temp	Surface te
V1 Outside temperature	***							
V2 Outside humidity	0.30498	***						
V3 Number of carcass	0.09442	-0.00481	***					
V4 Completion of loading				***				
V5 Preset temp.					***			
V6 Room temp. at 16:30						***		
V7 Room temp. at next 8:00							***	
V8 Surface temp.								***

共分散選択

前の群へ
次の群へ
切断
接続
逐次自動切断
線の凍結
選択履歴
モデルの保存
独立グラフ

警告・エラー　収束過程　分析情報

For Help, press F1

ここに入力して検索

20:19
2019/03/30

Juse SEM 因果分析編 - [Untitled]

データ数：44
《モデル全体》
フルモデルとの比較　：逸脱度＝0.422　自由度＝2　P値＝0.8097
適合度指標　：NFI＝0.997

《第1群》
フルモデルとの比較　：逸脱度＝0.422　自由度＝2　P値＝0.8097
直前のモデルとの比較　：逸脱度＝0.421　自由度＝1　P値＝0.5164
適合度指標　：GFI＝0.994　AGFI＝0.981　NFI＝0.911　SRMR＝0.041

下三角：偏相関係数　上三角：相関係数の残差

	Outside te	Outside hu	Number of	Completion	Preset tem	Room temp	Room temp	Surface te
V1 Outside temperature	***		0.09759					
V2 Outside humidity	0.30590	***	0.02530					
V3 Number of carcass	0.00001	0.00000	***					
V4 Completion of loading				***				
V5 Preset temp.					***			
V6 Room temp. at 16:30						***		
V7 Room temp. at next 8:00							***	
V8 Surface temp.								***

共分散選択

For Help, press F1

Juse SEM 因果分析編 - [Untitled]

ﾌｧｲﾙ(F) 編集(E) 表示(V) ﾚｲｱｳﾄ(L) 分析(A) ｳｨﾝﾄﾞｳ(W) ﾍﾙﾌﾟ(H)

- データ
  - データ
- 編集モデル
  - 共分散選択
  - 独立グラフ
- 保存モデル

データ数：44

《モデル全体》

フルモデルとの比較　：逸脱度＝0.422　自由度＝2　P値＝0.8097

適合度指標　：NFI＝0.997

《第2群》

フルモデルとの比較　：逸脱度＝ー　自由度＝ー　P値＝ー

直前のモデルとの比較　：逸脱度＝ー　自由度＝ー　P値＝ー

適合度指標　：GFI＝1.000　AGFI＝1.000　NFI＝1.000　SRMR＝0.000

下三角：偏相関係数　上三角：相関係数の残差

	Outside te	Outside hu	Number of	Completion	Preset tem	Room temp	Room temp	Surface te
V1 Outside temperature	***							
V2 Outside humidity		***						
V3 Number of carcass			***					
V4 Completion of loading	−0.14339	−0.14211	0.06972	***				
V5 Preset temp.	0.05152	−0.03696	−0.89198	0.04604	***			
V6 Room temp. at 16:30	−0.30491	0.03980	0.40367	0.13376	0.16840	***		
V7 Room temp. at next 8:00							***	
V8 Surface temp.								***

共分散選択

前の群へ | 次の群へ | 切断 | 接続 | 逐次自動切断 | 線の凍結 | 選択履歴 | モデルの保存 | 独立グラフ

警告・エラー | 収束過程 | 分析情報

For Help, press F1

ここに入力して検索

20:20
2019/03/30

Juse SEM 因果分析編 - [Untitled]

ﾌｧｲﾙ(F) 編集(E) 表示(V) ﾚｲｱｳﾄ(L) 分析(A) ｳｨﾝﾄﾞｳ(W) ﾍﾙﾌﾟ(H)

- データ
  - データ
- 編集モデル
  - 共分散選択
  - 独立グラフ
- 保存モデル

データ数：44

《モデル全体》

フルモデルとの比較　：　逸脱度＝4.541　自由度＝10　P値＝0.9197

適合度指標　：　NFI＝0.972

《第2群》

フルモデルとの比較　：　逸脱度＝4.118　自由度＝8　P値＝0.8463

直前のモデルとの比較　：　逸脱度＝1.500　自由度＝1　P値＝0.2206

適合度指標　：　GFI＝0.971　AGFI＝0.923　NFI＝0.964　SRMR＝0.056

下三角：偏相関係数　　上三角：相関係数の残差

	Outside te	Outside hu	Number of	Completion	Preset tem	Room temp	Room temp	Surface te
V1 Outside temperature	***			-0.16727	-0.01103			
V2 Outside humidity		***		-0.17970	-0.01903	0.01027		
V3 Number of carcass			***	0.01020				
V4 Completion of loading	-0.00000	-0.00000	0.00000	***	0.02231			
V5 Preset temp.	-0.00001	-0.00000	-0.87839	0.00001	***	0.05886		
V6 Room temp. at 16:30	-0.31746	-0.00000	0.27370	0.20502	-0.00000	***		
V7 Room temp. at next 8:00							***	
V8 Surface temp.								***

前の群へ

次の群へ

切断

接続

逐次自動切断

線の凍結

選択履歴

モデルの保存

独立グラフ

共分散選択

02_牛_平均

警告・エラー　収束過程　分析情報

For Help, press F1

20:21 2019/03/30

Juse SEM 因果分析編 - [Untitled]

データ数：44

《モデル全体》

フルモデルとの比較　：逸脱度＝4.541　自由度＝10　P値＝0.9197

適合度指標　：NFI＝0.972

《第3群》

フルモデルとの比較　：逸脱度＝－　自由度＝－　P値＝－

直前のモデルとの比較　：逸脱度＝－　自由度＝－　P値＝－

適合度指標　：GFI＝1.000　AGFI＝1.000　NFI＝1.000　SRMR＝0.000

下三角：偏相関係数　上三角：相関係数の残差

	Outside te	Outside hu	Number of	Completion	Preset tem	Room temp	Room temp	Surface te
V1 Outside temperature	***							
V2 Outside humidity		***						
V3 Number of carcass			***					
V4 Completion of loading				***				
V5 Preset temp.					***			
V6 Room temp. at 16:30						***		
V7 Room temp. at next 8:00	−0.02666	−0.26950	−0.04692	−0.24306	0.09987	−0.14273	***	
V8 Surface temp.	−0.42405	−0.05558	0.27321	−0.05466	−0.12617	−0.17468	−0.06318	***

For Help, press F1

Juse SEM 因果分析編 - [Untitled]

データ数：44

《モデル全体》

フルモデルとの比較　：　逸脱度＝19.027　自由度＝19　P値＝0.4551

適合度指標　：　NFI＝0.884

《第3群》

フルモデルとの比較　：　逸脱度＝14.486　自由度＝9　P値＝0.1060

直前のモデルとの比較　：　逸脱度＝10.006　自由度＝1　P値＝0.0016

適合度指標　：　GFI＝0.932　AGFI＝0.726　NFI＝0.912　SRMR＝0.123

下三角：偏相関係数　　上三角：相関係数の残差

	Outside te	Outside hu	Number of	Completion	Preset tem	Room temp	Room temp	Surface te
V1 Outside temperature	***						−0.01751	
V2 Outside humidity		***						−0.02057
V3 Number of carcass			***				−0.42265	
V4 Completion of loading				***				−0.04635
V5 Preset temp.					***		0.41756	−0.04656
V6 Room temp. at 16:30						***	−0.30414	−0.11077
V7 Room temp. at next 8:00	−0.00000	−0.25585	−0.00001	−0.29193	0.00001	0.00000	***	−0.29033
V8 Surface temp.	−0.36976	−0.00000	0.32401	−0.00000	−0.00000	−0.00001	0.00000	***

共分散選択

For Help, press F1

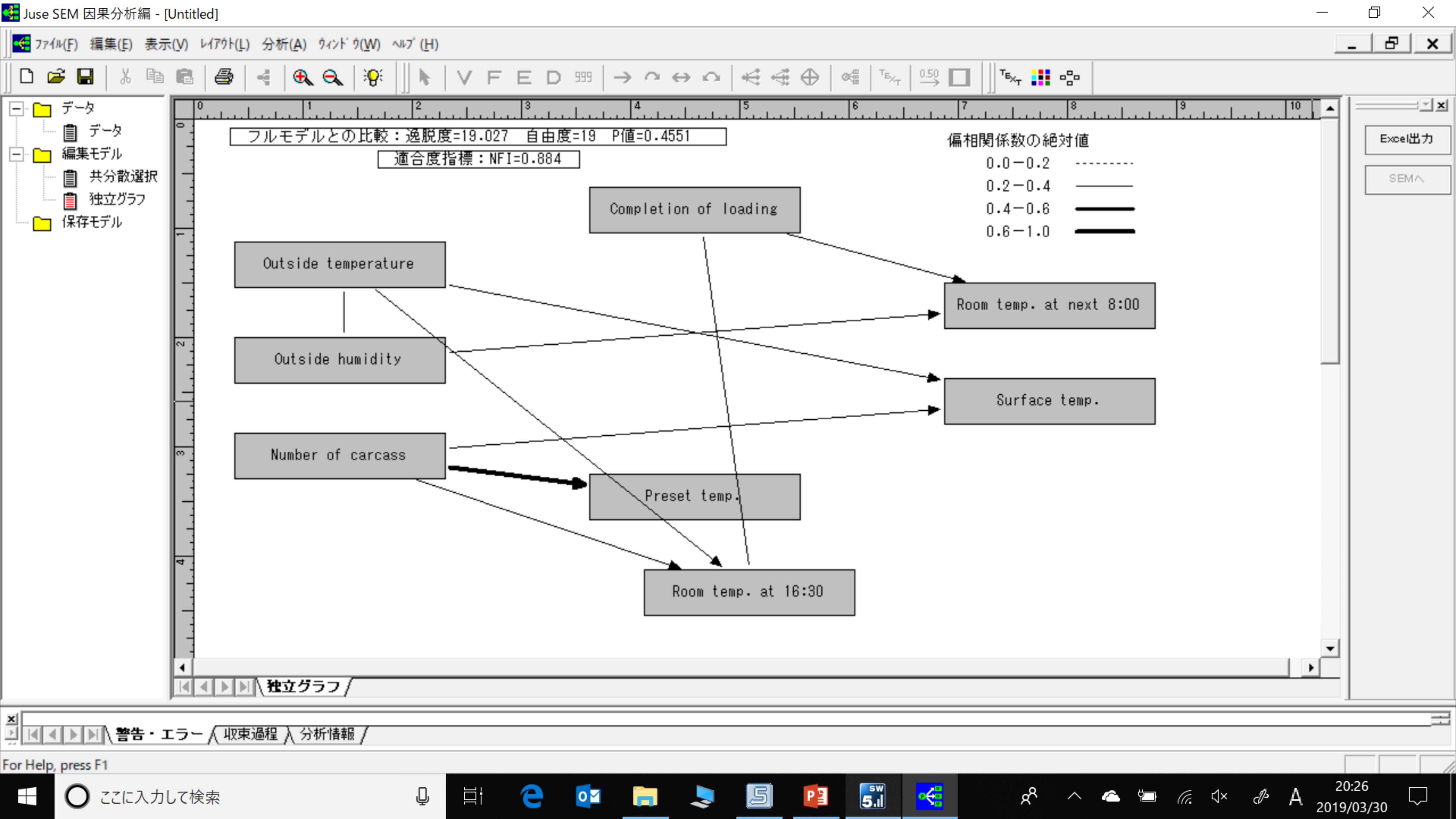
Juse SEM 因果分析編 - [Untitled]
ﾌｧｲﾙ(F) 編集(E) 表示(V) ﾚｲｱｳﾄ(L) 分析(A) ｳｨﾝﾄﾞｳ(W) ﾍﾙﾌﾟ(H)
データ
データ
編集モデル
共分散選択
独立グラフ
保存モデル
フルモデルとの比較：適脱度=19.027 自由度=19 P値=0.4551
適合度指標：NFI=0.884
偏相関係数の絶対値
0.0－0.2
0.2－0.4
0.4－0.6
0.6－1.0
Completion of loading
Outside temperature
Outside humidity
Number of carcass
Preset temp.
Room temp. at 16:30
Room temp. at next 8:00
Surface temp.
Excel出力
SEMへ
独立グラフ
警告・エラー
収束過程
分析情報
For Help, press F1
ここに入力して検索
20:26
2019/03/30

Juse SEM 因果分析編 - モデル相関係数行列_第3群.ess

ﾌｧｲﾙ(F) 編集(E) 表示(V) ﾃﾞｰﾀ(D) 分析(A) ｳｨﾝﾄﾞｳ(W) ﾍﾙﾌﾟ(H)

標本相関係数行列.ess

	Outside	Outside	Number o	Completi	Preset t	Room te	Room te	Surface
Outsid	1.0000	0.3059	0.0976	−0.2262	−0.1004	−0.2297	−0.0291	−0.2527
Outsid	0.3059	1.0000	0.0253	−0.1984	−0.0422	−0.0626	−0.2021	−0.1009
Numb	0.0976	0.0253	1.0000	0.1498	−0.9156	0.5443	−0.4748	0.6334
Compl	−0.2262	−0.1984	0.1498	1.0000	−0.1055	0.2565	−0.2515	0.1250
Prese	−0.1004	−0.0422	−0.9156	−0.1055	1.0000	−0.4395	0.4607	−0.6230
Room	−0.2297	−0.0626	0.5443	0.2565	−0.4395	1.0000	−0.3656	0.3238
Room	−0.0291	−0.2021	−0.4748	−0.2515	0.4607	−0.3656	1.0000	−0.3213
Surfa	−0.2527	−0.1009	0.6334	0.1250	−0.6230	0.3238	−0.3213	1.0000
STD_D	1.0000	1.0000	1.0000	1.0000	1.0000	1.0000	1.0000	1.0000
_MEA	0.0000	0.0000	0.0000	0.0000	0.0000	0.0000	0.0000	0.0000

モデル相関係数行列_第3群.ess

	Outside	Outside	Number o	Completi	Preset t	Room te	Room te	Surface
Outsid	1.0000	0.3059	0.0976	−0.2262	−0.1004	−0.2297	−0.0116	−0.2527
Outsid	0.3059	1.0000	0.0253	−0.1984	−0.0422	−0.0626	−0.2021	−0.0803
Numb	0.0976	0.0253	1.0000	0.1498	−0.9156	0.5443	−0.0522	0.6334
Compl	−0.2262	−0.1984	0.1498	1.0000	−0.1055	0.2565	−0.2515	0.1714
Prese	−0.1004	−0.0422	−0.9156	−0.1055	1.0000	−0.4395	0.0431	−0.5764
Room	−0.2297	−0.0626	0.5443	0.2565	−0.4395	1.0000	−0.0615	0.4346
Room	−0.0116	−0.2021	−0.0522	−0.2515	0.0431	−0.0615	1.0000	−0.0310
Surfa	−0.2527	−0.0803	0.6334	0.1714	−0.5764	0.4346	−0.0310	1.0000
STD_D	1.0000	1.0000	1.0000	1.0000	1.0000	1.0000	1.0000	1.0000
_MEA	0.0000	0.0000	0.0000	0.0000	0.0000	0.0000	0.0000	0.0000

パス図

変数情報

ﾓﾃﾞﾙﾌｧｲﾙ作成

警告・エラー 収束過程 分析情報

For Help, press F1

ここに入力して検索

20:31
2019/03/30

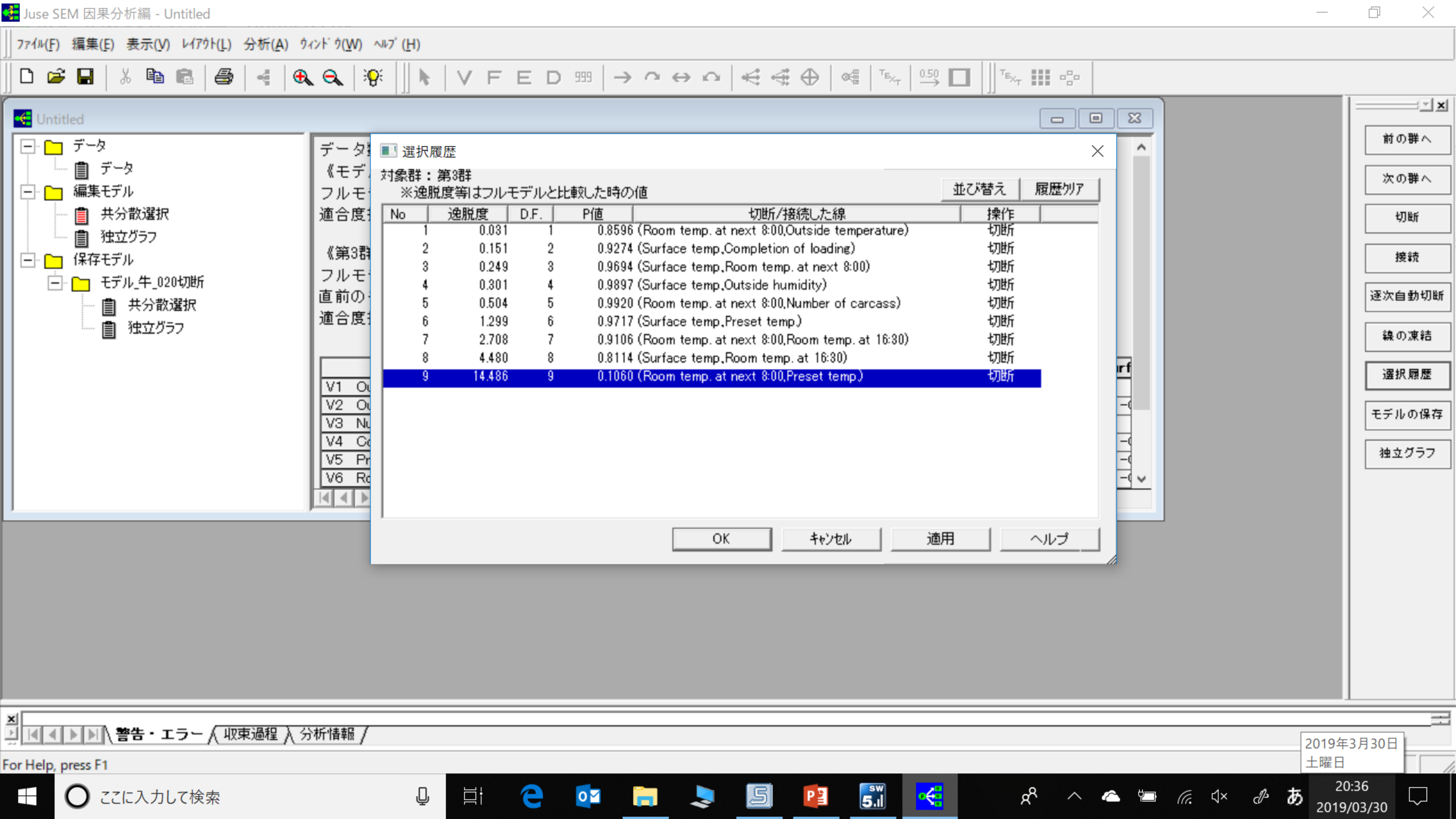

No	逸脱度	D.F.	P値	切断/接続した線	操作
1	0.031	1	0.8596	(Room temp. at next 8:00,Outside temperature)	切断
2	0.151	2	0.9274	(Surface temp.,Completion of loading)	切断
3	0.249	3	0.9694	(Surface temp.,Room temp. at next 8:00)	切断
4	0.301	4	0.9897	(Surface temp.,Outside humidity)	切断
5	0.504	5	0.9920	(Room temp. at next 8:00,Number of carcass)	切断
6	1.299	6	0.9717	(Surface temp.,Preset temp.)	切断
7	2.708	7	0.9106	(Room temp. at next 8:00,Room temp. at 16:30)	切断
8	4.480	8	0.8114	(Surface temp.,Room temp. at 16:30)	切断
9	14.486	9	0.1060	(Room temp. at next 8:00,Preset temp.)	切断

Juse SEM 因果分析編 - [Untitled]

データ数：44

《モデル全体》

フルモデルとの比較　：逸脱度＝8.622　自由度＝18　P値＝0.9679

適合度指標　：NFI＝0.947

《第3群》

フルモデルとの比較　：逸脱度＝4.081　自由度＝8　P値＝0.8497

直前のモデルとの比較　：逸脱度＝ー　自由度＝ー　P値＝ー

適合度指標　：GFI＝0.978　AGFI＝0.902　NFI＝0.975　SRMR＝0.027

下三角：偏相関係数　上三角：相関係数の残差

	Outside te	Outside hu	Number of	Completion	Preset tem	Room temp	Room temp	Surface te
V1 Outside temper	***						0.03303	
V2 Outside humidi		***						−0.02057
V3 Number of carc			***					
V4 Completion of I				***				−0.04636
V5 Preset temp.					***		0.02885	−0.04656
V6 Room temp. at						***	−0.08442	−0.11077
V7 Room temp. at	0.00001	−0.26037	−0.17739	−0.25563	−0.00000	−0.00001	***	−0.02557
V8 Surface temp.	−0.36976	0.00000	0.31887	0.00000	−0.00000	0.00001	−0.00000	***

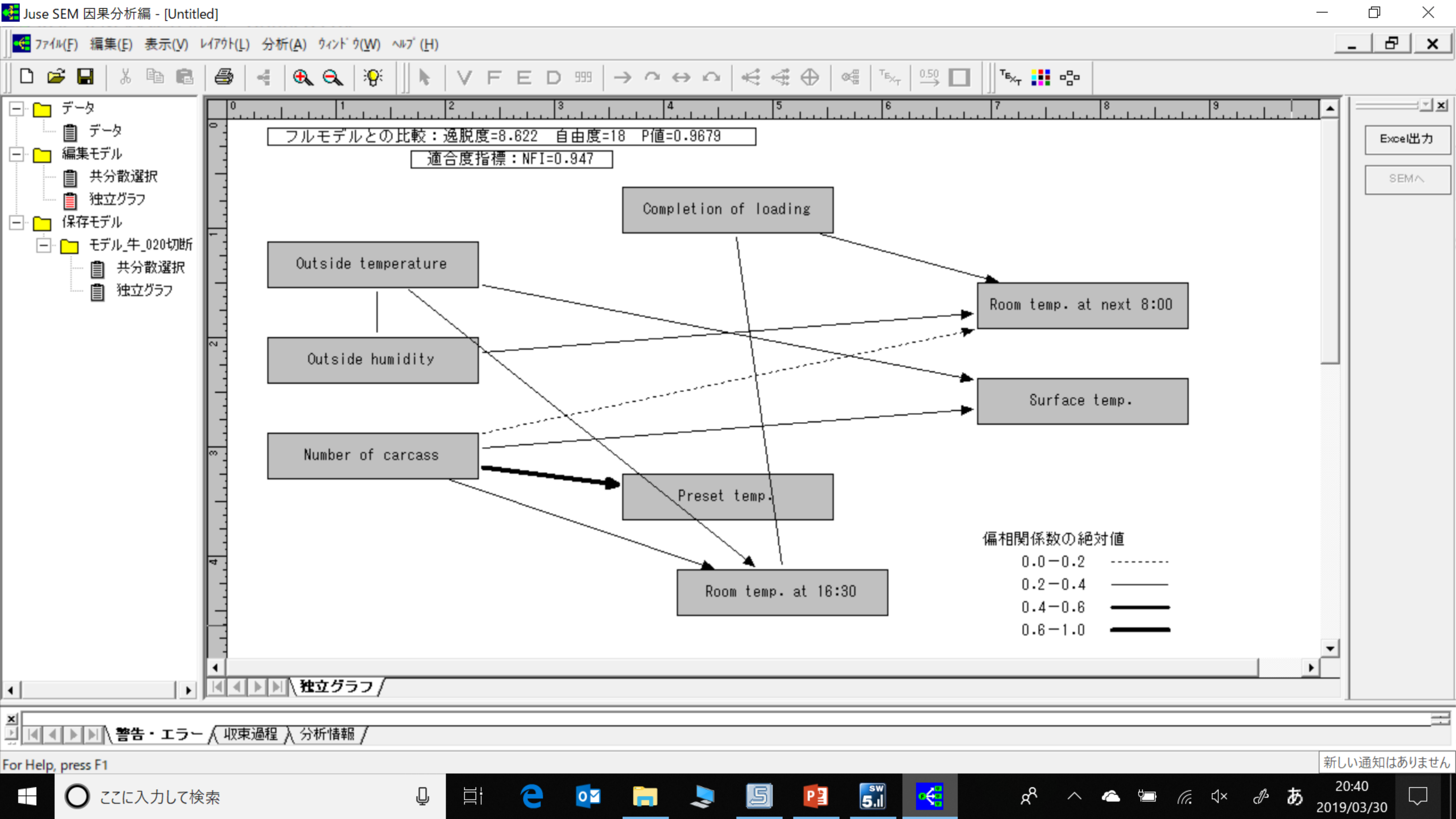

Juse SEM 因果分析編 - [Untitled]
ファイル(F) 編集(E) 表示(V) レイアウト(L) 分析(A) ウィンドウ(W) ヘルプ(H)
データ
データ
編集モデル
共分散選択
独立グラフ
保存モデル
モデル_牛_020切断
共分散選択
独立グラフ
フルモデルとの比較：逸脱度=8.622 自由度=18 P値=0.9679
適合度指標：NFI=0.947
Completion of loading
Outside temperature
Room temp. at next 8:00
Outside humidity
Surface temp.
Number of carcass
Preset temp.
Room temp. at 16:30
偏相関係数の絶対値
0.0−0.2
0.2−0.4
0.4−0.6
0.6−1.0
Excel出力
SEMへ
独立グラフ
警告・エラー
収束過程
分析情報
For Help, press F1
新しい通知はありません
ここに入力して検索
20:40
2019/03/30

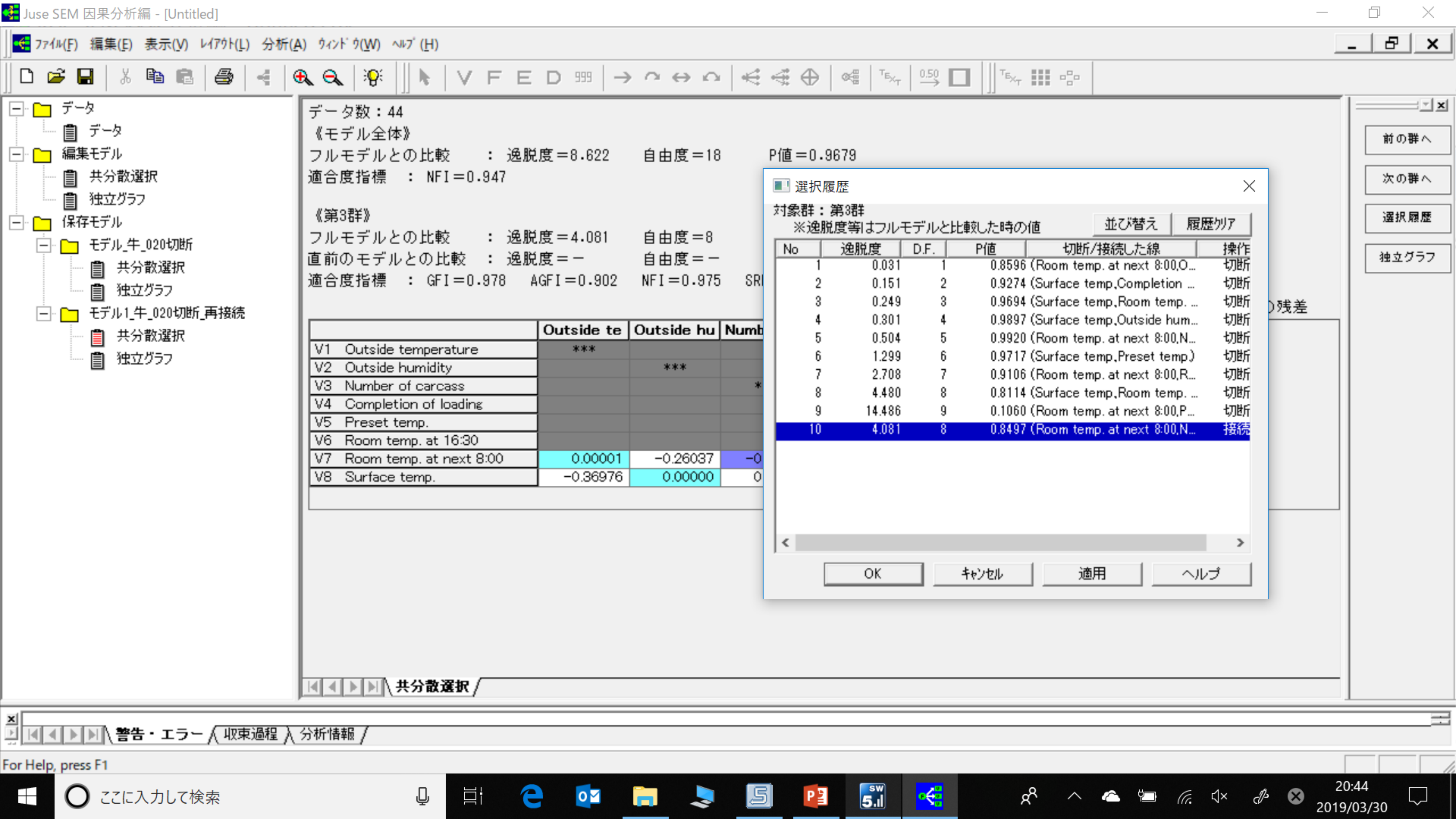
Juse SEM 因果分析編 - [Untitled]
ﾌｧｲﾙ(F) 編集(E) 表示(V) ﾚｲｱｳﾄ(L) 分析(A) ｳｨﾝﾄﾞｳ(W) ﾍﾙﾌﾟ(H)
データ
データ
編集モデル
共分散選択
独立グラフ
保存モデル
モデル_牛_020切断
共分散選択
独立グラフ
モデル1_牛_020切断_再接続
共分散選択
独立グラフ
データ数：44
《モデル全体》
フルモデルとの比較　：　逸脱度＝8.622　自由度＝18　P値＝0.9679
適合度指標　：　NFI＝0.947
《第3群》
フルモデルとの比較　：　逸脱度＝4.081　自由度＝8
直前のモデルとの比較　：　逸脱度＝－　自由度＝－
適合度指標　：　GFI＝0.978　AGFI＝0.902　NFI＝0.975　SR
Outside te
Outside hu
Numb
V1 Outside temperature
V2 Outside humidity
V3 Number of carcass
V4 Completion of loading
V5 Preset temp.
V6 Room temp. at 16:30
V7 Room temp. at next 8:00
V8 Surface temp.
***
***
0.00001
-0.26037
-0.36976
0.00000
残差
共分散選択
選択履歴
対象群：第3群
※逸脱度等はフルモデルと比較した時の値
並び替え
履歴ｸﾘｱ
No 逸脱度 D.F. P値 切断/接続した線 操作
1 0.031 1 0.8596 (Room temp. at next 8:00,O... 切断
2 0.151 2 0.9274 (Surface temp.,Completion ... 切断
3 0.249 3 0.9694 (Surface temp.,Room temp. ... 切断
4 0.301 4 0.9897 (Surface temp.,Outside hum... 切断
5 0.504 5 0.9920 (Room temp. at next 8:00,N... 切断
6 1.299 6 0.9717 (Surface temp.,Preset temp.) 切断
7 2.708 7 0.9106 (Room temp. at next 8:00,R... 切断
8 4.480 8 0.8114 (Surface temp.,Room temp. ... 切断
9 14.486 9 0.1060 (Room temp. at next 8:00,P... 切断
10 4.081 8 0.8497 (Room temp. at next 8:00,N... 接続
OK
ｷｬﾝｾﾙ
適用
ﾍﾙﾌﾟ
前の群へ
次の群へ
選択履歴
独立グラフ
警告・エラー
収束過程
分析情報
For Help, press F1
ここに入力して検索
20:44
2019/03/30

Juse SEM 因果分析編 - モデル相関係数行列_第3群.ess

ﾌｧｲﾙ(F) 編集(E) 表示(V) ﾃﾞｰﾀ(D) 分析(A) ｳｨﾝﾄﾞｳ(W) ﾍﾙﾌﾟ(H)

標本相関係数行列.ess

	Outside	Outside	Number o	Completi	Preset t	Room te	Room te	Surface
Outsid	1.0000	0.3059	0.0976	−0.2262	−0.1004	−0.2297	−0.0291	−0.2527
Outsid	0.3059	1.0000	0.0253	−0.1984	−0.0422	−0.0626	−0.2021	−0.1009
Numb	0.0976	0.0253	1.0000	0.1498	−0.9156	0.5443	−0.4748	0.6334
Compl	−0.2262	−0.1984	0.1498	1.0000	−0.1055	0.2565	−0.2515	0.1250
Prese	−0.1004	−0.0422	−0.9156	−0.1055	1.0000	−0.4395	0.4607	−0.6230
Room	−0.2297	−0.0626	0.5443	0.2565	−0.4395	1.0000	−0.3656	0.3238
Room	−0.0291	−0.2021	−0.4748	−0.2515	0.4607	−0.3656	1.0000	−0.3213
Surfa	−0.2527	−0.1009	0.6334	0.1250	−0.6230	0.3238	−0.3213	1.0000
STD_D	1.0000	1.0000	1.0000	1.0000	1.0000	1.0000	1.0000	1.0000
_MEA	0.0000	0.0000	0.0000	0.0000	0.0000	0.0000	0.0000	0.0000

モデル相関係数行列_第3群.ess

	Outside	Outside	Number o	Completi	Preset t	Room te	Room te	Surface
Outsid	1.0000	0.3059	0.0976	−0.2262	−0.1004	−0.2297	−0.0621	−0.2527
Outsid	0.3059	1.0000	0.0253	−0.1984	−0.0422	−0.0626	−0.2021	−0.0803
Numb	0.0976	0.0253	1.0000	0.1498	−0.9156	0.5443	−0.4748	0.6334
Compl	−0.2262	−0.1984	0.1498	1.0000	−0.1055	0.2565	−0.2515	0.1714
Prese	−0.1004	−0.0422	−0.9156	−0.1055	1.0000	−0.4395	0.4319	−0.5764
Room	−0.2297	−0.0626	0.5443	0.2565	−0.4395	1.0000	−0.2812	0.4346
Room	−0.0621	−0.2021	−0.4748	−0.2515	0.4319	−0.2812	1.0000	−0.2957
Surfa	−0.2527	−0.0803	0.6334	0.1714	−0.5764	0.4346	−0.2957	1.0000
STD_D	1.0000	1.0000	1.0000	1.0000	1.0000	1.0000	1.0000	1.0000
_MEA	0.0000	0.0000	0.0000	0.0000	0.0000	0.0000	0.0000	0.0000

パス図

変数情報

ﾓﾃﾞﾙﾌｧｲﾙ作成

警告・エラー 収束過程 分析情報

For Help, press F1

ここに入力して検索

20:46
2019/03/30
