# Supplementary material for: Dataset on causality analysis of chilling process in beef and pork carcasses using graphical modeling
Source: Data Brief. 2020 Jul 25;32:106075. doi: 10.1016/j.dib.2020.106075 (PMC7424210; doi:10.1016/j.dib.2020.106075)
Supplement: Supplementary file 4 — Supplementary Fig. S1b. The data processing of covariance selection of pork. [file mmc4.pdf]

Supplementary Fig. S1b.
The data processing of covariance selection of pork

Kuzuoka et al.

Sequence 1.

Partial correlation: less than 0.10

NFI: 0.90 or more

Juse SEM 因果分析編 - [Untitles]

ﾌｧｲﾙ(F) 編集(E) 表示(V) ﾚｲｱｳﾄ(L) 分析(A) ｳｨﾝﾄﾞｳ(W) ﾍﾙﾌﾟ(H)

- データ
  - データ
- 編集モデル
  - 共分散選択
  - 独立グラフ
- 保存モデル

	Outside	Outside	Number o	Carcass	Completi	Preset t	Room te	Room te	Surface	Inside t
Outsid	1.0000	0.3892	−0.0157	0.0254	−0.0386	0.0719	−0.0956	0.0877	−0.4842	−0.0978
Outsid	0.3892	1.0000	−0.0683	0.0491	0.1453	0.1702	−0.0166	0.2089	−0.3783	0.0685
Numb	−0.0157	−0.0683	1.0000	0.5535	0.4880	−0.8188	0.4692	−0.6878	−0.0844	0.3874
Carca	0.0254	0.0491	0.5535	1.0000	0.7496	−0.6653	0.6274	−0.5274	−0.0758	0.4847
Compl	−0.0386	0.1453	0.4880	0.7496	1.0000	−0.5847	0.5963	−0.4201	−0.0233	0.3818
Prese	0.0719	0.1702	−0.8188	−0.6653	−0.5847	1.0000	−0.4351	0.7993	−0.0718	−0.4696
Room	−0.0956	−0.0166	0.4692	0.6274	0.5963	−0.4351	1.0000	−0.3621	−0.1259	0.3588
Room	0.0877	0.2089	−0.6878	−0.5274	−0.4201	0.7993	−0.3621	1.0000	−0.0628	−0.4646
Surfa	−0.4842	−0.3783	−0.0844	−0.0758	−0.0233	−0.0718	−0.1259	−0.0628	1.0000	0.3434
Inside	−0.0978	0.0685	0.3874	0.4847	0.3818	−0.4696	0.3588	−0.4646	0.3434	1.0000
STD_D	1.0000	1.0000	1.0000	1.0000	1.0000	1.0000	1.0000	1.0000	1.0000	1.0000
_MEA	0.0000	0.0000	0.0000	0.0000	0.0000	0.0000	0.0000	0.0000	0.0000	0.0000

変数情報

ｻﾝﾌﾟﾙのﾏｽｸ

共分散選択

データ

警告・エラー 収束過程 分析情報

For Help, press F1

ここに入力して検索

22:18
2019/03/30

Juse SEM 因果分析編 - [Untitled]

ﾌｧｲﾙ(F) 編集(E) 表示(V) ﾚｲｱｳﾄ(L) 分析(A) ｳｨﾝﾄﾞｳ(W) ﾍﾙﾌﾟ(H)

- データ
  - データ
- 編集モデル
  - 共分散選択
  - 独立グラフ
- 保存モデル

データ数：44

《モデル全体》

フルモデルとの比較　：逸脱度＝ー　自由度＝ー　P値＝ー

適合度指標　：NFI＝1.000

《第1群》

フルモデルとの比較　：逸脱度＝ー　自由度＝ー　P値＝ー

直前のモデルとの比較　：逸脱度＝ー　自由度＝ー　P値＝ー

適合度指標　：GFI＝1.000　AGFI＝1.000　NFI＝1.000　SRMR＝0.000

下三角：偏相関係数　上三角：相関係数の残差

	Outside te	Outside hu	Number of	Carcass af	Completion	Preset tem	Room temp	Room temp	Surface te	Inside tem
V1 Outside temperature	***									
V2 Outside humidity	0.38908	***								
V3 Number of carcass	0.01184	-0.06752	***							
V4 Carcass after noon				***						
V5 Completion of loading					***					
V6 Preset temp.						***				
V7 Room temp. at 16:30							***			
V8 Room temp. at next 8:00								***		
V9 Surface temp.									***	
V10 Inside temp.										***

前の群へ
次の群へ
切断
接続
逐次自動切断
線の凍結
選択履歴
モデルの保存
独立グラフ

共分散選択

警告・エラー　収束過程　分析情報

For Help, press F1

ここに入力して検索

22:19
2019/03/30

Juse SEM 因果分析編 - [Untitled]

ﾌｧｲﾙ(F) 編集(E) 表示(V) ﾚｲｱｳﾄ(L) 分析(A) ｳｨﾝﾄﾞｳ(W) ﾍﾙﾌﾟ(H)

- データ
  - データ
- 編集モデル
  - 共分散選択
  - 独立グラフ
- 保存モデル

データ数：44

《モデル全体》

フルモデルとの比較　：逸脱度＝0.212　自由度＝2　P値＝0.8995

適合度指標　：NFI＝0.999

《第1群》

フルモデルとの比較　：逸脱度＝0.212　自由度＝2　P値＝0.8995

直前のモデルとの比較　：逸脱度＝0.206　自由度＝1　P値＝0.6501

適合度指標　：GFI＝0.997　AGFI＝0.990　NFI＝0.972　SRMR＝0.029

下三角：偏相関係数　上三角：相関係数の残差

	Outside te	Outside hu	Number of	Carcass af	Completion	Preset tem	Room temp	Room temp	Surface te	Inside tem
V1 Outside temperature	***		-0.01570							
V2 Outside humidity	0.38920	***	-0.06829							
V3 Number of carcass	-0.00000	-0.00001	***							
V4 Carcass after noon				***						
V5 Completion of loading					***					
V6 Preset temp.						***				
V7 Room temp. at 16:30							***			
V8 Room temp. at next 8:00								***		
V9 Surface temp.									***	
V10 Inside temp.										***

前の群へ

次の群へ

切断

接続

逐次自動切断

線の凍結

選択履歴

モデルの保存

独立グラフ

共分散選択

警告・エラー　収束過程　分析情報

For Help, press F1

JUSE Package Software

ここに入力して検索

22:20
2019/03/30

Juse SEM 因果分析編 - [Untitled]

ﾌｧｲﾙ(F) 編集(E) 表示(V) ﾚｲｱｳﾄ(L) 分析(A) ｳｨﾝﾄﾞｳ(W) ﾍﾙﾌﾟ(H)

- データ
  - データ
- 編集モデル
  - 共分散選択
  - 独立グラフ
- 保存モデル

データ数：44

《モデル全体》

フルモデルとの比較　：逸脱度＝0.212　自由度＝2　P値＝0.8995

適合度指標　：NFI＝0.999

《第2群》

フルモデルとの比較　：逸脱度＝－　自由度＝－　P値＝－

直前のモデルとの比較　：逸脱度＝－　自由度＝－　P値＝－

適合度指標　：GFI＝1.000　AGFI＝1.000　NFI＝1.000　SRMR＝0.000

下三角：偏相関係数　上三角：相関係数の残差

	Outside te	Outside hu	Number of	Carcass af	Completion	Preset tem	Room temp	Room temp	Surface te	Inside tem
V1 Outside temperature	***									
V2 Outside humidity		***								
V3 Number of carcass			***							
V4 Carcass after noon	0.13578	0.03666	−0.08989	***						
V5 Completion of loading	−0.13398	0.28410	−0.08218	0.43455	***					
V6 Preset temp.	0.02540	0.29253	−0.73377	−0.33900	−0.21947	***				
V7 Room temp. at 16:30	−0.10123	−0.10140	0.26789	0.34057	0.25824	0.22667	***			
V8 Room temp. at next 8:00								***		
V9 Surface temp.									***	
V10 Inside temp.										***

前の群へ

次の群へ

切断

接続

逐次自動切断

線の凍結

選択履歴

モデルの保存

独立グラフ

共分散選択

警告・エラー　収束過程　分析情報

For Help, press F1

新しい通知はありません

ここに入力して検索

22:20
2019/03/30

Juse SEM 因果分析編 - [Untitled]

ﾌｧｲﾙ(F)　編集(E)　表示(V)　ﾚｲｱｳﾄ(L)　分析(A)　ｳｨﾝﾄﾞｳ(W)　ﾍﾙﾌﾟ(H)

- データ
  - データ
- 編集モデル
  - 共分散選択
  - 独立グラフ
- 保存モデル

データ数：44

《モデル全体》

フルモデルとの比較　：逸脱度＝1.011　自由度＝6　P値＝0.9852

適合度指標　：NFI＝0.996

《第2群》

フルモデルとの比較　：逸脱度＝0.799　自由度＝4　P値＝0.9386

直前のモデルとの比較　：逸脱度＝0.399　自由度＝1　P値＝0.5276

適合度指標　：GFI＝0.995　AGFI＝0.964　NFI＝0.995　SRMR＝0.013

下三角：偏相関係数　上三角：相関係数の残差

	Outside te	Outside hu	Number of	Carcass af	Completion	Preset tem	Room temp	Room temp	Surface te	Inside tem
V1　Outside temperature	***					0.00880				
V2　Outside humidity		***		−0.00229			−0.06229			
V3　Number of carcass			***	−0.02609						
V4　Carcass after noon	0.13633	−0.00000	0.00000	***						
V5　Completion of loading	−0.13050	0.26394	−0.11199	0.45286	***					
V6　Preset temp.	−0.00000	0.28383	−0.71780	−0.27440	−0.24009	***				
V7　Room temp. at 16:30	−0.13196	−0.00001	0.22938	0.31967	0.24017	0.17558	***			
V8　Room temp. at next 8:00								***		
V9　Surface temp.									***	
V10　Inside temp.										***

共分散選択

前の群へ　次の群へ　切断　接続　逐次自動切断　線の凍結　選択履歴　モデルの保存　独立グラフ

警告・エラー　収束過程　分析情報

For Help, press F1

ここに入力して検索　22:21　2019/03/30

Juse SEM 因果分析編 - [Untitled]

ﾌｧｲﾙ(F) 編集(E) 表示(V) ﾚｲｱｳﾄ(L) 分析(A) ｳｨﾝﾄﾞｳ(W) ﾍﾙﾌﾟ(H)

- データ
  - データ
- 編集モデル
  - 共分散選択
  - 独立グラフ
- 保存モデル

データ数：44

《モデル全体》

フルモデルとの比較　：逸脱度＝1.011　自由度＝6　P値＝0.9852

適合度指標　：NFI＝0.996

《第3群》

フルモデルとの比較　：逸脱度＝ー　自由度＝ー　P値＝ー

直前のモデルとの比較　：逸脱度＝ー　自由度＝ー　P値＝ー

適合度指標　：GFI＝1.000　AGFI＝1.000　NFI＝1.000　SRMR＝0.000

下三角：偏相関係数　上三角：相関係数の残差

	Outside te	Outside hu	Number of	Carcass af	Completion	Preset tem	Room temp	Room temp	Surface te	Inside tem
V1 Outside temperature	***									
V2 Outside humidity		***								
V3 Number of carcass			***							
V4 Carcass after noon				***						
V5 Completion of loading					***					
V6 Preset temp.						***				
V7 Room temp. at 16:30							***			
V8 Room temp. at next 8:00	0.03904	0.16817	−0.08612	0.00344	0.05744	0.47157	0.02505	***		
V9 Surface temp.	−0.38511	−0.35825	−0.17568	−0.16012	0.14593	−0.12011	−0.24969	0.12891	***	
V10 Inside temp.	0.07731	0.35517	0.04596	0.23830	−0.13366	−0.01192	0.20074	−0.23071	0.52386	***

前の群へ
次の群へ
切断
接続
逐次自動切断
線の凍結
選択履歴
モデルの保存
独立グラフ

共分散選択

警告・エラー　収束過程　分析情報

For Help, press F1

03_豚_平均

ここに入力して検索

22:21
2019/03/30

Juse SEM 因果分析編 - [Untitled]

データ数：44

《モデル全体》

フルモデルとの比較　：逸脱度＝2.427　自由度＝14　P値＝0.9997

適合度指標　：NFI＝0.990

《第3群》

フルモデルとの比較　：逸脱度＝1.416　自由度＝8　P値＝0.9940

直前のモデルとの比較　：逸脱度＝0.431　自由度＝1　P値＝0.5114

適合度指標　：GFI＝0.994　AGFI＝0.957　NFI＝0.994　SRMR＝0.014

下三角：偏相関係数　上三角：相関係数の残差

	Outside te	Outside hu	Number of	Carcass af	Completion	Preset tem	Room temp	Room temp	Surface te	Inside tem
V1 Outside temperature	***							0.00645		0.04177
V2 Outside humidity		***								
V3 Number of carcass			***					-0.03238		0.05067
V4 Carcass after noon				***				0.03493		
V5 Completion of loading					***			0.04441		
V6 Preset temp.						***				-0.02945
V7 Room temp. at 16:30							***	0.03066		
V8 Room temp. at next 8:00	-0.00000	0.20131	-0.00001	0.00000	-0.00000	0.48687	-0.00000	***		
V9 Surface temp.	-0.35221	-0.35597	-0.15631	-0.16067	0.14738	-0.13064	-0.24182	0.14279	***	
V10 Inside temp.	-0.00000	0.36436	-0.00000	0.25287	-0.14030	-0.00001	0.19143	-0.25968	0.49598	***

共分散選択

For Help, press F1

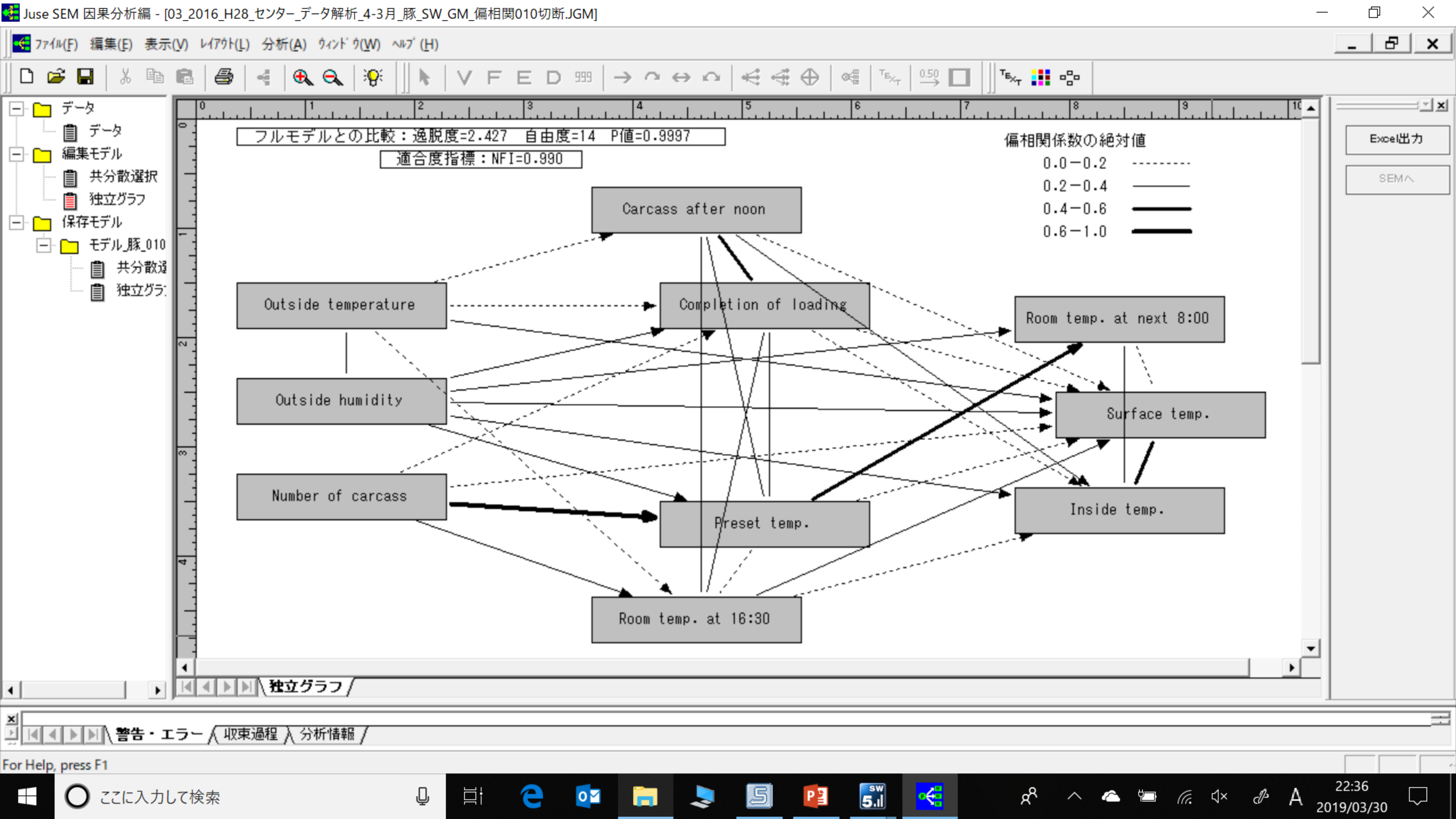
Juse SEM 因果分析編 - [03_2016_H28_センター_データ解析_4-3月_豚_SW_GM_偏相関010切断.JGM]
ﾌｧｲﾙ(F) 編集(E) 表示(V) ﾚｲｱｳﾄ(L) 分析(A) ｳｨﾝﾄﾞｳ(W) ﾍﾙﾌﾟ(H)
データ
データ
編集モデル
共分散選択
独立グラフ
保存モデル
モデル_豚_010
フルモデルとの比較：逸脱度=2.427 自由度=14 P値=0.9997
適合度指標：NFI=0.990
偏相関係数の絶対値
0.0−0.2
0.2−0.4
0.4−0.6
0.6−1.0
Carcass after noon
Outside temperature
Completion of loading
Room temp. at next 8:00
Outside humidity
Surface temp.
Number of carcass
Preset temp.
Inside temp.
Room temp. at 16:30
Excel出力
SEMへ
独立グラフ
警告・エラー
収束過程
分析情報
For Help, press F1
ここに入力して検索
22:36
2019/03/30

Sequence 2.

Partial correlation: less than 0.20

NFI: 0.90 or more

Juse SEM 因果分析編 - [Untitles]

ﾌｧｲﾙ(F) 編集(E) 表示(V) ﾚｲｱｳﾄ(L) 分析(A) ｳｨﾝﾄﾞｳ(W) ﾍﾙﾌﾟ(H)

- データ
  - データ
- 編集モデル
  - 共分散選択
  - 独立グラフ
- 保存モデル

	Outside	Outside	Number o	Carcass	Completi	Preset t	Room te	Room te	Surface	Inside t
Outsid	1.0000	0.3892	−0.0157	0.0254	−0.0386	0.0719	−0.0956	0.0877	−0.4842	−0.0978
Outsid	0.3892	1.0000	−0.0683	0.0491	0.1453	0.1702	−0.0166	0.2089	−0.3783	0.0685
Numb	−0.0157	−0.0683	1.0000	0.5535	0.4880	−0.8188	0.4692	−0.6878	−0.0844	0.3874
Carca	0.0254	0.0491	0.5535	1.0000	0.7496	−0.6653	0.6274	−0.5274	−0.0758	0.4847
Compl	−0.0386	0.1453	0.4880	0.7496	1.0000	−0.5847	0.5963	−0.4201	−0.0233	0.3818
Prese	0.0719	0.1702	−0.8188	−0.6653	−0.5847	1.0000	−0.4351	0.7993	−0.0718	−0.4696
Room	−0.0956	−0.0166	0.4692	0.6274	0.5963	−0.4351	1.0000	−0.3621	−0.1259	0.3588
Room	0.0877	0.2089	−0.6878	−0.5274	−0.4201	0.7993	−0.3621	1.0000	−0.0628	−0.4646
Surfa	−0.4842	−0.3783	−0.0844	−0.0758	−0.0233	−0.0718	−0.1259	−0.0628	1.0000	0.3434
Inside	−0.0978	0.0685	0.3874	0.4847	0.3818	−0.4696	0.3588	−0.4646	0.3434	1.0000
STD_D	1.0000	1.0000	1.0000	1.0000	1.0000	1.0000	1.0000	1.0000	1.0000	1.0000
_MEA	0.0000	0.0000	0.0000	0.0000	0.0000	0.0000	0.0000	0.0000	0.0000	0.0000

変数情報

ｻﾝﾌﾟﾙのﾏｽｸ

共分散選択

データ

警告・エラー　収束過程　分析情報

For Help, press F1

ここに入力して検索

22:38
2019/03/30

Juse SEM 因果分析編 - [Untitled]

ﾌｧｲﾙ(F) 編集(E) 表示(V) ﾚｲｱｳﾄ(L) 分析(A) ｳｨﾝﾄﾞｳ(W) ﾍﾙﾌﾟ(H)

- データ
  - データ
- 編集モデル
  - 共分散選択
  - 独立グラフ
- 保存モデル

データ数：44

《モデル全体》

フルモデルとの比較　：逸脱度＝－　自由度＝－　P値＝－

適合度指標　：NFI＝1.000

《第1群》

フルモデルとの比較　：逸脱度＝－　自由度＝－　P値＝－

直前のモデルとの比較　：逸脱度＝－　自由度＝－　P値＝－

適合度指標　：GFI＝1.000　AGFI＝1.000　NFI＝1.000　SRMR＝0.000

下三角：偏相関係数　上三角：相関係数の残差

	Outside te	Outside hu	Number of	Carcass af	Completion	Preset tem	Room temp	Room temp	Surface te	Inside tem
V1 Outside temperature	***									
V2 Outside humidity	0.38908	***								
V3 Number of carcass	0.01184	-0.06752	***							
V4 Carcass after noon				***						
V5 Completion of loading					***					
V6 Preset temp.						***				
V7 Room temp. at 16:30							***			
V8 Room temp. at next 8:00								***		
V9 Surface temp.									***	
V10 Inside temp.										***

共分散選択

前の群へ

次の群へ

切断

接続

逐次自動切断

線の凍結

選択履歴

モデルの保存

独立グラフ

警告・エラー　収束過程　分析情報

For Help, press F1

ここに入力して検索

22:39
2019/03/30

Juse SEM 因果分析編 - [Untitled]

データ数：44

《モデル全体》

フルモデルとの比較　：逸脱度＝0.212　自由度＝2　P値＝0.8995

適合度指標　：NFI＝0.999

《第1群》

フルモデルとの比較　：逸脱度＝0.212　自由度＝2　P値＝0.8995

直前のモデルとの比較　：逸脱度＝0.206　自由度＝1　P値＝0.6501

適合度指標　：GFI＝0.997　AGFI＝0.990　NFI＝0.972　SRMR＝0.029

下三角：偏相関係数　上三角：相関係数の残差

	Outside te	Outside hu	Number of	Carcass af	Completion	Preset tem	Room temp	Room temp	Surface te	Inside tem
V1 Outside temperature	***		-0.01570							
V2 Outside humidity	0.38920	***	-0.06829							
V3 Number of carcass	-0.00000	-0.00001	***							
V4 Carcass after noon				***						
V5 Completion of loading					***					
V6 Preset temp.						***				
V7 Room temp. at 16:30							***			
V8 Room temp. at next 8:00								***		
V9 Surface temp.									***	
V10 Inside temp.										***

Juse SEM 因果分析編 - [Untitled]

ﾌｧｲﾙ(F) 編集(E) 表示(V) ﾚｲｱｳﾄ(L) 分析(A) ｳｨﾝﾄﾞｳ(W) ﾍﾙﾌﾟ(H)

データ
データ
編集モデル
共分散選択
独立グラフ
保存モデル

データ数：44
《モデル全体》
フルモデルとの比較　：逸脱度＝0.212　自由度＝2　P値＝0.8995
適合度指標　：NFI＝0.999

《第2群》
フルモデルとの比較　：逸脱度＝－　自由度＝－　P値＝－
直前のモデルとの比較　：逸脱度＝－　自由度＝－　P値＝－
適合度指標　：GFI＝1.000　AGFI＝1.000　NFI＝1.000　SRMR＝0.000

下三角：偏相関係数　上三角：相関係数の残差

	Outside te	Outside hu	Number of	Carcass af	Completion	Preset tem	Room temp	Room temp	Surface te	Inside tem
V1 Outside temperature	***									
V2 Outside humidity		***								
V3 Number of carcass			***							
V4 Carcass after noon	0.13578	0.03666	−0.08989	***						
V5 Completion of loading	−0.13398	0.28410	−0.08218	0.43455	***					
V6 Preset temp.	0.02540	0.29253	−0.73377	−0.33900	−0.21947	***				
V7 Room temp. at 16:30	−0.10123	−0.10140	0.26789	0.34057	0.25824	0.22667	***			
V8 Room temp. at next 8:00								***		
V9 Surface temp.									***	
V10 Inside temp.										***

共分散選択

前の群へ
次の群へ
切断
接続
逐次自動切断
線の凍結
選択履歴
モデルの保存
独立グラフ

警告・エラー　収束過程　分析情報

For Help, press F1

ここに入力して検索
22:40
2019/03/30

Juse SEM 因果分析編 - [Untitled]

ﾌｧｲﾙ(F) 編集(E) 表示(V) ﾚｲｱｳﾄ(L) 分析(A) ｳｨﾝﾄﾞｳ(W) ﾍﾙﾌﾟ(H)

データ
データ
編集モデル
共分散選択
独立グラフ
保存モデル

データ数：44
《モデル全体》
フルモデルとの比較　：逸脱度＝13.331　自由度＝15　P値＝0.5767
適合度指標　：NFI＝0.947

《第2群》
フルモデルとの比較　：逸脱度＝13.119　自由度＝13　P値＝0.4386
直前のモデルとの比較　：逸脱度＝1.787　自由度＝1　P値＝0.1812
適合度指標　：GFI＝0.932　AGFI＝0.854　NFI＝0.916　SRMR＝0.058

下三角：偏相関係数　上三角：相関係数の残差

	Outside te	Outside hu	Number of	Carcass af	Completion	Preset tem	Room temp	Room temp	Surface te	Inside tem
V1 Outside temperature	***			0.03398	−0.03216	0.05903	−0.09021			
V2 Outside humidity		***		0.08633	0.17322	0.11427	0.00677			
V3 Number of carcass			***	0.00875	0.07967		0.12744			
V4 Carcass after noon	0.00000	−0.00001	0.00000	***						
V5 Completion of loading	−0.00001	−0.00001	−0.00001	0.52525	***	−0.08600				
V6 Preset temp.	0.00001	−0.00000	−0.72837	−0.33211	0.00000	***	−0.01770			
V7 Room temp. at 16:30	−0.00001	−0.00000	−0.00001	0.29692	0.24447	0.00000	***			
V8 Room temp. at next 8:00								***		
V9 Surface temp.									***	
V10 Inside temp.										***

前の群へ
次の群へ
切断
接続
逐次自動切断
線の凍結
選択履歴
モデルの保存
独立グラフ

共分散選択

警告・エラー　収束過程　分析情報

For Help, press F1

ここに入力して検索

22:41
2019/03/30

データ数：44
《モデル全体》
フルモデルとの比較　：逸脱度＝13.331　自由度＝15　P値＝0.5767
適合度指標　：NFI＝0.947

《第3群》
フルモデルとの比較　：逸脱度＝－　自由度＝－　P値＝－
直前のモデルとの比較　：逸脱度＝－　自由度＝－　P値＝－
適合度指標　：GFI＝1.000　AGFI＝1.000　NFI＝1.000　SRMR＝0.000

下三角：偏相関係数　上三角：相関係数の残差

	Outside te	Outside hu	Number of	Carcass af	Completion	Preset tem	Room temp	Room temp	Surface te	Inside tem
V1 Outside temperature	***									
V2 Outside humidity		***								
V3 Number of carcass			***							
V4 Carcass after noon				***						
V5 Completion of loading					***					
V6 Preset temp.						***				
V7 Room temp. at 16:30							***			
V8 Room temp. at next 8:00	0.03904	0.16817	−0.08612	0.00344	0.05744	0.47157	0.02505	***		
V9 Surface temp.	−0.38511	−0.35825	−0.17568	−0.16012	0.14593	−0.12011	−0.24969	0.12891	***	
V10 Inside temp.	0.07731	0.35517	0.04596	0.23830	−0.13366	−0.01192	0.20074	−0.23071	0.52386	***

Juse SEM 因果分析編 - [Untitled]

ﾌｧｲﾙ(F) 編集(E) 表示(V) ﾚｲｱｳﾄ(L) 分析(A) ｳｨﾝﾄﾞｳ(W) ﾍﾙﾌﾟ(H)

データ
データ
編集モデル
共分散選択
独立グラフ
保存モデル

データ数：44
《モデル全体》
フルモデルとの比較　：逸脱度＝23.226　自由度＝31　P値＝0.8408
適合度指標　：NFI＝0.908

《第3群》
フルモデルとの比較　：逸脱度＝9.895　自由度＝16　P値＝0.8721
直前のモデルとの比較　：逸脱度＝2.536　自由度＝1　P値＝0.1113
適合度指標　：GFI＝0.958　AGFI＝0.855　NFI＝0.961　SRMR＝0.036

下三角：偏相関係数　上三角：相関係数の残差

	Outside te	Outside hu	Number of	Carcass af	Completion	Preset tem	Room temp	Room temp	Surface te	Inside tem
V1 Outside temperature	***							0.00529		0.06336
V2 Outside humidity		***						0.09775		
V3 Number of carcass			***					−0.02787	−0.10565	0.04538
V4 Carcass after noon				***				0.02087	−0.00078	0.14211
V5 Completion of loading					***			0.06957	0.04726	0.03874
V6 Preset temp.						***			0.02456	−0.08765
V7 Room temp. at 16:30							***	0.02159		
V8 Room temp. at next 8:00	−0.00000	0.00001	−0.00000	0.00000	0.00001	0.50907	0.00000	***	0.07141	
V9 Surface temp.	−0.37400	−0.30244	0.00000	0.00000	0.00001	−0.00001	−0.28834	−0.00001	***	
V10 Inside temp.	0.00000	0.29017	0.00001	0.00001	−0.00000	−0.00001	0.26470	−0.24536	0.43126	***

前の群へ
次の群へ
切断
接続
逐次自動切断
線の凍結
選択履歴
モデルの保存
独立グラフ

共分散選択

警告・エラー　収束過程　分析情報

For Help, press F1

ここに入力して検索　22:42 2019/03/30

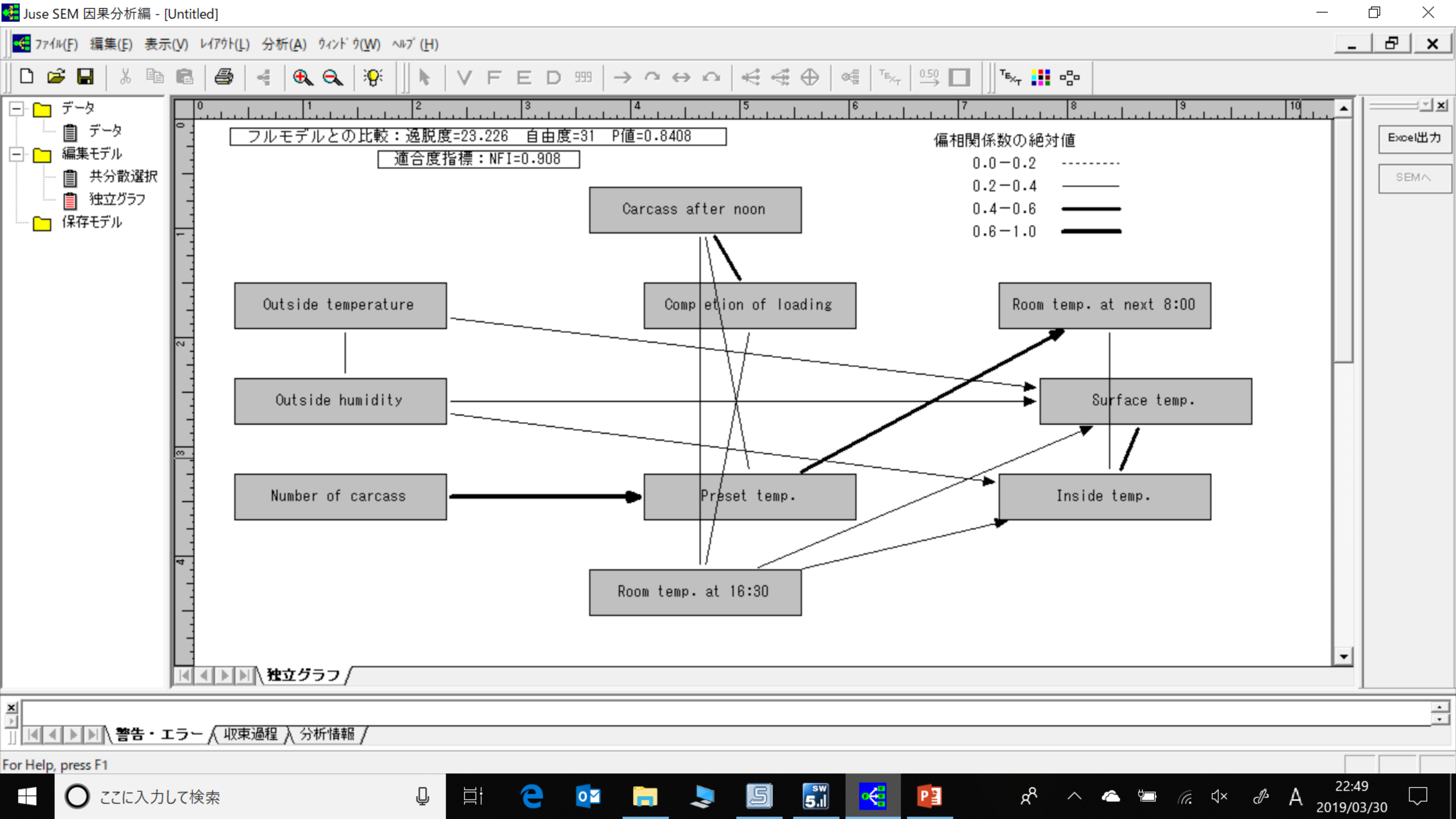
Juse SEM 因果分析編 - [Untitled]
ﾌｧｲﾙ(F) 編集(E) 表示(V) ﾚｲｱｳﾄ(L) 分析(A) ｳｨﾝﾄﾞｳ(W) ﾍﾙﾌﾟ(H)
データ
データ
編集モデル
共分散選択
独立グラフ
保存モデル
フルモデルとの比較：逸脱度=23.226　自由度=31　P値=0.8408
適合度指標：NFI=0.908
偏相関係数の絶対値
0.0－0.2
0.2－0.4
0.4－0.6
0.6－1.0
Carcass after noon
Outside temperature
Completion of loading
Room temp. at next 8:00
Outside humidity
Surface temp.
Number of carcass
Preset temp.
Inside temp.
Room temp. at 16:30
独立グラフ
Excel出力
SEMへ
警告・エラー
収束過程
分析情報
For Help, press F1
ここに入力して検索
22:49
2019/03/30

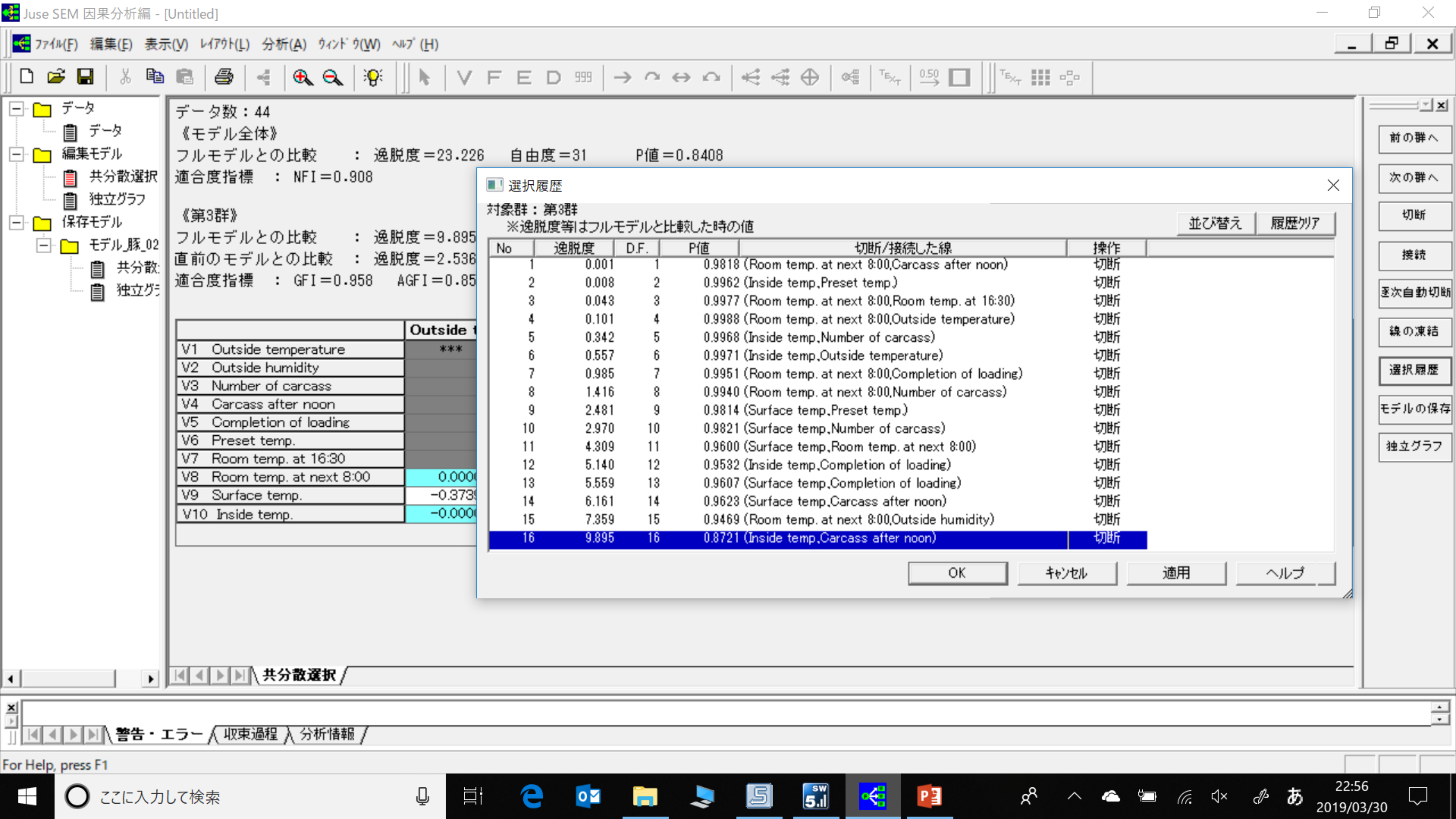
Juse SEM 因果分析編 - [Untitled]
ﾌｧｲﾙ(F) 編集(E) 表示(V) ﾚｲｱｳﾄ(L) 分析(A) ｳｨﾝﾄﾞｳ(W) ﾍﾙﾌﾟ(H)
データ
データ
編集モデル
共分散選択
独立グラフ
保存モデル
モデル_豚_02
データ数：44
《モデル全体》
フルモデルとの比較　：逸脱度＝23.226　自由度＝31　P値＝0.8408
適合度指標　：NFI＝0.908
《第3群》
フルモデルとの比較　：逸脱度＝9.895
直前のモデルとの比較　：逸脱度＝2.536
適合度指標　：GFI＝0.958　AGFI＝0.85
V1 Outside temperature
V2 Outside humidity
V3 Number of carcass
V4 Carcass after noon
V5 Completion of loading
V6 Preset temp.
V7 Room temp. at 16:30
V8 Room temp. at next 8:00
V9 Surface temp.
V10 Inside temp.
選択履歴
対象群：第3群
※逸脱度等はフルモデルと比較した時の値
並び替え
履歴クリア
No / 逸脱度 / D.F. / P値 / 切断/接続した線 / 操作
1 0.001 1 0.9818 (Room temp. at next 8:00,Carcass after noon) 切断
2 0.008 2 0.9962 (Inside temp.,Preset temp.) 切断
3 0.043 3 0.9977 (Room temp. at next 8:00,Room temp. at 16:30) 切断
4 0.101 4 0.9988 (Room temp. at next 8:00,Outside temperature) 切断
5 0.342 5 0.9968 (Inside temp.,Number of carcass) 切断
6 0.557 6 0.9971 (Inside temp.,Outside temperature) 切断
7 0.985 7 0.9951 (Room temp. at next 8:00,Completion of loading) 切断
8 1.416 8 0.9940 (Room temp. at next 8:00,Number of carcass) 切断
9 2.481 9 0.9814 (Surface temp.,Preset temp.) 切断
10 2.970 10 0.9821 (Surface temp.,Number of carcass) 切断
11 4.309 11 0.9600 (Surface temp.,Room temp. at next 8:00) 切断
12 5.140 12 0.9532 (Inside temp.,Completion of loading) 切断
13 5.559 13 0.9607 (Surface temp.,Completion of loading) 切断
14 6.161 14 0.9623 (Surface temp.,Carcass after noon) 切断
15 7.359 15 0.9469 (Room temp. at next 8:00,Outside humidity) 切断
16 9.895 16 0.8721 (Inside temp.,Carcass after noon) 切断
OK
キャンセル
適用
ヘルプ
前の群へ
次の群へ
切断
接続
逐次自動切断
線の凍結
選択履歴
モデルの保存
独立グラフ
共分散選択
警告・エラー
収束過程
分析情報
For Help, press F1
ここに入力して検索
22:56
2019/03/30

Juse SEM 因果分析編 - [Untitled]

ﾌｧｲﾙ(F) 編集(E) 表示(V) ﾚｲｱｳﾄ(L) 分析(A) ｳｨﾝﾄﾞｳ(W) ﾍﾙﾌﾟ(H)

データ
データ
編集モデル
共分散選択
独立グラフ
保存モデル
モデル_豚_02
共分散
独立グ

データ数：44
《モデル全体》
フルモデルとの比較　：逸脱度＝20.690　自由度＝30　P値＝0.8973
適合度指標　：NFI＝0.918

《第3群》
フルモデルとの比較　：逸脱度＝7.359　自由度＝15　P値＝0.9469
直前のモデルとの比較　：逸脱度＝－　自由度＝－　P値＝－
適合度指標　：GFI＝0.968　AGFI＝0.882　NFI＝0.971　SRMR＝0.029

下三角：偏相関係数　上三角：相関係数の残差

	Outside te	Outside hu	Number of	Carcass af	Completion	Preset tem	Room temp	Room temp	Surface te	Inside tem
V1 Outside temperature	***							0.01396		0.04313
V2 Outside humidity		***						0.09383		
V3 Number of carcass			***					-0.03139	-0.11637	0.02179
V4 Carcass after noon				***				0.03371	-0.06165	
V5 Completion of loading					***			0.07206	0.01351	-0.03761
V6 Preset temp.						***			0.04648	-0.03914
V7 Room temp. at 16:30							***	0.01104		
V8 Room temp. at next 8:00	0.00001	0.00000	0.00000	0.00000	0.00001	0.50870	0.00001	***	0.07502	
V9 Surface temp.	-0.38424	-0.30224	-0.00000	-0.00001	-0.00000	0.00001	-0.29590	0.00001	***	
V10 Inside temp.	-0.00000	0.27111	-0.00000	0.17095	-0.00001	-0.00001	0.17180	-0.18674	0.42992	***

前の群へ
次の群へ
切断
接続
逐次自動切断
線の凍結
選択履歴
モデルの保存
独立グラフ

共分散選択

警告・エラー　収束過程　分析情報

For Help, press F1

ここに入力して検索　23:02 2019/03/30

Juse SEM 因果分析編 - [Untitled]

データ数：44
《モデル全体》
フルモデルとの比較　：逸脱度＝19.400　自由度＝29　P値＝0.9106
適合度指標　：NFI＝0.923

《第3群》
フルモデルとの比較　：逸脱度＝6.069　自由度＝14　P値＝0.9647
直前のモデルとの比較　：逸脱度＝－　自由度＝－　P値＝－
適合度指標　：GFI＝0.973　AGFI＝0.894　NFI＝0.976　SRMR＝0.025

下三角：偏相関係数　上三角：相関係数の残差

	Outside te	Outside hu	Number of	Carcass af	Completion	Preset tem	Room temp	Room temp	Surface te	Inside tem
V1 Outside temperature	***							0.01069		0.05065
V2 Outside humidity		***						0.09557		
V3 Number of carcass			***					−0.03526		0.06305
V4 Carcass after noon				***				0.03913	−0.03388	
V5 Completion of loading					***			0.07652	0.03900	−0.03642
V6 Preset temp.						***			−0.03569	−0.05920
V7 Room temp. at 16:30							***	0.01527		
V8 Room temp. at next 8:00	0.00000	0.00001	0.00001	0.00000	0.00001	0.50857	0.00001	***	0.02003	
V9 Surface temp.	−0.37148	−0.32102	−0.09988	0.00000	0.00001	−0.00001	−0.25016	−0.00000	***	
V10 Inside temp.	0.00000	0.28352	0.00001	0.17690	0.00000	−0.00001	0.16761	−0.20552	0.45216	***

共分散選択

Juse SEM 因果分析編 - [Untitled]

データ数：44

《モデル全体》

フルモデルとの比較　：逸脱度＝19.400　自由度＝29　P値＝0.9106

適合度指標　：NFI＝0.923

《第3群》

フルモデルとの比[illegible]

直前のモデルとの[illegible]

適合度指標　：GF[illegible]

V1 Outside temper[illegible]
V2 Outside humidi[illegible]
V3 Number of carc[illegible]
V4 Carcass after [illegible]
V5 Completion of [illegible]
V6 Preset temp.
V7 Room temp. at [illegible]
V8 Room temp. at [illegible]
V9 Surface temp.
V10 Inside temp.

選択履歴

対象群：第3群

※逸脱度等はフルモデルと比較した時の値

No	逸脱度	D.F.	P値	切断/接続した線	操作
1	0.001	1	0.9818	(Room temp. at next 8:00,Carcass after noon)	切断
2	0.008	2	0.9962	(Inside temp.,Preset temp.)	切断
3	0.043	3	0.9977	(Room temp. at next 8:00,Room temp. at 16:30)	切断
4	0.101	4	0.9988	(Room temp. at next 8:00,Outside temperature)	切断
5	0.342	5	0.9968	(Inside temp.,Number of carcass)	切断
6	0.557	6	0.9971	(Inside temp.,Outside temperature)	切断
7	0.985	7	0.9951	(Room temp. at next 8:00,Completion of loading)	切断
8	1.416	8	0.9940	(Room temp. at next 8:00,Number of carcass)	切断
9	2.481	9	0.9814	(Surface temp.,Preset temp.)	切断
10	2.970	10	0.9821	(Surface temp.,Number of carcass)	切断
11	4.309	11	0.9600	(Surface temp.,Room temp. at next 8:00)	切断
12	5.140	12	0.9532	(Inside temp.,Completion of loading)	切断
13	5.559	13	0.9607	(Surface temp.,Completion of loading)	切断
14	6.161	14	0.9623	(Surface temp.,Carcass after noon)	切断
15	7.359	15	0.9469	(Room temp. at next 8:00,Outside humidity)	切断
16	9.895	16	0.8721	(Inside temp.,Carcass after noon)	切断
17	7.359	15	0.9469	(Inside temp.,Carcass after noon)	接続
18	6.069	14	0.9647	(Surface temp.,Number of carcass)	接続

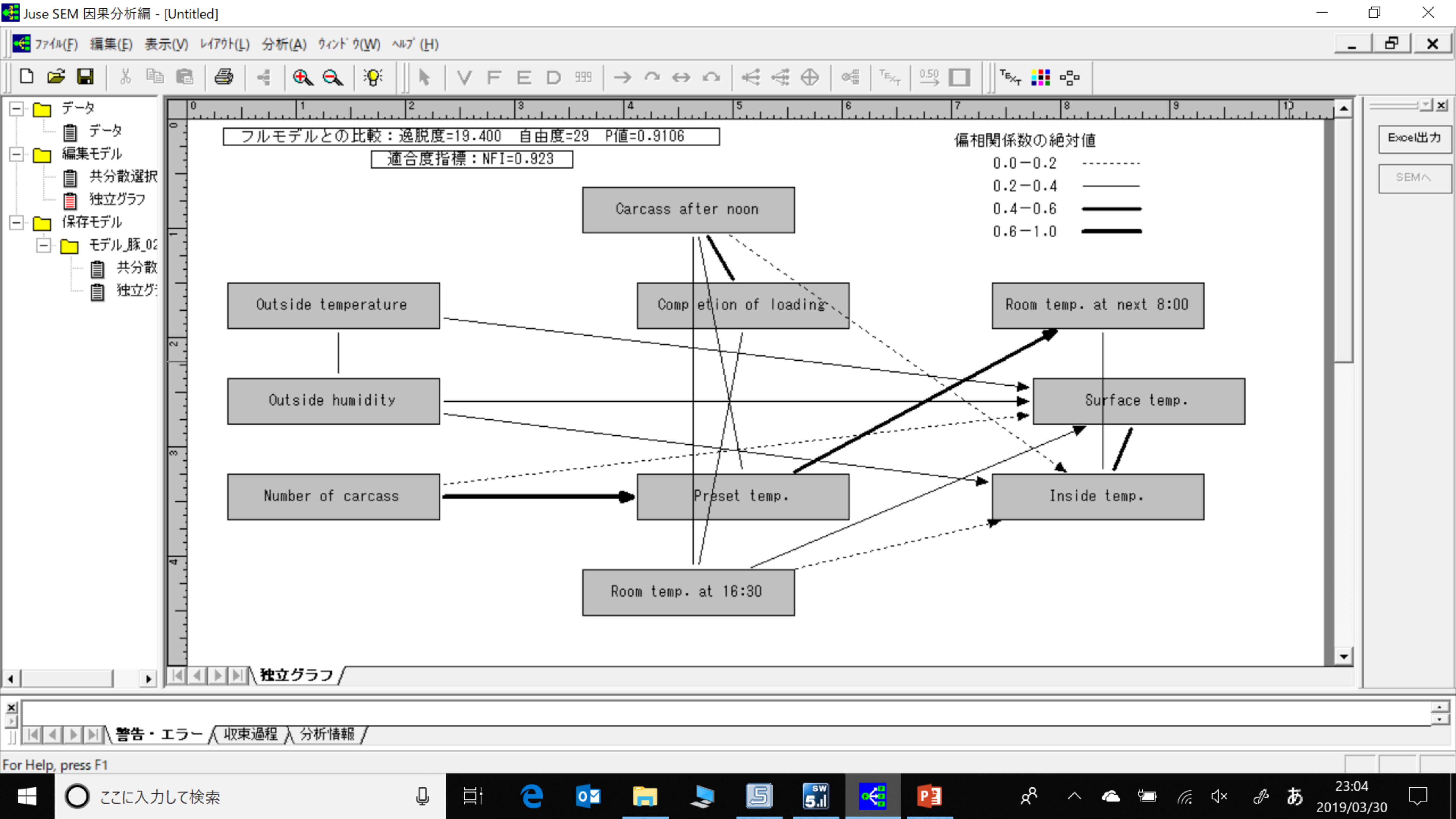
Juse SEM 因果分析編 - [Untitled]
ﾌｧｲﾙ(F) 編集(E) 表示(V) ﾚｲｱｳﾄ(L) 分析(A) ｳｨﾝﾄﾞｳ(W) ﾍﾙﾌﾟ(H)
データ
データ
編集モデル
共分散選択
独立グラフ
保存モデル
モデル_豚_02
共分散
独立グ
フルモデルとの比較：逸脱度=19.400　自由度=29　P値=0.9106
適合度指標：NFI=0.923
偏相関係数の絶対値
0.0−0.2
0.2−0.4
0.4−0.6
0.6−1.0
Carcass after noon
Outside temperature
Completion of loading
Room temp. at next 8:00
Outside humidity
Surface temp.
Number of carcass
Preset temp.
Inside temp.
Room temp. at 16:30
Excel出力
SEMへ
独立グラフ
警告・エラー
収束過程
分析情報
For Help, press F1
ここに入力して検索
23:04
2019/03/30

Juse SEM 因果分析編 - モデル相関係数行列_第3群.ess

ﾌｧｲﾙ(F) 編集(E) 表示(V) ﾃﾞｰﾀ(D) 分析(A) ｳｨﾝﾄﾞｳ(W) ﾍﾙﾌﾟ(H)

標本相関係数行列.ess

	Outside	Outside	Number o	Carcass	Completi	Preset t	Room te	Room te	Surface	Inside t
Outsid	1.0000	0.3892	−0.0157	0.0254	−0.0386	0.0719	−0.0956	0.0877	−0.4842	−0.0978
Outsid	0.3892	1.0000	−0.0683	0.0491	0.1453	0.1702	−0.0166	0.2089	−0.3783	0.0685
Numb	−0.0157	−0.0683	1.0000	0.5535	0.4880	−0.8188	0.4692	−0.6878	−0.0844	0.3874
Carca	0.0254	0.0491	0.5535	1.0000	0.7496	−0.6653	0.6274	−0.5274	−0.0758	0.4847
Compl	−0.0386	0.1453	0.4880	0.7496	1.0000	−0.5847	0.5963	−0.4201	−0.0233	0.3818
Prese	0.0719	0.1702	−0.8188	−0.6653	−0.5847	1.0000	−0.4351	0.7993	−0.0718	−0.4696
Room	−0.0956	−0.0166	0.4692	0.6274	0.5963	−0.4351	1.0000	−0.3621	−0.1259	0.3588
Room	0.0877	0.2089	−0.6878	−0.5274	−0.4201	0.7993	−0.3621	1.0000	−0.0628	−0.4646
Surfa	−0.4842	−0.3783	−0.0844	−0.0758	−0.0233	−0.0718	−0.1259	−0.0628	1.0000	0.3434
Inside	−0.0978	0.0685	0.3874	0.4847	0.3818	−0.4696	0.3588	−0.4646	0.3434	1.0000
STD_D	1.0000	1.0000	1.0000	1.0000	1.0000	1.0000	1.0000	1.0000	1.0000	1.0000
_MEA	0.0000	0.0000	0.0000	0.0000	0.0000	0.0000	0.0000	0.0000	0.0000	0.0000

モデル相関係数行列_第3群.ess

	Outside	Outside	Number o	Carcass	Completi	Preset t	Room te	Room te	Surface	Inside t
Outsid	1.0000	0.3892	−0.0157	0.0254	−0.0386	0.0719	−0.0956	0.0770	−0.4842	−0.1484
Outsid	0.3892	1.0000	−0.0683	0.0491	0.1453	0.1702	−0.0166	0.1133	−0.3783	0.0685
Numb	−0.0157	−0.0683	1.0000	0.5535	0.4880	−0.8188	0.4692	−0.6525	−0.0844	0.3243
Carca	0.0254	0.0491	0.5535	1.0000	0.7496	−0.6653	0.6274	−0.5665	−0.0419	0.4847
Compl	−0.0386	0.1453	0.4880	0.7496	1.0000	−0.5847	0.5963	−0.4966	−0.0623	0.4182
Prese	0.0719	0.1702	−0.8188	−0.6653	−0.5847	1.0000	−0.4351	0.7993	−0.0361	−0.4104
Room	−0.0956	−0.0166	0.4692	0.6274	0.5963	−0.4351	1.0000	−0.3774	−0.1259	0.3588
Room	0.0770	0.1133	−0.6525	−0.5665	−0.4966	0.7993	−0.3774	1.0000	−0.0828	−0.4646
Surfa	−0.4842	−0.3783	−0.0844	−0.0419	−0.0623	−0.0361	−0.1259	−0.0828	1.0000	0.3434
Inside	−0.1484	0.0685	0.3243	0.4847	0.4182	−0.4104	0.3588	−0.4646	0.3434	1.0000
STD_D	1.0000	1.0000	1.0000	1.0000	1.0000	1.0000	1.0000	1.0000	1.0000	1.0000
_MEA	0.0000	0.0000	0.0000	0.0000	0.0000	0.0000	0.0000	0.0000	0.0000	0.0000

パス図

変数情報

ﾃﾞﾙﾌｧｲﾙ作成

警告・エラー 収束過程 分析情報

For Help, press F1

ここに入力して検索

23:07
2019/03/30
